# Effects of Dietary Approaches and Exercise Interventions on Gestational Diabetes Mellitus: A Systematic Review and Bayesian Network Meta-analysis

**DOI:** 10.1016/j.advnut.2024.100330

**Published:** 2024-10-29

**Authors:** Liang Zhang, Fang Wang, Syoichi Tashiro, Peng Ju Liu

**Affiliations:** 1 Department of Rehabilitation Medicine, Kyorin University Faculty of Medicine, Tokyo, Japan; 2 Department of Clinical Nutrition, Department of Health Medicine, Peking Union Medical College Hospital, Chinese Academy of Medical Science and Peking Union Medical College, Beijing, PR China; 3 Department of Rehabilitation Medicine, Keio University School of Medicine, Tokyo, Japan

**Keywords:** perinatal complications, dietary pattern, lifestyle modification, physical activity, medical nutrition therapy

## Abstract

Although lifestyle interventions are recommended as the frontline therapeutic strategy for women with gestational diabetes mellitus (GDM), the optimal dietary regimen or form of exercise has yet to be definitively established. We aimed to compare the effectiveness of lifestyle therapies for GDM. Four databases (PubMed, Web of Science, EMBASE, and Cochrane Library) were systematically searched by multiple researchers for randomized controlled trials (RCTs). RCTs comparing lifestyle therapies to treat GDM with control or another treatment were included. Data extraction and synthesis were performed, estimating mean differences (MDs) or relative risk (RR) through pair-wise and network meta-analysis with a randomized or fixed-effects model when appropriate. The primary outcomes were maternal glucose control, birth weight of newborns, macrosomia and preterm birth rate, and rate of need for insulin therapy. In total, 39 trials with information obtained from 2712 women assessed 15 treatments. After sensitivity analysis, we confirmed the dietary approaches to stop hypertension (DASH) diet [MD: −11.52; 95% credible intervals (CrIs): −14.01, −9.07, very low certainty of evidence (CoE)] and low glycemic index (GI) diets (MD: −6.3; 95% CrI: −9.9, −2.7, low CoE) have shown significant advantages in fasting plasma glucose and 2-h postprandial glucose control, respectively. Furthermore, the DASH diet and resistance exercise reduced insulin requirements independently by 71% (95% CrI: 52%, 84%) and 67% (95% CrI: 48%, 85%), respectively. Additionally, both the DASH (MD: −587.6; 95% CrI: −752.12, −421.85, low CoE) and low GI diets (MD: −180.09, 95% CrI: −267.48, −94.65, low CoE) reduced birth weight significantly, with the DASH diet also demonstrating effects in reducing macrosomia by 89% (95% CrI: 53%, 98%) and lowering the cesarean section rate by 46% (95% CI: 27%, 60%). However, exercise did not affect infant outcomes. Our findings suggest that the DASH diet and low GI diet and resistance exercise may be beneficial for maternal outcomes in pregnancies with GDM. The impact on infants is primarily observed through dietary interventions. Future research, characterized by higher quality and evidence grades, is necessary to complement and substantiate our findings.

This study was registered with PROSPERO as CRD 42024527587.

**Statement of Significance**

Currently, the evidence for lifestyle interventions in gestational diabetes mellitus is weak. The use of lifestyle interventions in clinical practice requires consideration of the specific situation of pregnant women. Future research, characterized by higher quality and evidence grades, is necessary to complement and substantiate our findings.

## Introduction

Gestational diabetes mellitus (GDM), characterized by glucose intolerance during pregnancy, has emerged as a global epidemic [1], with rapidly escalating prevalence in the last 10–20 y [[2], [3], [4]]. Of grave concern, GDM is associated with an increased risk of substantial adverse short-term and long-term maternal and fetal consequences [[5], [6], [7]]. Specifically, women with GDM exhibit higher rates of cesarean delivery, birth injury, preterm delivery, pre-eclampsia, and shoulder dystocia and are at elevated risk for developing type 2 diabetes mellitus and cardiovascular disease postpartum [[8], [9], [10]]. Moreover, their offspring face increased odds of macrosomia, neonatal hypoglycemia, hyperbilirubinemia, as well as obesity, impaired glucose tolerance, type 2 diabetes in childhood or early adulthood, and cardiovascular disease later in life [6,7,[9], [10], [11]]. This is because pregnancy is a critical period for fetal development, and any disturbance in maternal physiology can lead to altered disease susceptibility in the offspring throughout their life [12], thereby perpetuating a vicious intergenerational cycle of obesity and diabetes. Given the multitude of adverse outcomes, early identification and management of GDM is crucial to mitigate these risks for both mothers and their offspring.

During pregnancy, physiologic adaptations such as hormonal fluctuations and reorganization in the brain modulate taste perception and increase maternal cravings for specific foods, leading to subconscious alterations in dietary behaviors and eating patterns [12]. These changes can contribute to excessive weight gain and elevate risk of developing GDM. As a key component of lifestyle interventions, medical nutrition therapy (MNT) helps address these issues by providing adequate caloric intake to meet the nutritional demands of both the pregnant woman and fetus, achieve optimal glycemic control, and promote appropriate gestational weight gain [13]. Similarly, physical activity has been commonly prescribed for individuals with diabetes due to its beneficial effects on glycaemia and insulin sensitivity [14], effectively reducing GDM risk [15,16]. Therefore, lifestyle interventions, particularly MNT and physical exercise, play a vital role in GDM management, with lifestyle modifications alone enabling over 70% of women diagnosed with GDM to achieve glycemic targets [13].

However, despite the pivotal role of MNT and physical activity, there are inconsistencies in the optimal dietary regimens or exercise modalities capable of achieving maternal euglycemia while concurrently improving perinatal outcomes [13,17]. Several recent systematic reviews have examined the impact of dietary approaches on maternal and neonatal outcomes [[18], [19], [20], [21]]. Viana et al. [19] and Wei et al. [20] reported a connection between low glycemic index (GI) diets and reduced risk of macrosomia, while Yamamoto et al. [18] and Han et al. [21] found no similar associations. Furthermore, although conflicting findings regarding the effects of low GI diets on neonatal birth weight were presented by studies [18,19,21], Yamamoto et al. [18] and Han et al. [21] observed improved glycemic control, enhanced insulin sensitivity, and lower mean birth weight with the dietary approaches to stop hypertension (DASH) diets. These results suggest heterogeneity in the impact of different dietary strategies on maternal and infant outcomes. In this context, assessing the comparative effectiveness of dietary interventions based solely on individual randomized controlled trials (RCTs) or even pair-wise meta-analyses is challenging. These studies are typically designed to evaluate 1 or more dietary interventions against a control group, rather than directly comparing different dietary approaches, which makes it difficult to draw definitive conclusions. Regarding physical activity, while several systematic reviews have demonstrated its beneficial effects on preventing GDM development [15,22,23], a recent Cochrane systematic review [24] highlighted the limited intervention studies especially among women with existing GDM, precluding definitive conclusions about differences between groups. These gaps underscore the need to evaluate and compare the relative effectiveness of various lifestyle intervention approaches.

Therefore, to address this crucial knowledge gap, we conducted a network meta-analysis (NMA) to comprehensively evaluate the intervention effects of dietary, exercise, or combined diet and/or exercise on glycemic control and maternal–infant outcomes among women with GDM, against each other or usual care.

## Research Design and Methods

We performed a systematic review and NMA following our previously published protocol (PROSPERO CRD42024527587). Our NMA was conducted following the PRISMA extension statement for reporting systematic reviews incorporating NMA [25] (Supplementary S2).

### Data sources and search strategy

The electronic databases PubMed, EMBASE, Web of Science, and Cochrane Library were systematically searched from inception through 10 March, 2024, to identify published RCTs evaluating the effectiveness of dietary approaches and/or exercise interventions in pregnant women with GDM. Eligible trials compared 1 or more types of diets and/or exercises with ≥1 of the following: another dietary approach, exercise intervention, control diet, usual diet, or usual care. The detailed search strategy is provided in Supplementary S3. Only English-language publications were included. Reference lists were manually screened to obtain additional relevant data. Reference management was performed using EndNote software. For inclusion and exclusion criteria, please see Supplementary S4. The definition of GDM, macrosomia, or preterm birth was based on each trial.

Literature screening was independently conducted by 2 reviewers (LZ and FW), with reasons for exclusion at the full-text review stage recorded. Discrepancies were resolved through discussion and, if needed, by consulting a third reviewer (PJL or ST).

### Risk of bias assessment

The risk of bias assessment was independently performed by 2 researchers (LZ and PJL) using the Cochrane Collaboration Tool [26]. Any disagreements that occurred during the assessment process were resolved through discussion between the 2 researchers. In cases where a consensus could not be reached, a third researcher (FW or ST) was consulted to provide a final judgment. The Cochrane Collaboration Tool evaluates the risk of bias in studies across several domains, including random sequence generation, blinding, incomplete data, and selective reporting. Each domain is assigned a label of high risk, low risk, or unclear risk to indicate the level of bias present in the study (Supplementary S5).

### Data extraction

Data of each included trial were collected using a predesigned data extraction form (Supplementary S6). Extracted data elements included the following: *1*) name of the ﬁrst author, year of publication, and country of study; *2*) participant characteristics, including the total sample size, age and BMI at baseline, and diagnostic criteria for GDM; *3*) study design and duration of intervention; *4*) dietary characteristics (including type, energy, macronutrient composition, ratio, and speciﬁc forms of dietary control) or the characteristics of exercise; and *5*) maternal and neonatal outcome measurements.

### Data synthesis and meta-analysis

The mean differences (MDs) or relative risks (RRs), along with SD of unified units was used for estimating postinterference effects. The conversion of SEM to SD is done using Formula A, while the overall pooled SD is determined using Formula B; n is the sample size. SD1 and SD2 represent the SDs of baseline and final measurements for each group, respectively, where *R* is the correlation coefficient between baseline and final measurements. If the exact value of *R* was not provided in the text, we used *R* = 0.5 as a conservative estimate.

$$\text{Formula}\;A:\text{SD}=SEM\times \sqrt{n}$$

$$\text{Formula}\;B:{SD}_{pooled}=\sqrt{{SD}_{1}^{2}+{SD}_{2}^{2}-2R\times {SD}_{1}\times {SD}_{2}}$$

If there are 2 similar or identical interventions within the same study, we adopted Formulas C–E as a merged approach for analysis. Formulas C–E is as follows, where *n* is the sample size, subscript number is the intervention, and mean is the mean value of each intervention:

$$\text{Formula}\;C:N_{\text{Merged}}=n_{1}+n_{2}$$

$$\text{Formula}\;D:{\text{Mean}}_{\text{merged}}=\frac{{Mean}_{1}\times n_{1}+{Mean}_{2}\times n_{2}}{n_{1}+n_{2}}$$

$$\text{Formula}\;E:{\text{SD}}_{\text{merged}}=\sqrt{\frac{\left(n_{1}-1\right)\times {SD}_{1}^{2}+\left(n_{2}-1\right)\times {SD}_{2}^{2}+\frac{n_{1}\times n_{2}}{n_{1+}n_{2}}\times {\left({Mean}_{1}-{Mean}_{2}\right)}^{2}}{n_{1}+n_{2}-1}}$$

We also merged the blood glucose concentrations measured 2 h after breakfast, lunch, and dinner using weighted averaging and included them in the network analysis. Since the number of individuals measured at 2 h after breakfast, lunch, and dinner were the same, we used the following merging formula (Formula F and Formula G): *i* = 1 signifies the starting point of summation; *i* is a variable that takes on different values during the summation process; *n* represents the end point of summation. ${Mean}_{merged}$ denotes the overall mean; ${Mean}_{i}$ represents the mean of the *i*th group:

$$\text{Formula}\;F:{\text{Mean}}_{\text{merged}}=\frac{\sum_{i=1}^{n}\left(n_{i}\times {Mean}_{i}\right)}{\sum_{i=1}^{n}n_{i}}$$

$$\text{Formula}\;G:{\text{SD}}_{\text{merged}\;}=\sqrt{\frac{\sum_{i=1}^{n}{n_{i}[SD}_{i}^{2}+{\left({Mean}_{merged}-{Mean}_{i}\right)}^{2}]}{\sum_{i=1}^{n}n_{i}}}$$

For dichotomous data, all our RR estimates and 95% credible intervals (CrI) are directly derived from the posterior distribution of the Bayesian model. The Bayesian approach addresses zero-event issues by specifying appropriate previous distributions, eliminating the need for additional data modification or correct when zero events occur in some treatment arms [27].

After a thorough review, we extracted data on fasting plasma glucose (FPG), 2-h postprandial glucose (2hPG), birth weight, macrosomia, insulin requirements, and preterm birth for NMA. However, for outcomes such as HOMA-IR, glycated hemoglobin A1c (HbA1c%), large for gestational age (LGA), and cesarean section rate (CSR), we performed pair-wise meta-analyses due to insufficient interconnectivity among these outcomes to form a comprehensive network.

The *I*^2^ statistic was used to assess the heterogeneity among the included studies. An *I*^2^ value ranging from 0% to 50% was considered moderate heterogeneity, while values exceeding 50% but <75% were deemed to represent high heterogeneity. Very high heterogeneity was denoted by an *I*^2^ value of >75%. The selection of the appropriate model for NMAs was based on both the degree of heterogeneity and a comparison of the Deviance Information Criterion (DIC). In case of moderate heterogeneity, a fixed-effects model may be applicable, while a random-effects model may be preferred in the presence of high or extremely high heterogeneity. Additionally, a difference in DIC of >3 between the 2 models was deemed significant, supporting the use of the model with the lower DIC value for the subsequent analysis (Supplementary S7).

The network analysis was conducted using a Bayesian framework, using the Markov chain Monte Carlo (MCMC) simulation technique [28]. To achieve model convergence, 50,000 simulation iterations and 10,000 adaptation iterations were performed, using 4 chains with a thinning interval of 10. Convergence was assessed by examining trace plots, density plots, and Gelman–Rubin–Brooks figures.

The node-splitting method was used to evaluate local inconsistency within the network, while DIC was used to assess the global inconsistency of the network. DIC differences >3 between the consistency and inconsistency models is considered as evidence of global inconsistency (Supplementary S8). We used a qualitative approach to assess transitivity and tested for potential systematic differences that may violate the principle of transitivity through meta-regression. The ranking probabilities of the interventions were presented using surface under the cumulative ranking (SUCRA) values, which provide a summary statistic of the overall ranking of each outcome (Supplementary S10 and Supplementary S9). All statistical analyses were performed using the gemtc package in R (version 4.3.1) and Stata (version 14) software. Data were evaluated using 95% CrI, and a *P* value of <0.05 was statistically significant.

The quality of evidence was assessed using the Grading of Recommendations, Assessment, Development, and Evaluation (GRADE) approach, following the recommendation by the GRADE Working Group [[29], [30], [31]]. Initially, all included RCTs were assigned a score of 4, representing high-quality evidence. Subsequently, the score was downgraded based on the presence of limitations, indirectness, inconsistency, imprecision, and publication bias. The final quality of evidence was categorized into 4 levels: high, moderate, low, and very low. The GRADE level for each direct and indirect comparison was visually represented using a color-coding scheme in league table (Supplementary S11).

We conducted sensitivity analyses to assess the robustness of our findings. Studies were iteratively removed in arms based on high heterogeneity or zero-event occurrence (leave-1-arm-out approach), and the potential impact on network geometry, effect estimates, between-study heterogeneity (*I*^2^ statistic), and ranking probabilities were evaluated (Supplementary S14 and Supplementary S12-S13). If network disconnection occurred after study removal, the remaining evidence was analyzed separately for each subnetwork, provided the subnetworks still satisfied the coherence assumption of NMA. For subnetworks that could not be validly analyzed due to limited evidence, we further excluded those data. To address studies with zero events, we performed sensitivity analyses by excluding them and examining changes in network structure and effect estimates. The criteria for determining result instability were prespecified as follows: *1*) a change in network geometry, *2*) a significant shift in effect estimates, and *3*) a notable alteration in ranking probabilities.

## Results

**FIGURE 1:**
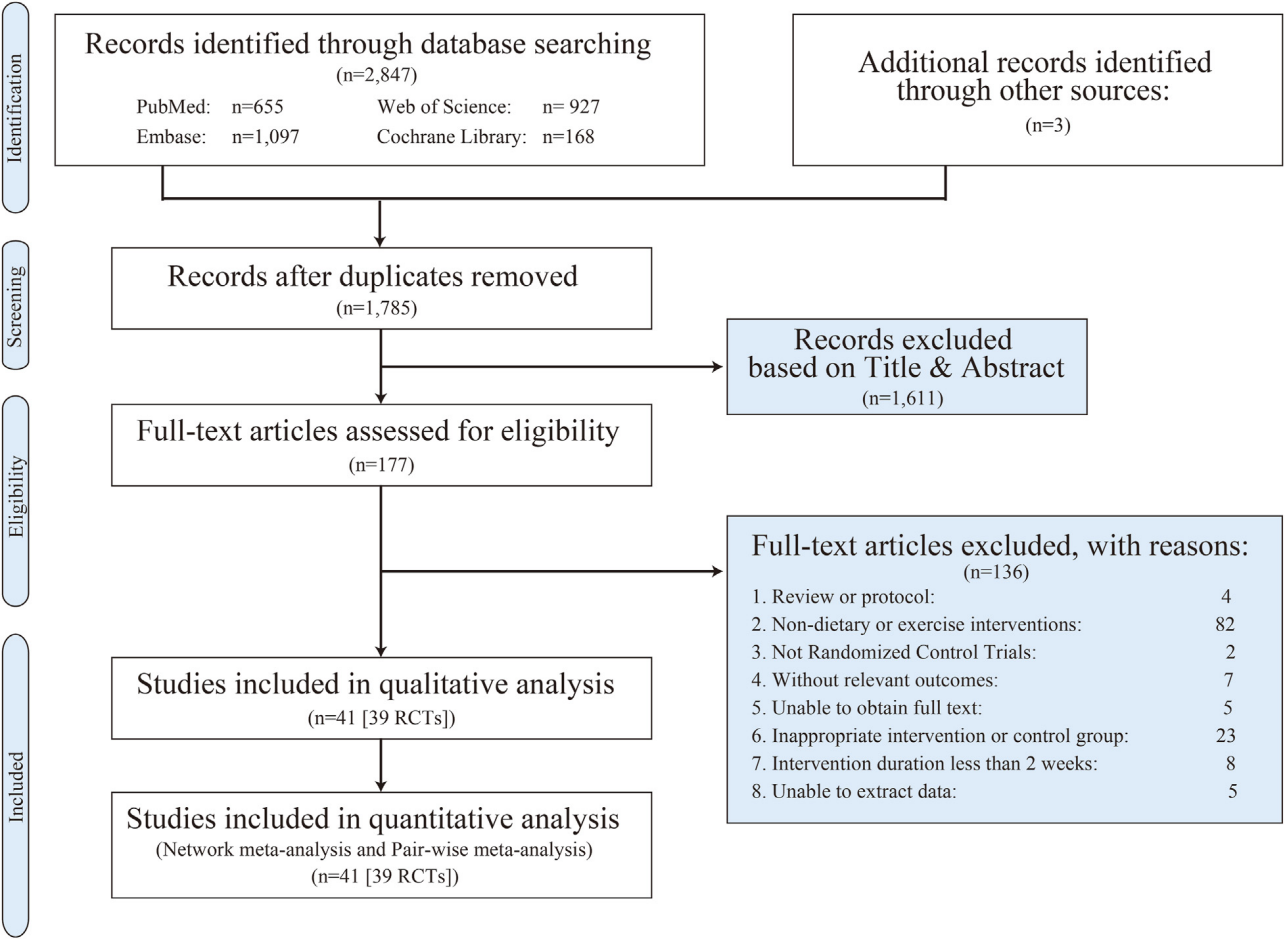
PRISMA flow diagram. During the literature selection process, we retrieved 2850 articles from 4 databases that aligned with our initial search strategy. After screening, we excluded 2809 articles that did not meet the inclusion criteria. Ultimately, 41 studies from 39 randomized controlled trials (RCTs) were included in our quantitative and qualitative analysis.

Among 2850 records screened, 177 full-text articles were further assessed for eligibility, and finally, 41 articles representing 39 RCTs reporting on 2712 women were included finally (Figure 1). The 39 RCTs included were conducted in 15 countries between 1989 and 2024.

### Characteristics of included studies

We summarized all characteristics of the included studies in Supplementary S6. In summary, the included RCTs were conducted across 15 countries and regions. China contributed the highest number of RCTs [[32], [33], [34], [35], [36], [37], [38], [39], [40], [41]], followed by the United States [[42], [43], [44], [45], [46], [47]] and Australia [[48], [49], [50], [51], [52], [53], [54]] with 6 RCTs each. Iran contributed 3 RCTs [[55], [56], [57], [58]], while Canada [59,60], Denmark [61,62], and Thailand [63,64] had 2 RCTs apiece. The remaining trials were from among Egypt [65], Poland [66], Brazil [67], Croatia [68], Turkey [69], Spain [70], Mexico [71], and India [72].

Regarding the dietary interventions, low GI/glycemic load diets were the most common, investigated in 8 RCTs [32,33,51,53,60,63,64,71]. Higher fat and lower carbohydrate (HFLC) diets followed with 6 RCTs [36,[43], [44], [45], [46],^59^]. Four RCTs each examined high complex carbohydrate (HCC) diets [43,44,46,72] and low carbohydrate (LC) diets [47,52,66,70], while 4 RCTs compared the DASH diet [40,[55], [56], [57]]. High carbohydrate [61,66], high GI/glycemic load [53,63], and soy-based diets [58,72] had 2 RCTs each. Single trial observed high monounsaturated fatty acids [61], high fiber (HF) [51], and energy-restricted diets [54].

For exercise interventions, aerobic exercise (AE) was the most frequently studied, appearing in 8 RCTs [35,38,39,42,45,[48], [49], [50],^65^]. Five RCTs [34,37,62,68,69] used structured exercise (SE) regimens, while resistance exercise (RE) was a component in 6 RCTs [35,38,41,59,65,67]. Only 1 RCT incorporated postmeal walking [48] or yoga [64].

### Risk of bias and CoE assessment

Supplementary S5 present the results of the risk of bias assessment. The results showed that the overall risk of bias in the included studies was high, with 90.2% of the included studies having a high risk of bias due to a lack of participant blinding. Considering that our research focused on diet and exercise interventions, it is relatively difficult to blind participants to these types of interventions. Other high risks of bias were mainly found in incomplete outcome data (4.9%) and other sources of bias (7.3%). Additionally, only 7.3% of the studies exhibited a low risk of bias. Moreover, our funnel plot exhibits a symmetric pattern, indicating no evidence of publication bias (Supplementary S15).

**FIGURE 2:**
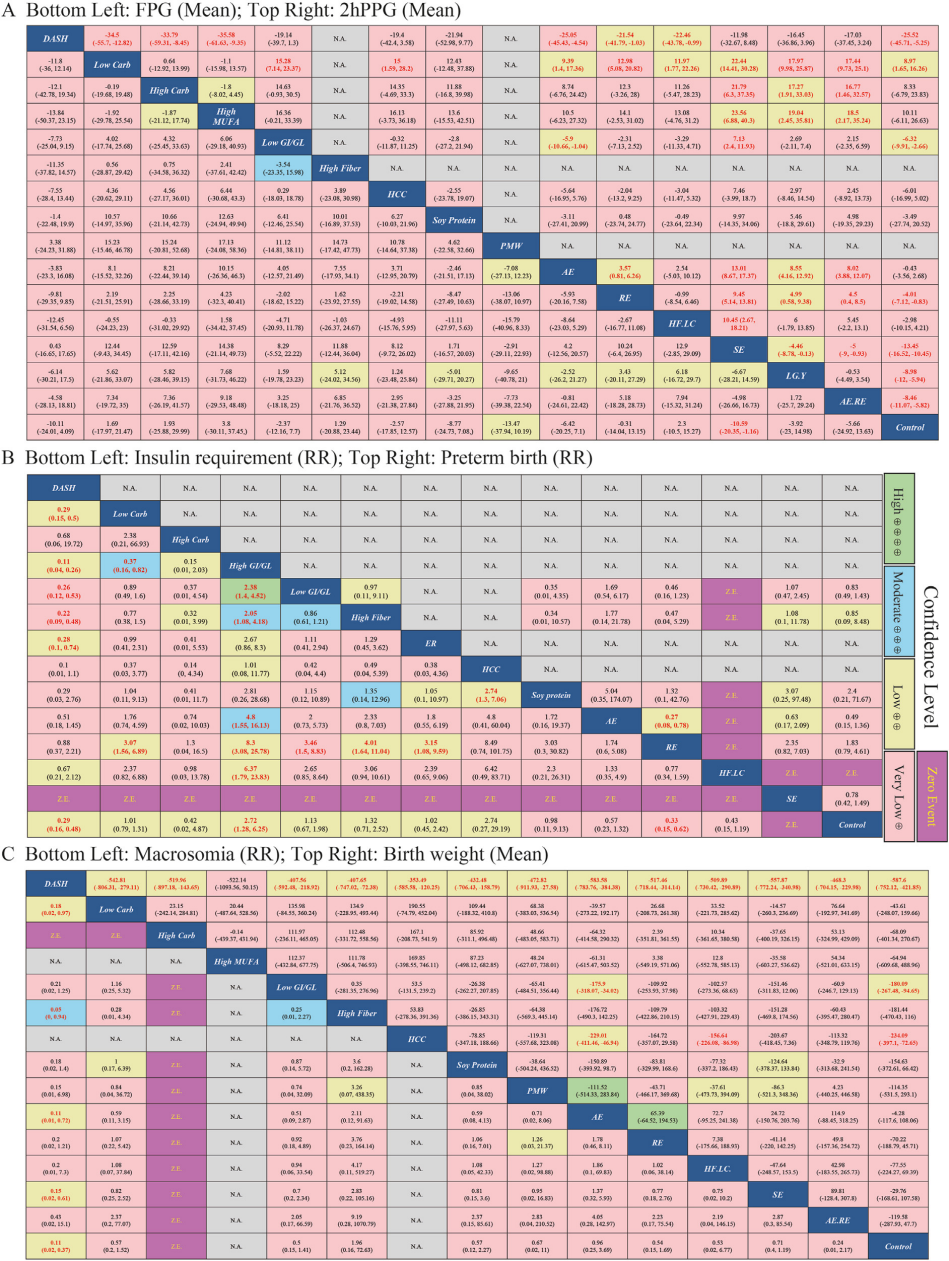
League table for maternal and infant outcomes. (A) The mean difference and 95% credible interval (CrI) for fasting plasma glucose (bottom left) and 2-h postprandial glucose (top right). (B) The relative risk (RR) and 95% CrI for insulin requirements (bottom left) and preterm birth (top right). (C) The RR and 95% CrI for macrosomia (bottom left), and the mean difference and 95% CrI for birth weight (top right). Different colors represent varying levels of certainty of evidence (CoE): green for high, blue for moderate, yellow for low, and pink for very low CoE. Purple indicates zero events, while gray denotes missing data. The red numbers represent statistical significance. All data are interpreted from left to right, and less is better for all measures. For mean, negative values indicate that the interference on the left is better than the interference on the right, while positive values indicate that the interference on the left is worse than the interference on the right. For RR, values <1 indicate that the interference on the left is better than the interference on the right, while values >1 indicate that the interference on the left is worse than the interference on the right. 2hPPG, 2-h postprandial glucose; AE, aerobic exercise; AE.RE, aerobic exercise+resistance exercise; Carb, carbohydrates DASH, dietary approaches to stop hypertension; ER, energy restriction; FPG, fasting plasma glucose; GI/GL, glycemic index/glycemic load; HCC, high complex carbohydrate; HF.LC, higher fat/lower carbohydrates; LG.Y, low GI+yoga; MUFA, monounsaturated fatty acid; N.A., no available data; PMW, postmeal walking; RE, resistance exercise; SE, structured exercise; Z.E., zero event.

We also evaluated the quality of evidence (GRADE) for the included studies to optimize the interpretation of our results (Supplementary S11). The certainty of evidence (CoE) was categorized into 4 levels: high, moderate, low, and very low, and all results are presented in Figure 2 using different colors. Overall, the included studies in our research demonstrated a very low CoE. Comparisons with low CoE were scattered throughout the network, while moderate and high CoEs were mainly observed in indirect comparisons.

### Convergence assessment

To assess the goodness of fit in our Bayesian NMA model, we used the MCMC method to sample and estimate the posterior distribution. This was done to observe whether the posterior distribution converged to a stable state during the MCMC process, thereby ensuring the reliability of the estimates we obtained.

Our results are presented in Supplementary S16 and S17. Through visual inspection, we did not observe any fluctuations of the individual chains in the trace-density plots, indicating that the overlapping area formed by the fluctuation range of most chains exhibited stable convergence characteristics. Additionally, almost all 4 chains converged to an approximately normal distribution, and the bandwidth values tended to be stable (close to 0) (Supplementary S16). Our Brooks–Gelman–Rubin diagnosis plot showed that the median and 97.5% shrink factors converged toward 1 (Supplementary S17), and all the potential scale reduction factor values were equal to 1 (potential scale reduction factor of <1.05 is considered a good fit for the Markov chain). These results indicate a satisfactory fit for our established NMA model.

### Outcome measures

We performed NMA for 6 maternal (FPG, 2hPG, and insulin requirements) and neonatal (birth weight, macrosomia, and preterm birth) outcomes. Three outcomes (insulin requirements, macrosomia, and preterm birth) encountered the issue of zero events. Although we included the zero-event populations and computed effect estimates using Bayesian models, the extreme values obtained may introduce substantial uncertainty in the results. To avoid potential overestimation bias, we temporarily excluded the zero-event arms from the league tables (Figure 2B, C, bottom left). Additionally, we performed pair-wise meta-analyses for outcome measures that were not suitable for NMA, including HOMA-IR, HbA1c%, LGA, and CSR (Supplementary S18).

#### Maternal outcomes

**FIGURE 3:**
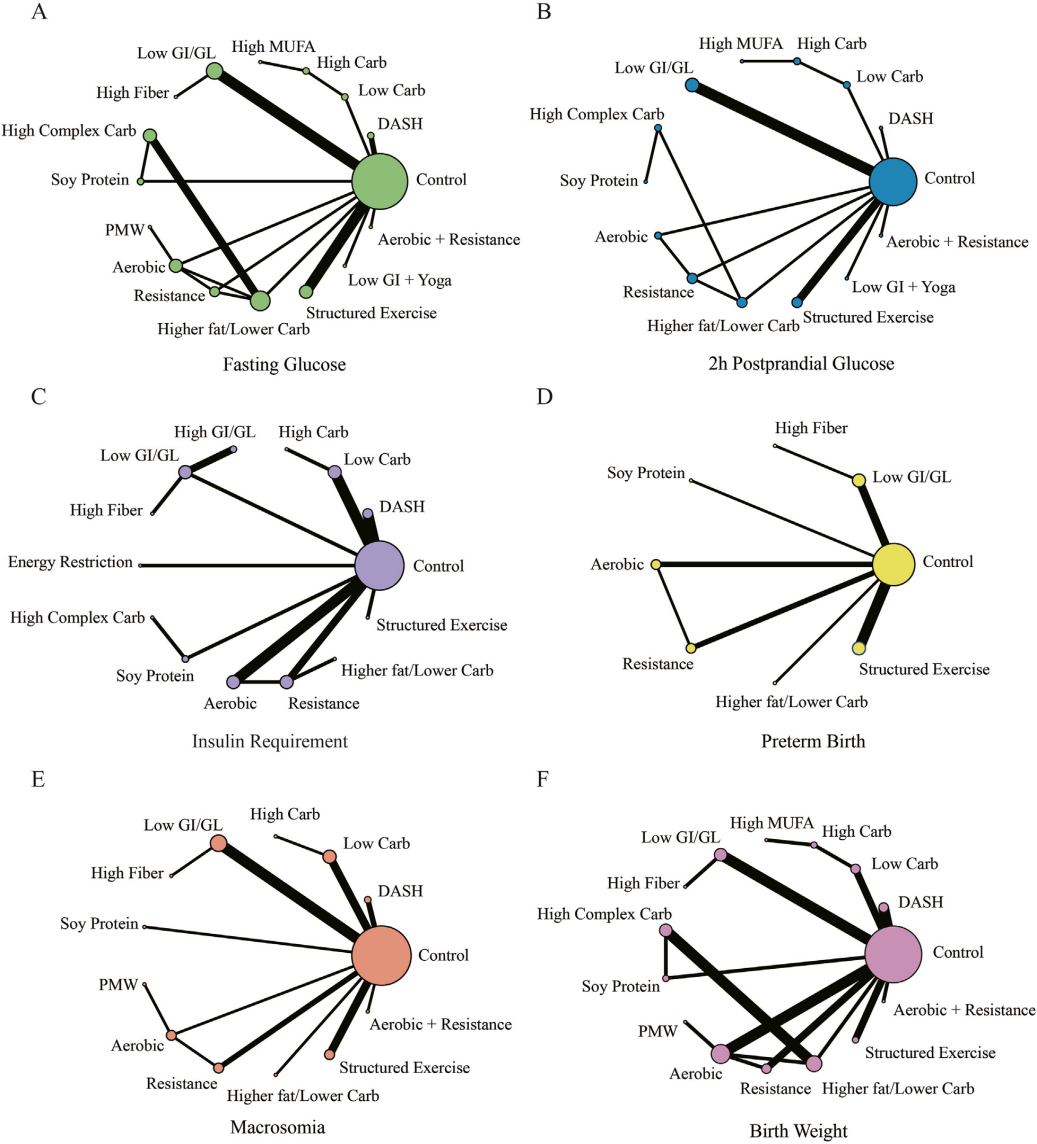
Network plots for maternal and infant outcomes. (A) Network comparisons of different treatments regarding the outcome of fasting plasma glucose. Network plots for the outcome of 2-h postprandial glucose levels (B), for insulin requirements (C), and for the outcome of preterm birth (D). (E) Network plots depicting study comparisons for the outcome of macrosomia. (F) Comparisons between treatments in terms of birth weight. Network plots are composed of nodes and lines. The different nodes represent different studies, with the node size corresponding to the number of participants. The lines connecting the nodes indicate direct comparisons between studies, and the width of each line is proportional to the number of studies involved in that particular comparison. Aerobic, aerobic exercise; Carb, carbohydrates DASH, Dietary approaches to stop hypertension; GI/GL, glycemic index/glycemic load; MUFA, monounsaturated fatty acid; PMW, postmeal walking; Resistance, resistance exercise.

In this analysis, 3 indices related to maternal glycemic parameters (FPG, 2hPG, and insulin requirement) were subjected to NMA. FPG was examined in 28 studies, which collectively involved 1883 participants. Moreover, 20 studies, with a combined sample size of 1561 participants, assessed 2hPG. Finally, insulin requirements were investigated in 22 studies, encompassing 1511 participants. To visualize the complex relationships among studies for each outcome, network plots were constructed, as presented in Figure 3A–C, respectively.

**FIGURE 4:**
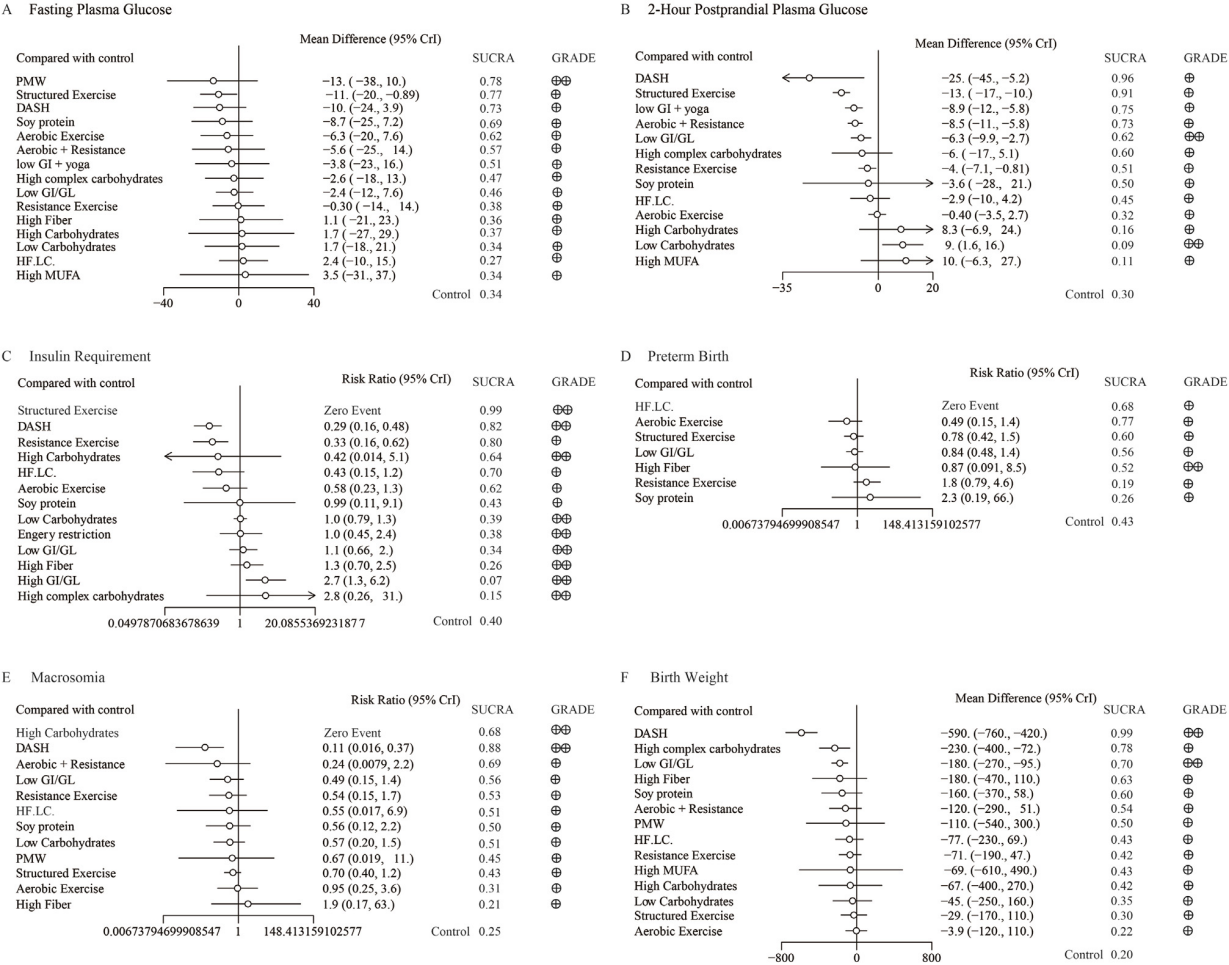
Forest plot comparison with the control group, SUCRA, and GRADE rating. The forest plots present network effect estimates of the direct and indirect evidence relative to the control group. (A) Fasting plasma glucose; (B) 2-h postprandial glucose; (C) insulin requirements; (D) preterm birth; (E) macrosomia; and (F) birth weight. The effect estimates are presented using means, relative risks, and 95% credible intervals. ⊕, very low certainty; ⊕⊕, low certainty. Aerobic, aerobic exercise; Carb, carbohydrates DASH, dietary approaches to stop hypertension; GI/GL, glycemic index/glycemic load; GRADE, Grading of Recommendations Assessment, Development and Evaluation; MUFA, monounsaturated fatty acid; PMW, postmeal walking; Resistance, resistance exercise; SUCRA, surface under the cumulative ranking curve.

The NMA for FPG encompassed 19 direct comparisons, with the results depicted in Figures 2A (bottom left) and 3A. When compared with the control, only SE demonstrated a significant difference (MD: −10.59; 95% CrI: −20.35, −1.16; very low CoE). No evidence of global or local inconsistency was observed in these results (Supplementary S19 and Supplementary S8). However, while no substantial global heterogeneity was noted (Supplementary S8), local heterogeneity was significant in 8 comparisons (Supplementary S20). The postmeal walking exhibited the highest SUCRA value (0.78), followed by the SE (0/77), and the lowest was the HFLC diet (0.27). Comprehensive rank probabilities and SUCRA values for all interventions are provided in Figure 4 and Supplementary S9.

In the NMA of 2hPG, 15 direct interventions were compared, the results of which are shown in Figures 2A (top right) and 3B. Multiple comparisons yielded significant outcomes, displayed in Figure 2A (top right). Compared with the control, the DASH diet showed the most effective approach (MD: −25.52; 95% CrI: −45.71, −5.25, very low CoE). Moreover, SE (MD: −13.45; 95% CrI: −16.52, −10.45, very low CoE), low GI plus yoga (MD: −8.98; 95% CrI: −12, −5.94, very low CoE), and AE plus RE (MD: −8.46; 95% CrI: −11.07, −5.82, very low CoE) demonstrated significant reductions in 2hPG and ranked higher than other interventions (Figure 4B and Supplementary S9). However, LC significantly increased 2hPG (MD: 8.97; 95% CrI: 1.65, 16.26, low CoE), exhibiting the worst approach in this outcome measure (Figure 4B and Supplementary S9). Similar to FPG, no evidence of global heterogeneity or inconsistency was found in these results (Supplementary S8). However, significant local inconsistencies emerged between HFLC and RE (*P* = 0.04), and 3 comparisons (HFLC and control; SE and control; HFLC and RE) also exhibited local heterogeneity (Supplementary S19 and S20).

Fourteen direct comparisons formed the network for insulin requirements, with the network diagram and analysis results shown in Figures 2B (bottom left) and 3C, respectively. The evidence quality of this network was relatively higher than the glucose measures, and multiple comparisons demonstrated significant differences (Figure 2B, bottom left). Due to the zero-event issue in SE, we were unable to effectively explain the relevant data. Therefore, although we included participants of SE in the network analysis, the data related to SE were excluded from the data interpretation. From the remaining results, DASH (RR: 0.29; 95% CrI: 0.16, 0.48, low CoE) and RE (RR: 0.33; 95% CrI: 0.15, 0.62, very low CoE) appear to perform better than the control group (Figure 2B, bottom left). Conversely, compared with the control group, only high GI showed poor performance (RR: 2.72; 95% CrI: 1.28, 6.25, low CoE). No inconsistency or heterogeneity was detected across the NMA (Supplementary S19 and Supplementary S8).

#### Neonatal outcomes

In this analysis, we included 3 outcomes, preterm birth, macrosomia, and birth weight, as measures of neonatal outcomes. For the preterm birth analysis, 1344 participants and 15 direct comparisons were included. See Figure 3D for the network plot and Figure 2B, top right, for the NMA results. Except for AE showing a better effect than RE (RR: 0.27; 95% CrI: 0.08, 0.78, low CoE), no treatment demonstrated superiority over the control group (Figure 2B, top right). The SUCRA ranking suggests that AE may be the most effective treatment for this outcome, while RE ranks the poorest. Although no significant inconsistency was observed (Supplementary S19), a comparison displayed local heterogeneity (low glycemic index (LGI) compared with control; *I*^2^ = 54.9%) (Supplementary S20).

For macrosomia, 22 direct comparisons and 1788 participants were included (Figure 3E). In this analysis, not only did DASH outperform the control group (RR: 0.11; 95% CrI: 0.02, 0.37, low CoE) but it also demonstrated superiority over HF (RR: 0.05; 95% CrI: 0, 0.94, moderate CoE), AE (RR: 0.11; 95% CrI: 0.01, 0.72, low CoE), and SE (RR: 0.15, 95% CrI: 0.02, 0.61, low CoE) (Figure 2C, bottom left). No significant differences were found among the other comparisons. DASH ranked first in the SUCRA, followed by AE+RE, with HF ranking the lowest (Figure 4 and Supplementary S9). There was no evidence of inconsistency (Supplementary S19 and Supplementary S8). Although we found no evidence of network heterogeneity (Supplementary S8), 1 comparison exhibited local heterogeneity (SE compared with control) (I^2^=74·2%) (Supplementary S20).

For the birth weight outcome, 28 direct comparisons and 1617 participants were included in the network comparison, with the network plot presented in Figure 3F. Low CoE suggests that compared with all other treatments except HMU, the DASH diet exhibited a better effect in reducing birth weight. Meanwhile, both the low GI diet (MD: −180.09; 95% CrI: −267.48, −94.65, low CoE) and the HCC diet (MD: −234.09; 95% CrI: −397.1, −72.65, very low CoE) demonstrated significant differences compared with the control group (Figure 2C, top right). The DASH diet ranked first in the SUCRA, followed by the HCC and low GI diets, while the control group ranked last for this outcome (Figure 4 and Supplementary S9). No significant global inconsistency or overall heterogeneity was detected across the network. However, another comparison (HFLC compared with control) exhibited significant local inconsistency (*P* = 0.045) and heterogeneity (*I*^2^ = 68.1%) (Supplementary S19 and S20). Additionally, local heterogeneity (*I*^2^ = 68.8%) was also present for the HFLC and AE comparison (Supplementary S20).

#### Pair-wise meta-analysis outcomes

We additionally extracted data on HOMA-IR, HbA1c%, LGA, and CSR. However, as these data were disconnected and could not constitute an analyzable network, we therefore synthesized them through pair-wise meta-analyses.

No intervention was found to significantly improve HOMA-IR, HbA1c%, or LGA measurements. However, as for CSR, the DASH diet was the sole intervention that demonstrated a reduced risk of cesarean section (RR: 0.54; 95% CrI: 0.40, 0.73; *I*^2^ = 0%) compared with the control group (Supplementary S18).

### Sensitivity analysis and regression analysis

Although no significant global heterogeneity and network inconsistency were evident (Supplementary S8), local heterogeneity and inconsistency were present across several outcome measures. To evaluate the robustness of our findings and explore potential sources of heterogeneity, sensitivity analyses were conducted. Our findings indicate that the blood glucose results demonstrated a degree of instability. Following the sensitivity analysis, the effect estimates and rankings underwent significant changes, while the other measurements remained relatively stable, and null events had no bearing on the outcomes. For the details of the sensitivity analysis, please refer to Supplementary S21.

Additionally, since the duration of the intervention (in weeks) was identified as a potential factor influencing all outcomes, we conducted a meta-regression analysis using duration as a covariate to investigate its effect on the estimates. Our results indicated that duration did not significantly impact the estimates (Supplementary S22), suggesting that it does not violate the transitivity assumption. Furthermore, no other factors that would violate the transitivity assumption were identified.

## Discussion

Lifestyle therapies are the primary treatment for gestational diabetes mellitus (GDM). To our knowledge, this is the first review to systematically integrate and compare the effects of lifestyle interventions on glycemic control and neonatal outcomes using an NMA.

This NMA included 2712 women with GDM from 41 studies (39 RCTs). Among the dietary approaches, our findings revealed that adherence to the DASH diets significantly improved maternal FPG and 2hPG concentrations, while as reducing the likelihood of insulin requirement. Additionally, a low GI diet reduced both 2hPG and birth weight. Certain dietary patterns, such as DASH and low GI diets, also had significant effects on fetal outcomes, whereas these effects were not observed with exercise interventions.

Regarding the glucose control, the latest published pair-wise meta-analysis revealed that compared with the control group, low GI diets resulted in greater reductions in fasting and postprandial blood glucose concentrations [18]. Although we did not observe the effect of a low GI diet on FPG control, we found a significant impact of a low GI diet on 2hPG. Intriguingly, we also discovered that the DASH diet significantly lowered both FPG and 2hPG concentrations in our study. This discrepancy may stem from Yamamoto et al. [18], excluding DASH studies in their sensitivity analysis. Supporting our findings, a recent meta-analysis suggested that adherence to the DASH diet by non-GDM pregnant women with cardiometabolic disorders significantly decreased FPG concentrations. It also reduced the incidence of pre-eclampsia, fetal macrosomia, large for gestational age, and newborn head circumference [73]. These data suggest that adherence to a DASH or low GI diet may be more effective for maternal glucose control compared with control diet or other dietary approaches. Additionally, our findings are further supported by the meta-analysis by Yamamoto et al., which showed that other dietary approaches, such as low carbohydrate, fat modification, and soy protein, did not significantly improve glucose control [18]. This effect may be attributed to the fact that both the DASH and low GI diets are rich in dietary fiber and low GI starch, which can slow the absorption of carbohydrates after meals, helping to prevent blood sugar spikes and postprandial glucose elevation [74]. Therefore, it is important to note that the DASH diet can be considered a low GI dietary approach [19].

Moreover, regarding insulin usage, the study by Viana et al. [19] demonstrated the advantage of a low GI diet in reducing insulin requirements, while Yamamoto et al. [18] found that a soy protein–rich diet could significantly decrease medication use. In contrast to these 2 studies, our research only indicated that the DASH diet could reduce the risk of insulin use by 71%. This divergence may be attributable to the varying number of studies included in the analyses, as we only incorporated 1 study [60] comparing low GI diets and control in the NMA of insulin use, and the scarcity of studies led to our underpowered conclusion. From a mechanistic perspective, low GI diets slow the rise in blood glucose concentrations [75], thereby reducing the burden on pancreatic β cells and improving insulin efficiency. Therefore, further high-quality studies are needed to confirm these questions.

Our findings on infant birth weight and macrosomia align with those by Viana et al. [19] that low GI diets can more meaningfully reduce birth weight compared with control but has no effect on decreasing the incidence of macrosomia. Remarkably, we found a significant advantage of DASH diet in reducing birth weight and the incidence of macrosomia. This is consistent with the findings by Li et al. [73], which showed that following the DASH diet during pregnancy decreased risk of macrosomia (RR: 0.294; 95% CI: 0.120, 0.721; *P* = 0.043) and large for gestational age (RR: 0.452; 95% CI: 0.211, 0.969; *P* = 0.041), as well as lowered mean newborn head circumference (weighted MD: −0.807; 95% CI: −1.283, −0.331; *P* = 0.001).

Our study did not identify any effect of dietary interventions on lowering the rate of preterm birth, which was also rarely reported in previous pair-wise meta-analyses for GDM [[18], [19], [20], [21]]. However, we found DASH diet also reduced the risk of cesarean section by 46%. In contrast, Li et al. [73] reported that following the DASH diet during pregnancy had no effect on cesarean section risk. This discrepancy is most likely due to differences in the studies included.

Exercise is a crucial lifestyle intervention. According to the American College of Obstetricians and Gynecologists recommendation [76], individuals with GDM are recommended to participate in moderate AE for a minimum of 30 min/d or 150 min/wk [76]. Concurrently, several studies have also demonstrated the beneficial effects of exercise on blood glucose control [77,78], and these benefits are associated with the physical activity level [79,80]. Consistent with these studies and recommendations, our research revealed that postmeal walking ranked first in FPG regulation. Structured exercise demonstrated significant improvements as well, ranking second. However, postmeal walking did not exhibit statistical significance, and SE exhibited substantial heterogeneity, rendering this conclusion unreliable and necessitating further large-scale studies to validate the benefits of these 2 interventions. Nonetheless, after excluding these studies, our sensitivity analyses indicated that combined AE+RE and low GI diets+yoga showed significant advantages in regulating FPG and 2hPG concentrations. Simultaneously, RE demonstrated more pronounced advantages in 2hPG regulation. AE is considered to significantly reduce blood glucose concentrations [76], and low GI has also been shown to have advantages in blood glucose control, insulin use, and birth weight [19,81]. Remarkably, RE has even been proven in a meta-analysis to significantly reduce FPG and 2hPG [82]. Although we did not find benefits of AE, we observed a significant effect of RE in reducing 2hPG. Encouragingly, we also discovered that combined aerobic with RE can further significantly reduce FPG and 2hPG glucose compared with RE alone, similar to the findings by Keating et al. [77]. On the contrary, although the effect size of low GI diets+yoga was lower than the that of the low GI group alone, there was no significant difference between the 2 groups, indicating that current evidence supports the superiority of combining low GI diets with yoga in blood glucose control but is insufficient to establish its superiority over using low GI diets alone. However, it should be also clarified that although our analysis yielded the abovementioned results, these findings are all based on a single trial. Therefore, we did not consider these positive results as the conclusion of our NMA. Larger-scale RCTs are needed in the future to confirm these findings and establish reliable conclusions.

Furthermore, in our study, RE reduced insulin requirements by 67%, which aligns well with the previous findings [82]. This suggests that RE can effectively decrease insulin resistance in GDM and enhance insulin sensitivity. While both AE and REs are recognized as effective interventions for reducing insulin resistance [83], our research did not identify any advantage of AE in lowering insulin requirements for GDM. Unfortunately, no studies have investigated whether combining AE and RE confers superior benefits in reducing insulin requirements. Future high-quality clinical trials are warranted to validate the efficacy of this combined intervention approach. Regarding infant outcomes, no significant effects of exercise were observed.

It is noteworthy that in our study, we also found that the LC diet significantly increased 2hPG concentration. However, since this conclusion was based on limited data from a single study, the conclusion should be interpreted with caution. Moreover, the high GI diet increased insulin use by 2.72-fold. This dietary approach warrants attention in future diabetes management.

Overall, our NMA reinforces the importance of nutritional interventions in improving glycemic control and infant outcomes in gestational diabetes. While the effects of dietary patterns on glucose concentrations may seem modest, even small improvements in glycemic balance can significantly reduce the need for medication [84]. This evidence highlights the potential of dietary management as an essential nonpharmacologic therapy to optimize maternal and neonatal outcomes. On the contrary, although exercise may also offer benefits, conclusions regarding its efficacy should be approached with caution due to the limited number of studies and high heterogeneity. Notably, RE has been found to reduce insulin requirements, suggesting its potential value in improving GDM care. These findings provide valuable insights that may inform future clinical practice.

### Limitation

A major limitation of our study is that several conclusions are drawn from evidence that is categorized as low to very low quality. This limitation arises mainly from the inherent difficulties in blinding exercise and nutritional interventions, which has led to a scarcity of high-quality RCTs in this domain, thus affecting the overall certainty of our findings. Despite this, we believe these results are still of value for publication and can serve as a useful reference. By systematically comparing the effectiveness of lifestyle interventions for individuals with GDM, our study provides valuable clinical insights and paves the way for future research in this field. Regarding the FPG outcome, there was substantial local heterogeneity, which may have arisen from different experimental designs and protocols, necessitating the exclusion of these studies and the performance of a new network analysis through sensitivity analyses, leading to significant deviations from our previous results. Since our conclusion for FPG was derived after excluding these studies, selection bias may have been introduced. Additionally, for 2hPG, after removing studies with high heterogeneity, our network was disconnected, requiring independent sensitivity analyses for the disjointed loops. Although this precluded direct comparisons of relative effect estimates between the disconnected loops, our results exhibited stability. Likewise, the conclusions for our other outcomes remained robust. However, it is crucial to acknowledge that the presence of single small trials, limited single-arm studies, inconsistent GDM diagnostic criteria, varying intervention durations, high risk of bias, and zero-event issues may restrict the generalizability and introduce uncertainty to our conclusions. In addition, due to the limited number of trials on each arm, we are unable to conduct subgroup analysis. Furthermore, our final conclusions do not account for high heterogeneity and interventions from single studies, such as structured training, which may lead to potential information loss and bias. Larger sample sizes and more high-quality RCTs are imperative for further confirmation in the future.

### Conclusion

This study highlighted the benefits of the DASH diet and low GI diet in glycemic control and infant birth weight outcomes for GDM, with the DASH diet additionally demonstrating advantages in reducing macrosomia and CSR. Furthermore, the DASH diet and RE reduced insulin requirements. Although we did not observe the advantages of AE alone, combining AE and RE demonstrated superior blood glucose regulation compared with either approach individually. However, it is important to note that these exercise-related conclusions are based on a single trial. Future investigations with larger sample sizes and high-quality RCTs are warranted to address the limitations of this study and reinforce our conclusions.

## Conflict of interest

The authors report no conflicts of interest.
