# Supplementary material for: Effects of Dietary Approaches and Exercise Interventions on Gestational Diabetes Mellitus: A Systematic Review and Bayesian Network Meta-analysis
Source: Adv Nutr. 2024 Oct 29;15(12):100330. doi: 10.1016/j.advnut.2024.100330 (PMC11629230; doi:10.1016/j.advnut.2024.100330)

**Effects of dietary approaches and exercise interventions on gestational diabetes mellitus: A systematic review and Bayesian network meta-analysis of randomized controlled trials**

Liang Zhang^1^, M.D., Ph.D., Fang Wang^2^, M.D., Syoichi Tashiro^1,3^, M.D., Ph.D., Pengju Liu^2^*, M.D.

^1^ Department of Rehabilitation Medicine, Kyorin University Faculty of Medicine, Tokyo, Japan

^2^ Department of Clinical Nutrition, Department of Health Medicine, Peking Union Medical College Hospital, Chinese Academy of Medical Science and Peking Union Medical College, Beijing, PR China

^3^ Department of Rehabilitation Medicine, Keio University School of Medicine, Tokyo, Japan

***Correspondence:** Peng Ju Liu, Department of Clinical Nutrition, Peking Union Medical College Hospital, NO. 1 Shuaifuyuan, Dongcheng District, Beijing, China. Email: [lpjjia@126.com](mailto:lpjjia@126.com)

***CONTENTS***

*Supplementary S1: Abbreviation list and definition*

*3-4*

*Supplementary S2: PRISMA NMA Checklist.*

*5-9*

*Supplementary S3: The search strategy of the systematic review.*

*10-11*

*Supplementary S4: The PICO criteria used to identify studies to be included in the systematic review.*

*12*

*Supplementary S5: Risk of bias assessment for included randomized controlled clinical trials.*

*13-14*

*Supplementary S6: Characteristics of included studies*

*15-31*

*Supplementary S7: Model selection*

*32*

*Supplementary S8: Global inconsistency and heterogeneity assessment*

*33*

*Supplementary S9: Rank Probability and SUCRA Values*

*34-37*

*Supplementary S10: Cumulative Rank Curve*

*38-40*

*Supplementary S11: The Grading of Recommendations, Assessment, Development, and Evaluation (GRADE)*

*41-55*

*Supplementary S12: League Tables for Sensitive Analysis*

*56-64*

*Supplementary S13: SUCRA for Sensitive Analysis*

*65-66*

*Supplementary S14: Network Plot for Sensitive Analysis*

*67-70*

*Supplementary S15: Funnel plots*

*71-73*

*Supplementary S16:* *Trace-density plots for Bayesian models*

*74-79*

*Supplementary S17: Brooks-Gelman-Rubin Plots*

*80-82*

*Supplementary S18: Meta-analysis results of included data that cannot be used for network analysis.*

*83*

*Supplementary S19: Inconsistency analysis*

*84-87*

*Supplementary S20: Heterogeneity Analysis*

*88-93*

*Supplementary S21: Sensitivity analysis*

*94-101*

*Supplementary S22: Meta-regression analysis*

*102-107*

***Supplementary S1: Abbreviation list and definition***

| ***Abbreviations*** | **Full names** | ***Definitions*** |
| --- | --- | --- |
| ***ACOG*** | American College of Obstricians and Gynecologists |  |
| ***AE*** | Aerobic Exercise |  |
| ***AGW*** | Average gestational week at delivery |  |
| ***AR/ARE*** | Aerobic + Resistance Exercise |  |
| ***BMI*** | Body Mass Index |  |
| ***Carb.*** | Carbohydrates |  |
| ***CGM*** | Continuous glucose monitoring |  |
| ***CoE*** | Certainty of evidence |  |
| ***CON*** | Control |  |
| ***CrI*** | Credible intervals |  |
| ***CSR:*** | Caesarean section rate |  |
| ***DAS/DASH*** | Dietary Approaches to Stop Hypertension |  |
| ***DIC*** | Deviance Information Criterion |  |
| ***ER*** | Energy Restriction diet | ≦70% of recommended dietary intake |
| ***FPG*** | Fasting Plasma Glucose |  |
| ***GDM*** | Gestational diabetes mellitus |  |
| ***GI*** | Glycaemic index |  |
| ***GL*** | Glycemic load |  |
| ***GRADE*** | Grading of Recommendations, Assessment, Development, and Evaluation |  |
| ***HbA1c*** | Glycated Hemoglobin |  |
| ***HC*** | High Carbohydrates | Carbohydrate% ≧60% of total energy |
| ***HCC*** | High Complex Carbohydrates |  |
| ***HF*** | High Fiber |  |
| ***HFL/HFLC*** | Higher fat/Lower Carbohydrates | fat %≧30% and carbohydrate% ≦55% of total energy |
| ***HG/HGI*** | High GI/GL |  |
| ***HMU/High MUFA*** | High Monounsaturated Fatty Acid | Source was a hybrid sunflower oil with high oleic acid (80%) content |
| ***HOMA-IR*** | Homeostatic Model Assessment for Insulin Resistance |  |
| ***HR*** | Heart rate |  |
| ***LC*** | Low Carbohydrates | Carbohydrate% ≦45% of total energy |
| ***LG/LGI*** | Low GI/GL | High in fruits, vegetables, whole grains, low-fat dairy, low saturated fats, cholesterol, refined grains and sweets. |
| ***LGA*** | Large for gestational age |  |
| ***LGY*** | Low GI + Yoga |  |
| ***MCMC*** | Markov Chain Monte Carlo |  |
| ***MLC*** | Modestly lower carbohydrate diet |  |
| ***NFI*** | Need for insulin therapy after intervention |  |
| ***OGL*** | Oral glucose load |  |
| ***OGTT*** | Oral Glucose Tolerance Test |  |
| ***PI*** | Ponderal index |  |
| ***PMW*** | Post-meal Walking |  |
| ***PPG*** | Postprandial Glucose |  |
| ***Prot.*** | Protein |  |
| ***PSRF*** | Potential Scale Reduction Factor |  |
| ***RC*** | Routine care |  |
| ***RCTs*** | Randomized controlled trials |  |
| ***RE*** | Resistance Exercise |  |
| ***RR*** | Relative risk |  |
| ***SD*** | Standard Deviation |  |
| ***SE*** | Structured Exercise | Physical activities that are guided and supervised by professionals, conducted according to a specific plan and arrangement. |
| ***Soy*** | Soy Protein |  |
| ***SUCRA*** | Surface Under the Cumulative Ranking |  |

***Supplementary S2: PRISMA NMA Checklist***

| ***Section/Topic*** | ***Item #*** | ***Checklist Item*** | ***Page #*** |
| --- | --- | --- | --- |
| **TITLE** |  |  |  |
| *Title* | 1 | Identify the report as a systematic review *incorporating a network meta-analysis (or related form of meta-analysis).* | ***1*** |
| **ABSTRACT** |  |  |  |
| *Structured summary* | 2 | Provide a structured summary including, as applicable:  **Background:** main objectives  **Methods:** data sources; study eligibility criteria, participants, and interventions; study appraisal; and *synthesis methods, such as network meta-analysis.*  **Results:** number of studies and participants identified; summary estimates with corresponding confidence/credible intervals; *treatment rankings may also be discussed. Authors may choose to summarize pairwise comparisons against a chosen treatment included in their analyses for brevity.*  **Discussion/Conclusions:** limitations; conclusions and implications of findings.  **Other:** primary source of funding; systematic review registration number with registry name. | 5-6 |
| **INTRODUCTION** |  |  |  |
| *Rationale* | 3 | Describe the rationale for the review in the context of what is already known*, including mention of why a network meta-analysis has been conducted.* | ***7-9*** |
| *Objectives* | 4 | Provide an explicit statement of questions being addressed, with reference to participants, interventions, comparisons, outcomes, and study design (PICOS). | 7-9 |
| **METHODS** |  |  |  |
| *Protocol and registration* | 5 | Indicate whether a review protocol exists and if and where it can be accessed (e.g., Web address); and, if available, provide registration information, including registration number. | 9 |
| *Eligibility criteria* | 6 | Specify study characteristics (e.g., PICOS, length of follow-up) and report characteristics (e.g., years considered, language, publication status) used as criteria for eligibility, giving rationale. *Clearly describe eligible treatments included in the treatment network, and note whether any have been clustered or merged into the same node (with justification).* | Suppl. S4 |
| *Information sources* | 7 | Describe all information sources (e.g., databases with dates of coverage, contact with study authors to identify additional studies) in the search and date last searched. | 9-10 |

***(Continued)***

***PRISMA NMA Checklist (Continued)***

| ***Section/Topic*** | ***Item #*** | ***Checklist Item*** | ***Page #*** |
| --- | --- | --- | --- |
| *Search* | 8 | Present full electronic search strategy for at least one database, including any limits used, such that it could be repeated. | 9-10, suppl. S3 |
| *Study selection* | 9 | State the process for selecting studies (i.e., screening, eligibility, included in systematic review, and, if applicable, included in the meta-analysis). | 9-10 |
| *Data collection process* | 10 | Describe method of data extraction from reports (e.g., piloted forms, independently, in duplicate) and any processes for obtaining and confirming data from investigators. | 11 |
| *Data items* | 11 | List and define all variables for which data were sought (e.g., PICOS, funding sources) and any assumptions and simplifications made. | 13, suppl.S4 |
| ***Geometry of the network*** | **S1** | Describe methods used to explore the geometry of the treatment network under study and potential biases related to it. This should include how the evidence base has been graphically summarized for presentation, and what characteristics were compiled and used to describe the evidence base to readers. | ***Fig. 3*** |
| *Risk of bias within individual studies* | 12 | Describe methods used for assessing risk of bias of individual studies (including specification of whether this was done at the study or outcome level), and how this information is to be used in any data synthesis. | 10-11 |
| *Summary measures* | 13 | State the principal summary measures (e.g., risk ratio, difference in means). *Also describe the use of additional summary measures assessed, such as treatment rankings and surface under the cumulative ranking curve (SUCRA) values, as well as modified approaches used to present summary findings from meta-analyses.* | 11-15 |
| *Planned methods of analysis* | 14 | Describe the methods of handling data and combining results of studies for each network meta-analysis. This should include, but not be limited to:   - *Handling of multi-arm trials;* - *Selection of variance structure;* - *Selection of prior distributions in Bayesian analyses; and* - *Assessment of model fit.* | 11-15 |
| ***Assessment of Inconsistency*** | **S2** | Describe the statistical methods used to evaluate the agreement of direct and indirect evidence in the treatment network(s) studied. Describe efforts taken to address its presence when found. | 14 |

***(Continued)***

***PRISMA NMA Checklist (Continued)***

| ***Section/Topic*** | ***Item #*** | ***Checklist Item*** | ***Page #*** |
| --- | --- | --- | --- |
| *Risk of bias across studies* | 15 | Specify any assessment of risk of bias that may affect the cumulative evidence (e.g., publication bias, selective reporting within studies). | **10-11** |
| *Additional analyses* | 16 | Describe methods of additional analyses if done, indicating which were pre-specified. This may include, but not be limited to, the following:   - Sensitivity or subgroup analyses; - Meta-regression analyses; - *Alternative formulations of the treatment network; and* - *Use of alternative prior distributions for Bayesian analyses (if applicable).* | 13-15 |
| **RESULTS†** |  |  |  |
| *Study selection* | 17 | Give numbers of studies screened, assessed for eligibility, and included in the review, with reasons for exclusions at each stage, ideally with a flow diagram. | 15, Fig.1 |
| ***Presentation of network structure*** | **S3** | Provide a network graph of the included studies to enable visualization of the geometry of the treatment network. | ***Fig.3*** |
| ***Summary of network geometry*** | **S4** | Provide a brief overview of characteristics of the treatment network. This may include commentary on the abundance of trials and randomized patients for the different interventions and pairwise comparisons in the network, gaps of evidence in the treatment network, and potential biases reflected by the network structure. | ***15, Fig.3, Suppl. S6*** |
| *Study characteristics* | 18 | For each study, present characteristics for which data were extracted (e.g., study size, PICOS, follow-up period) and provide the citations. | 16 |
| *Risk of bias within studies* | 19 | Present data on risk of bias of each study and, if available, any outcome level assessment. | 17, suppl. S5 |
| *Results of individual studies* | 20 | For all outcomes considered (benefits or harms), present, for each study: 1) simple summary data for each intervention group, and 2) effect estimates and confidence intervals. *Modified approaches may be needed to deal with information from larger networks.* | 18-23, Fig.2 |

***(Continued)***

***PRISMA NMA Checklist (Continued)***

| ***Section/Topic*** | ***Item #*** | ***Checklist Item*** | ***Page #*** |
| --- | --- | --- | --- |
| *Synthesis of results* | 21 | Present results of each meta-analysis done, including confidence/credible intervals. *In larger networks, authors may focus on comparisons versus a particular comparator (e.g. placebo or standard care), with full findings presented in an appendix. League tables and forest plots may be considered to summarize pairwise comparisons.* If additional summary measures were explored (such as treatment rankings), these should also be presented. | ***18-23, suppl.S18,Fig.2,4*** |
| ***Exploration for inconsistency*** | **S5** | Describe results from investigations of inconsistency. This may include such information as measures of model fit to compare consistency and inconsistency models, *P* values from statistical tests, or summary of inconsistency estimates from different parts of the treatment network. | ***18-23*** |
| *Risk of bias across studies* | 22 | Present results of any assessment of risk of bias across studies for the evidence base being studied. | 17,suppl.S5 |
| *Results of additional analyses* | 23 | Give results of additional analyses, if done (e.g., sensitivity or subgroup analyses, meta-regression analyses*, alternative network geometries studied, alternative choice of prior distributions for Bayesian analyses,* and so forth). | ***17-18,23-24,suppl.S16-17,S19-20,S22*** |
| **DISCUSSION** |  |  |  |
| *Summary of evidence* | 24 | Summarize the main findings, including the strength of evidence for each main outcome; consider their relevance to key groups (e.g., healthcare providers, users, and policy-makers). | 24-29 |
| *Limitations* | 25 | Discuss limitations at study and outcome level (e.g., risk of bias), and at review level (e.g., incomplete retrieval of identified research, reporting bias). *Comment on the validity of the assumptions, such as transitivity and consistency. Comment on any concerns regarding network geometry (e.g., avoidance of certain comparisons).* | 29-30 |
| *Conclusions* | 26 | Provide a general interpretation of the results in the context of other evidence, and implications for future research. | 31 |

***(Continued)***

***PRISMA NMA Checklist (Continued)***

| ***Section/Topic*** | ***Item #*** | ***Checklist Item*** | ***Page #*** |
| --- | --- | --- | --- |
| **FUNDING** |  |  |  |
| *Funding* | 27 | Describe sources of funding for the systematic review and other support (e.g., supply of data); role of funders for the systematic review. This should also include information regarding whether funding has been received from manufacturers of treatments in the network and/or whether some of the authors are content experts with professional conflicts of interest that could affect use of treatments in the network. | ***31*** |

*PICOS = population, intervention, comparators, outcomes, study design.*

** Text in italics indicate S wording specific to reporting of network meta-analyses that has been added to guidance from the PRISMA statement.*

*† Authors may wish to plan for use of appendices to present all relevant information in full detail for items in this section.*

***Supplementary S3: Search strategy of the systematic review***

| ***Databases Searched*** | ***Search terms*** |
| --- | --- |
| ***Medline (Pubmed) (From 1946), Cochrane Library*** | **((((((((((((((((((((((((((((((((((((((((((exercise[MeSH Terms]) OR (resistance training[MeSH Terms])) OR (rehabilitation[MeSH Terms])) OR (exercise therapy[MeSH Terms])) OR (exercise movement techniques[MeSH Terms])) OR (muscle stretching exercises[MeSH Terms])) OR (physical therapy modalities[MeSH Terms])) OR (sports[MeSH Terms])) OR (physical activity[Title/Abstract])) OR (exercise[Title/Abstract])) OR (train*[Title/Abstract])) OR (rehabilit*[Title/Abstract])) OR (activit*[Title/Abstract])) OR (move*[Title/Abstract])) OR (aerobic[Title/Abstract])) OR (resist*[Title/Abstract])) OR (strength*[Title/Abstract])) OR (fitness[Title/Abstract])) OR (cycling[Title/Abstract])) OR (bicycle[Title/Abstract])) OR (arm ergometer[Title/Abstract])) OR (walking[Title/Abstract])) OR (jogging[Title/Abstract])) OR (swimming[Title/Abstract])) OR (treadmill[Title/Abstract])) OR (gymnastic*[Title/Abstract])) OR (yoga[Title/Abstract])) OR (stretch*[Title/Abstract])) OR (pilates[Title/Abstract])) OR (taiji[Title/Abstract])) OR (sport*[Title/Abstract])) OR (flexib*[Title/Abstract])) OR (mobil*[Title/Abstract])) OR (therapeutic exercise[Title/Abstract])) OR (physical therapy[Title/Abstract])) OR ((((((((Diet[MeSH Terms]) OR (Diet, healthy)) OR (Diet* patterns)) OR (Diet* therapy)) OR (Nutrition*)) OR (Food intake)) OR (Dietary intake)) OR (Food consumption))) OR ((((Mediterranean Diet[MeSH Terms]) OR (Diets, Mediterranean[Title/Abstract])) OR (Mediterranean Diets[Title/Abstract])) OR (Diet, Mediterranean[Title/Abstract]))) OR (((((((((Diet, Plant-Based[MeSH Terms]) OR (Diet, Plant Based[Title/Abstract])) OR (Diets, Plant-Based[Title/Abstract])) OR (Plant-Based Diets[Title/Abstract])) OR (Plant-Based Diet[Title/Abstract])) OR (Plant Based Diet[Title/Abstract])) OR (Plant-Based Nutrition[Title/Abstract])) OR (Nutrition, Plant-Based[Title/Abstract])) OR (Plant Based Nutrition[Title/Abstract]))) OR ((((((Dietary Approaches To Stop Hypertension[MeSH Terms]) OR (DASH Diet[Title/Abstract])) OR (DASH Diets[Title/Abstract])) OR (Diet, DASH[Title/Abstract])) OR (Diets, DASH[Title/Abstract])) OR (Dietary Approaches To Stop Hypertension Diet[Title/Abstract]))) OR ((((Glycemic index[MeSH Terms]) OR (Glycemic load[MeSH Terms])) OR (Glycemic ind*[Title/Abstract])) OR (Glycemic load[Title/Abstract]))) OR (((((((((((((((((Diet, Carbohydrate-Restricted[MeSH Terms]) OR (Diet, Carbohydrate Restricted[Title/Abstract])) OR (Diet, Low Carbohydrate[Title/Abstract])) OR (Carbohydrate Diet, Low[Title/Abstract])) OR (Carbohydrate Diets, Low[Title/Abstract])) OR (Diets, Low Carbohydrate[Title/Abstract])) OR (Low Carbohydrate Diets[Title/Abstract])) OR (Carbohydrate-Restricted Diet[Title/Abstract])) OR (Carbohydrate Restricted Diet[Title/Abstract])) OR (Carbohydrate-Restricted Diets[Title/Abstract])) OR (Diets, Carbohydrate-Restricted[Title/Abstract])) OR (Low-Carbohydrate Diet[Title/Abstract])) OR (Diet, Low-Carbohydrate[Title/Abstract])) OR (Diets, Low-Carbohydrate[Title/Abstract])) OR (Low Carbohydrate Diet[Title/Abstract])) OR (Low-Carbohydrate Diets[Title/Abstract])) OR (Calori* restriction[Title/Abstract]))) AND (((((((((randomized controlled trial[MeSH Terms]) OR (randomized controlled trial[Publication Type])) OR (controlled clinical trial[Publication Type])) OR (clinical trial[Publication Type])) OR (placebo[Title/Abstract])) OR (random*[Title/Abstract])) OR (trial[Title/Abstract]))) OR (groups[Title/Abstract]))) AND ((((((((((Gestational diabetes mellitus[MeSH Terms]) OR (GDM[Title/Abstract])) OR (gestational diabetes[Title/Abstract])) OR (diabetes, pregnancy induced[Title/Abstract])) OR (diabetes mellitus, gestational[Title/Abstract])) OR (pregnancy, glucose intolerance[Title/Abstract])) OR (pregnancy, hyperglycaemia[Title/Abstract])) OR (pregnancy glycaemic index[Title/Abstract])) OR (Pregnancy-Induced Diabetes[Title/Abstract])) OR (Diabetes, Gestational[Title/Abstract]))/filter: randomized controlled trial** |
| ***Embase***  ***(From 1947)*** | ('diabetes mellitus gravidarum' OR 'diabetes mellitus of pregnancy' OR 'diabetes of pregnancy' OR 'diabetes, gestational' OR 'diabetes, pregnancy' OR 'gestational diabetes mellitus' OR 'maternal gestational diabetes mellitus' OR 'pregnancy diabetes' OR 'pregnancy diabetes mellitus' OR 'pregnancy-induced diabetes' OR 'gestational diabetes') AND ('diet pattern' OR 'dietary patterns' OR 'dietary pattern' OR 'Diet' OR 'diet influence' OR 'diet regimen' OR 'dietary effect' OR 'dietary influence' OR 'dieting' OR 'Diet, healthy' OR 'Diet* patterns' OR 'Diet* therapy' OR 'Nutrition*' OR 'Food intake' OR 'Dietary intake' OR 'Food consumption' OR 'diet, plant-based' OR 'plant based diet' OR 'plant based dietary pattern' OR 'plant-based eating pattern' OR 'plantbased diet' OR 'plant-based diet' OR 'diet, Mediterranean' OR 'MedDiet' OR 'Mediterranean diet' OR 'DASH-style diet' OR 'dietary approach to stop hypertension' OR 'dietary approach to stop hypertension (DASH) style diet' OR 'dietary approach to stop hypertension diet' OR 'dietary approaches to stop hypertension' OR 'Dietary Approaches to Stop Hypertension (DASH)' OR 'dietary approaches to stop hypertension diet' OR 'dietary approaches to stop hypertension style diet' OR 'glycaemia index' OR 'glycaemic index' OR 'glycaemic value' OR 'glycemia index' OR 'glycemic value' OR 'glycemie index' OR 'glycemic index' OR 'glycemic load' OR 'carbohydrate restricted diet' OR 'carbohydrate-poor diet' OR 'carbohydrate-restricted diet' OR 'diet, carbohydrate-restricted' OR 'diet, low carbohydrate' OR 'low carb diet' OR 'low carbohydrate diet' OR 'calori* restriction' OR '**Exercise**' OR '**resistance training**' OR '**exercise therapy**' OR '**exercise movement techniques**' OR '**muscle stretching exercises**' OR '**physical therapy modalities**' OR '**physical activity**' OR '**train***' OR '**rehabilit***' OR '**activit***' OR '**move***' OR '**aerobic**' **OR** '**resist***' OR '**strength***' OR '**fitness**' OR '**cycling**' OR '**bicycle**' OR '**arm ergometer**' OR '**walking**' OR '**jogging**' OR '**swimming**' OR '**treadmill**' OR '**gymnastic***' OR '**yoga**' OR '**stretch***' OR '**pilates**' OR '**taiji**' OR '**sport***' OR '**flexib***' OR '**mobil***' OR '**therapeutic exercise**' OR '**physical therapy**') AND ('controlled trial, randomized' OR 'randomised controlled study' OR 'randomised controlled trial' OR 'randomized controlled study' OR 'trial, randomized controlled' OR 'randomized controlled trial') **/filter: randomized controlled trial** |
| ***Web of Science*** | ((((((((((TS=(randomized controlled trial)) OR TS=(controlled trial, randomized)) OR TS=(randomised controlled study)) OR TS=(randomised controlled trial)) OR TS=(randomized controlled study)) OR TS=(trial, randomized controlled')) OR TS=(placebo)) OR TS=(random*)) OR TS=(trial)) AND ((((((((((((((((((((((((((((((((((((((((((((((((((((((((((((((((((((((TS=(Diet)) OR TS=(Dietary pattern)) OR TS=(Diet* patterns)) OR TS=(Diet* therapy)) OR TS=(Nutrition*)) OR TS=(Diet, healthy)) OR TS=(Food intake)) OR TS=(Dietary intake)) OR TS=(Food consumption)) OR TS=(plant-based diet)) OR TS=(diet, plant-based)) OR TS=(plant based diet)) OR TS=(plant based dietary pattern)) OR TS=(plant-based eating pattern)) OR TS=(plantbased diet)) OR TS=(plant-based diet)) OR TS=(Mediterranean Diet)) OR TS=(Diets, Mediterranean)) OR TS=(Mediterranean Diets)) OR TS=(Dietary Approaches To Stop Hypertension)) OR TS=(DASH Diet)) OR TS=(DASH Diets)) OR TS=(Diet, DASH)) OR TS=(Diets, DASH)) OR TS=(Dietary Approaches To Stop Hypertension Diet)) OR TS=(Glycemic index)) OR TS=(Glycemic load)) OR TS=(Glycemic ind*)) OR TS=(carbohydrate restricted diet)) OR TS=(carbohydrate-poor diet)) OR TS=(carbohydrate-restricted diet)) OR TS=(diet, carbohydrate-restricted)) OR TS=(diet, low carbohydrate)) OR TS=(low carb diet)) OR TS=(low carbohydrate diet)) OR TS=(Calori* restriction)) OR TS=(exercise)) OR TS=(resistance training)) OR TS=(rehabilitation)) OR TS=(exercise therapy)) OR TS=(exercise movement techniques)) OR TS=(muscle stretching exercises)) OR TS=(physical therapy modalities)) OR TS=(sports)) OR TS=(physical activity)) OR TS=(train*)) OR TS=(rehabilit*)) OR TS=(activit*)) OR TS=(move*)) OR TS=(aerobic)) OR TS=(resist*)) OR TS=(strength*)) OR TS=(fitness)) OR TS=(cycling)) OR TS=(bicycle)) OR TS=(arm ergometer)) OR TS=(walking)) OR TS=(jogging)) OR TS=(swimming)) OR TS=(treadmill)) OR TS=(gymnastic*)) OR TS=(yoga)) OR TS=(stretch*)) OR TS=(pilates)) OR TS=(taiji)) OR TS=(sport*)) OR TS=(flexib*)) OR TS=(mobil*)) OR TS=(therapeutic exercise)) OR TS=(physical therapy))) AND (((((((((((TS=(Gestational diabetes mellitus)) OR TS=(GDM)) OR TS=(gestational diabetes)) OR TS=(diabetes, pregnancy induced)) OR TS=(diabetes mellitus, gestational)) OR TS=(pregnancy, glucose intolerance)) OR TS=(pregnancy, hyperglycaemia)) OR TS=(pregnancy glycaemic index)) OR TS=(Pregnancy-Induced Diabetes)) OR TS=(Diabetes, Gestational)) OR TS=(diabetes mellitus gravidarum)) |

***Supplementary S4: Summary of the PICTOS criteria used to identify studies for inclusion and exclusion***

| **Parameter** | **Description** |
| --- | --- |
| ***Population*** | Women with gestational diabetes mellitus (GDM) [any type] defined by **trials authors** |
| ***Intervention*** | Eligible interventions include RCTs of GDM using any definite **dietary pattern**, **exercise characteristics**, or a combination intervention of diet and exercise |
| ***Comparison*** | Usual care, placebo, control intervention or healthy education, |
| ***Outcome*** | **Primary outcomes:**   1. Maternal fasting glucose, 2-hour postprandial glucose (continuous data) 2. Birth weight of newborns (continuous data) 3. Macrosomia (dichotomous data) 4. Need for insulin therapy after intervention (dichotomous data) 5. Preterm birth   **Secondary outcomes:**   1. Maternal fasting insulin (continuous data) 2. Homeostatic model assessment of insulin resistance (HOMA-IR, continuous data) 3. HbA1c% (continuous data) 4. Caesarean section rate (dichotomous data or continuous data) 5. Large for gestational age (LGA) (dichotomous data) |
| ***Time*** | The duration of the study was not less than 2 weeks |
| ***Study design*** | Randomized controlled trial (parallel or cross-over designs) |
| ***Exclusion*** | 1) trials that included participants with type 1 or type 2 diabetes if data for women with GDM were not provided independently;  2) cross-sectional, cohort or case-control studies, reviews, meta-analyses, case reports, and animal or cell experiments;  3) characteristics of diet or exercise in the trials were unavailable;  4) trials without expected outcomes;  5) trials with intervention duration of less than 2 weeks;  6) trials comparing different intensities of the same exercise, or utilizing nutritional supplements such as vitamins, probiotics, prebiotics, synbiotics, or inositol, etc. |

***Supplementary S5: Risk of bias assessment for included randomized controlled clinical trials***

| ***The first author (publication year)*** | ***Random sequence generation*** | ***Allocation concealment*** | ***Blinding of participants and personnel*** | ***Blinding of outcome assessment*** | ***Incomplete outcome data*** | ***Selective reporting*** | ***Other sources of bias*** |
| --- | --- | --- | --- | --- | --- | --- | --- |
| ***Asemi (2013a & 2013b)*** (55;56) | Low | Unclear | High | Low | Low | Unclear | Unclear |
| ***Asemi (2014)*** (57) | Low | Unclear | High | Unclear | Low | Low | Unclear |
| ***Avery (1997)*** (42) | Low | Unclear | High | Unclear | Low | Unclear | Low |
| ***Awad (2019)*** (65) | Low | Low | High | Unclear | Low | Low | Low |
| ***Brankston (2004)*** (59) | Unclear | Low | High | Unclear | Low | Unclear | High |
| ***Christie (2024)*** (48) | Low | Unclear | High | Unclear | Low | Low | Unclear |
| ***Cypryk (2007)*** (66) | Unclear | Unclear | High | Unclear | Unclear | Unclear | Unclear |
| ***de Barros (2010)*** (67) | Low | Low | High | Unclear | Low | Low | Low |
| ***Grant (2011)*** (60) | Unclear | Low | High | Unclear | High | Unclear | Unclear |
| ***Halse (2014 & 2015)*** (49;50) | Low | Low | High | Unclear | Low | Unclear | Unclear |
| ***Hernandez (2016)*** (44) | Unclear | Unclear | High | Unclear | Low | Low | High |
| ***Hernandez (2023)*** (43) | Low | Low | High | Unclear | Low | Low | Low |
| ***Jamilian & Asemi (2015)*** (58) | Low | Unclear | High | Unclear | Low | Unclear | Low |
| ***Jin (2022)*** (34) | Low | Low | High | Low | Low | Low | Low |
| ***Jovanovic-Peterson (1989)*** (45) | Unclear | Unclear | High | Unclear | Low | Unclear | Unclear |
| ***Kokic (2017)*** (68) | Low | Unclear | High | Low | Low | Low | Low |
| ***Lauszus (2001)*** (61) | Unclear | Low | Unclear | Unclear | Low | Low | Unclear |
| ***Louie (2011)*** (51) | Low | Low | Low | Low | Low | Low | Low |
| ***Lv (2019)*** (32) | Unclear | Unclear | High | Unclear | Unclear | Low | High |
| ***Ma (2014)*** (33) | Low | Unclear | High | Low | Low | Unclear | Low |
| ***Menek (2024)*** (69) | Low | Unclear | High | Unclear | Low | Low | Unclear |
| ***Mijatovic (2020)*** (52) | Low | Low | High | Unclear | High | Low | Low |
| ***Moreno-Castilia (2013)*** (70) | Low | Unclear | High | Unclear | Low | Low | Low |
| ***Moses (2009)*** (53) | Low | Unclear | High | Unclear | Low | Low | Low |
| ***Perichart-Perera (2012)*** (71) | Low | Unclear | High | Low | Low | Unclear | Low |
| ***Rae (2000)*** (54) | Low | Low | Low | Low | Low | Unclear | Low |

***(Continued)***

***Supplementary S5: Risk of bias assessment for included randomized controlled clinical trials (Continued)***

| ***The first author (publication year)*** | ***Random sequence generation*** | ***Allocation concealment*** | ***Blinding of participants and personnel*** | ***Blinding of outcome assessment*** | ***Incomplete outcome data*** | ***Selective reporting*** | ***Other sources of bias*** |
| --- | --- | --- | --- | --- | --- | --- | --- |
| ***Sanpawithavakul (2023)*** (63) | Low | Low | Low | Low | Low | Low | Low |
| ***Sarathi (2016)*** (72) | Unclear | Unclear | High | Unclear | Low | Unclear | Unclear |
| ***Sugino (2022)*** (46) | Low | Low | High | Low | Low | Low | Low |
| ***Tan (2022)*** (35) | Low | Low | High | Low | Low | Unclear | Low |
| ***Trout (2016)*** (47) | Low | Unclear | High | Unclear | Low | Low | Low |
| ***Vinter (2018)*** (62) | Unclear | Unclear | High | Low | Low | Low | Low |
| ***Wang (2015)*** (36) | Low | Unclear | High | Unclear | Low | Unclear | Low |
| ***Wu (2021)*** (37) | Low | Unclear | High | Unclear | Low | Unclear | Low |
| ***Xie (2021)*** (39) | Low | Low | High | Unclear | Low | Unclear | Unclear |
| ***Xie (2022)*** (38) | Low | Low | High | Low | Low | Unclear | Unclear |
| ***Yao (2015)*** (40) | Unclear | Unclear | High | Unclear | Low | Unclear | Low |
| ***Youngwanichsetha (2014)*** (64) | Low | Low | High | Unclear | Low | Unclear | Low |
| ***Zhao (2022)*** (41) | Low | Low | High | Low | Low | Unclear | Unclear |

***Supplementary S6: Characteristics of included studies***

| ***First author (publication year)*** | ***Country*** | ***Sample size (C/I group)*** | ***RCT design*** | ***Definition of GDM*** | ***Mean maternal age, years (mean±SD)*** | ***Gestational age at enrollment (mean±SD; weeks)*** | ***Baseline BMI kg/m^2^ (mean±SD)*** | ***Intervention method*** | ***Duration of intervention (weeks)*** | ***Comparison intervention*** | ***Outcomes*** |
| --- | --- | --- | --- | --- | --- | --- | --- | --- | --- | --- | --- |
| Asemi et al. (2013a & 2013b) (55;56) | Iran | 17/17 | Tow-arm parallel | 100 g OGTT at 24-28 weeks of gestation; fasting >95mg/dl; 1h≥180mg/dl; 2h≥155mg/dl; 3h≥140mg/dl | C:29.4±6.2;  I: 30.7±6.7 | 24-28 | C: 31.4±5.7;  I:29±3.2 | DASH diet (rich in fruits, vegetables, whole grains and low in fat, cholesterol, refined grains and sweets) | 4 | Control diet (45-55% Carb., 15-20% Prot., 25-30% Fat) | FPG; PPG (1h,2h,3h)HbA1C; Insulin; HOMA-IR; maternal weight gain |
| Asemi et al. (2014) (57) | Iran | 26/26 | Tow-arm parallel | 100 g OGTT at 24-28 weeks of gestation; fasting >95mg/dl; 1h≥180mg/dl; 2h≥155mg/dl; 3h≥140mg/dl | C:30.7±6.3;  I: 31.9±6.1 | C: 25.9 ± 1.4  I: 25.8 ± 1.4 | C: 31±4.9;  I:29.2±3.5 | DASH diet (rich in fruits, vegetables, whole grains and low in fat, cholesterol, refined grains and sweets) | 4 | Control diet (45-55% Carb., 15-20% Prot., 25-30% Fat) | CSR; Insulin requirement; Macrosomia; AGW; birth weight, birth length, newborn head circumference, Apgar, PI |

***(Continued)***

***Supplementary S6. Characteristics of included studies (continued)***

| ***First author (year)*** | ***Country*** | ***Sample size (C/I group)*** | ***RCT design*** | ***Definition of GDM*** | ***Mean maternal age, years*** | ***Gestational age at enrollment*** | ***Baseline BMI kg/m^2^ (mean±SD)*** | ***Intervention method*** | ***Duration of intervention (weeks)*** | ***Comparison intervention*** | ***Outcomes*** |
| --- | --- | --- | --- | --- | --- | --- | --- | --- | --- | --- | --- |
| Avery et al. (1997) (42) | USA | 14/15 | Non-blinded parallel | O’Sullivan and Mahan criteria & national consensus screening guidelines for diagnosis of GDM | C: 30.4±5.1  I: 32.2±4.9 | 34 weeks or less | C: 30±5.1  I: 32.2±5.9 | Supervised exercise: Cycle ergometer exercise (5 min warm up & cool-down before and after 20 min exercise, total 30 min/day. twice/week) and Unsupervised exercise (at home): walking or cycling for the same 30min 1-2 times/week; Training HR: 70% of estimated maximal HR[(220 – age) x 0.7]; 3±0.6 times/week | until delivery | Control group (no regular exercise regimen for continuous 30 min more than twice/week); 0.7±0.6 times/week | FPG; PPG (2h breakfast/lunch/dinner); HbA1C; Insulin requirement; maternal weight gain; Birth weight; Macrosomia; birth hypoglycemia;  CSR; AGW; Apgar |
| Awad et al. (2019) (65) | Egypt | 30/30 | Single-blinded parallel | Not reported | C: 29±3.07  I:28.23±2.87 | C: 21.33±1.49  I: 21.33±1.49 | C: 33.83±1.88  I: 33.01±1.73 | Exercise: aerobic + circuits resistance training (10-15 min warm up & cool-down before and after 30 min treadmill aerobic exercise, total 60 min/day; 3-4 times/week), and circuit resistance exercises (2 circuits, each of 10 repetitions with 2 min rest); 60-80% maximal aerobic capacity, and HR no more than 140 beats/min. | until delivery | Control: Diet (2000-2500kcal)+insulin | CSR; Apgar |

***(Continued)***

***Supplementary S6. Characteristics of included studies (continued)***

| ***First author (year)*** | ***Country*** | ***Sample size (C/I group)*** | ***RCT design*** | ***Definition of GDM*** | ***Mean maternal age, years*** | ***Gestational age at enrollment*** | ***Baseline BMI kg/m^2^ (mean±SD)*** | ***Intervention method*** | ***Duration of intervention (weeks)*** | ***Comparison intervention*** | ***Outcomes*** |
| --- | --- | --- | --- | --- | --- | --- | --- | --- | --- | --- | --- |
| Brankston et al. (2004) (59) | Canada | 16/16 | parallel | Canadian Diabetes Association guidelines: 50 g OGL anytime. 1h>185 mg/dl; or 75g OGTT, more than 2 of the following were met: fasting≥95mg/dl; 1h≥191mg/dl;2h≥160mg/dl. | C: 31.3±5  I: 30.5±4.4 | C: 29.6±2.1  I: 29±2 | Not reported | Exercise + diet.  Exercise: circuits resistance training (3 times/week; HR no more than 140 beats/min);  Diet: as same as control group | until delivery | Diet alone (40% Carb.; 20% protein; 40% fat) | Insulin requirement; FPG; PPG (2h breakfast/lunch/dinner/pooled); |
| Christie et al. (2024) (48) | Australia | 14/12 | parallel | International association of diabetes in pregnancy study group criteria | C: 35±4  I: 32±5 | 28-30 | C: 28.2±4.6  I: 28±4.3 | PMW (3x10 min walking, within 60 min of consuming breakfast, lunch and dinner) | Until delivery | Continuous exercise (30 min) | Blood glucose (24h); FPG; PPG(3h breakfast/lunch/dinner); CSR; AGW; birth weigh; birth length; head circumference; newborn hypoglycemia |
| Cypryk et al. (2007) (66) | Poland | 15/15 | parallel | WHO criteria | 28.7±3.7 | 29.2±5.4 | Not reported | Low Carb. (45% Carb., 25% Prot., 30% fat) | 2 | High Carb. (60% Carb., 25% Prot., 15% fat) | FPG; PPG(2h breakfast/lunch/dinner); AGW; CSR; birth weight; Macrosomia; Apgar |

***(Continued)***

***Supplementary S6. Characteristics of included studies (continued)***

| ***First author (year)*** | ***Country*** | ***Sample size (C/I group)*** | ***RCT design*** | ***Definition of GDM*** | ***Mean maternal age, years*** | ***Gestational age at enrollment*** | ***Baseline BMI kg/m^2^ (mean±SD)*** | ***Intervention method*** | ***Duration of intervention (weeks)*** | ***Comparison intervention*** | ***Outcomes*** |
| --- | --- | --- | --- | --- | --- | --- | --- | --- | --- | --- | --- |
| de Barros et al. (2010) (67) | Brazil | 32/32 | parallel | 100g OGTT in 3h; or 75g OGTT in 2h. fasting ≥95mg/dl; 2h≥120mg/dl | C: 32.4±5.4  I: 31.81±4.87 | C: 31.06±2.3  I: 31.56±2.29 | C: 29.43±4.01  I: 29.61±4.13 | Circuits resistance training (elastic band; start about 90min after a meal; 15 repetitions for each exercise with 30s-1min rest; 2 circuit sessions/day at 1st and 2nd week, and 3 circuit sessions from 3rd week to the end of gestation; 3 sessions/week with 1 session supervised and 2 sessions at home) | Until delivery | Control (35 kcal/kg/day, and 300 kcal/day were added in the 2^nd^ and 3^rd^ trimesters) | Insulin requirement; blood glucose (not 24h); FPG; AGW; maternal weight gain; birth weight |
| Grant et al. (2011) (60) | Canada | 22/21 | Parallel | Diagnosed based on 1-hour plasma glucose ≥185.4 mg/dL after 50g glucose load, or 2 of the following values after 75g OGTT: fasting ≥95.4 mg/dL, 1-hour ≥190.8 mg/dL, 2-hour ≥160.2 mg/dL | C: 34±2.3; I: 34±0.4 | C: 29±1.54; I:29±1.47 | Not reported | Low GI group received low GI foods. | From enrollment until delivery, up to 8 weeks | Control group received intermediate and high GI foods. | FPB; PPG; Insulin requirement; maternal weight gain; birth weight; macrosomia; |

***(Continued)***

***Supplementary S6. Characteristics of included studies (continued)***

| ***First author (year)*** | ***Country*** | ***Sample size (C/I group)*** | ***RCT design*** | ***Definition of GDM*** | ***Mean maternal age, years*** | ***Gestational age at enrollment*** | ***Baseline BMI kg/m^2^ (mean±SD)*** | ***Intervention method*** | ***Duration of intervention (weeks)*** | ***Comparison intervention*** | ***Outcomes*** |
| --- | --- | --- | --- | --- | --- | --- | --- | --- | --- | --- | --- |
| Halse et al. (2014 & 2015) (49;50) | Australia | 20/20 | Parallel | Fasting venous plasma glucose ≥99mg/dl or 75-g OGTT 2h venous plasma glucose ≥144mg/dl | C: 32±3; I: 34±5 | C:28.8±1  I: 28.8±0.8 | Not reported | Home-based cycling ergometer program: 3 supervised sessions (25-45 min, 65-85% age predicted HRmax, RPE 12-16) + 2 unsupervised 30 min moderate sessions per week | 6 ± 1 weeks | Conventional management with diet counseling and glucose monitoring | FPG; PPG (2h, breakfast/lunch/dinner; pooled); HbA1c; blood glucose; insulin; Maternal weight gain; CSR; AGW; preterm birth; Brith weight; birth length; head circumference; Apgar |
| Hernandez et al. (2016) (44) | USA | 6/6 | Parallel | Diagnosed at 24-28 weeks using Carpenter and Coustan criteria | C: 28 ± 4.9  I: 30 ± 2.45 | C: 31.2 ± 0.82  I: 31.7 ± 2.45 | C: 33.4 ± 3.4  I: 34.3 ± 3.27 | Higher-complex carbohydrate /lower-fat Choosing Healthy Options In Carbohydrate Energy diet: 60% Carb./25% fat/15% prot. | 7 weeks (until delivery) | higher-fat conventional diet: 40% Carb./45% fat/15% protein | FPG; Insulin; HOMA-IR; AWG; maternal weight gain; CSR; birth weight; birth length |
| Hernandez et al. (2023) (43) | USA | 23/23 | Parallel | Women with GDM meeting criteria by a 50-g glucose challenge followed by a 3-h 100-g OGTT according to the American College of Obstetricians and Gynecologists | C: 32 ± 4.78  I: 33 ± 4.78 | C: 31.7 ± 0.48  I: 32.3 ± 0.48 | C:32 ± 4.78  I: 32 ± 4.78 | Higher-complex carbohydrate /lower-fat Choosing Healthy Options In Carbohydrate Energy diet: 60% Carb./25% fat/15% protein | 7-8 weeks | higher-fat conventional diet: 40% Carb./45% fat/15% protein | CSR; birth weight; birth length; head circumference; AGW; maternal weight gain; LGA; SGA; newborn hypoglycemia; PI; FPG; PPG (1h/2h); blood glucose (24h); HOMA-IR; Insulin |

***(Continued)***

***Supplementary S6. Characteristics of included studies (continued)***

| ***First author (year)*** | ***Country*** | ***Sample size (C/I group)*** | ***RCT design*** | ***Definition of GDM*** | ***Mean maternal age, years*** | ***Gestational age at enrollment*** | ***Baseline BMI kg/m^2^ (mean±SD)*** | ***Intervention method*** | ***Duration of intervention (weeks)*** | ***Comparison intervention*** | ***Outcomes*** |
| --- | --- | --- | --- | --- | --- | --- | --- | --- | --- | --- | --- |
| Jamilian and Asemi (2015) (58) | Iran | 34/34 | Parallel | Diagnosed by a "one-step" 2-hour 75-g oral glucose tolerance test based on American Diabetes Association criteria: fasting plasma glucose ≥ 92 mg/dL, 1-hour value ≥ 180 mg/dL, or 2-hour value ≥ 153 mg/dL | C: 29.3 ± 4.2  I: 28.2 ± 4.6 | 24-28 | C: 28.4 ± 3.4  I: 28.9 ± 5 | Soy protein group received a diet containing 0.8 g/kg protein with 35% animal protein, 35% soy protein (textured soy protein from Sobhan company), and 30% other plant proteins | 6 weeks | Control group received a diet containing 0.8 g/kg protein with 70% animal protein and 30% plant proteins | FPG; insulin; HOMA-IR; insulin requirement; preterm birth; CSR; macrosomia; AWG; birth weight; birth length; head circumference; Apgar; newborn hypoglycemia |
| Jin et al. (2022) (34) | China | 66/65 | Parallel | Diagnosed according to the criteria of the International Association of Diabetes and Pregnancy Study Groups (fasting blood glucose ≥91 mg/dL, 1-h blood glucose ≥180 mg/dL, or 2-h blood glucose ≥153 mg/dL in a 75-g OGTT) | C: 32.14 ± 4.39  I: 33.51 ± 4.27 | C: 25.91 ± 1.39  I: 25.66 ± 1.58 | Not reported | The original Gymnastics for Pregnant Women program lasted 15 min and included 8 sections: Warm-up, Finger stretch, Arm stretch, Fluttering of arms like butterflies, Stretching like a bird in flight, Swinging arms in bow step, Waving arms in the "Lucky Cat" style, and Cool down. Participants performed it 10 times per week. | Until delivery | Conventional intervention: standardized GDM health education including medical nutrition therapy, exercise guidance, and self-monitoring of blood glucose | FPG; PPG (2h); CSR; HbA1c; maternal weight gain; premature birth; newborn hypoglycemia; birth weight; macrosomia |

***(Continued)***

***Supplementary S6. Characteristics of included studies (continued)***

| ***First author (year)*** | ***Country*** | ***Sample size (C/I group)*** | ***RCT design*** | ***Definition of GDM*** | ***Mean maternal age, years*** | ***Gestational age at enrollment*** | ***Baseline BMI kg/m^2^ (mean±SD)*** | ***Intervention method*** | ***Duration of intervention (weeks)*** | ***Comparison intervention*** | ***Outcomes*** |
| --- | --- | --- | --- | --- | --- | --- | --- | --- | --- | --- | --- |
| Jovanovic-Peterson et al. (1989) (45) | USA | 9/10 | Parallel | Diagnosed according to standard protocol from second international Workshop-Conference on Gestational Diabetes Mellitus | C: 31.1 ± 2.8  I: 29.5 ± 2.5 | Not reported | Not reported | Exercise (20 minutes three times a week for 6 weeks) using an arm ergometer to maintain heart rate in the training range. | 6 weeks | Control: Diet alone (24 to 30 kcal/kg/24 hours; 20% protein, 40% carbohydrate, 40% fat) | FPG; PPG (1h); HbA1c; AWG; birth weight |
| Kokic et al. (2017) (68) | Croatia | 20/18 | Parallel | Diagnosed according to criteria published by the International Association of the Diabetes and Pregnancy Study Groups | C: 31.95 ± 4.91  I: 32.78 ± 3.83 | C: 20.8 ± 6.05  I: 22.44 ± 6.55 | Not reported | Structured exercise program 2x/week (20 min aerobic, 20-25 min resistance, pelvic floor/stretching) plus ≥30 min daily brisk walking. Aerobic target 65-75% max HR. Resistance with bands/weights, 3 sets of 10-15 reps for major muscle groups. | Minimum 6 weeks, until delivery | Standard antenatal care for GDM | FPG; PPG (3h); AWG; CSR; Apgar; PI; newborn hypoglycemia; birth length; birth weight |
| Lauszus et al. (2001) (61) | Denmark | 12/13 | Parallel, unpaired | GDM was defined as two or more plasma glucose samples above three standard deviations of the mean from a 3-hour 75g OGTT | C: 29±3.46  I: 31±3.61 | 33 | C: 32.2±4.9  I: 35.3±7.85 | High monounsaturated fatty acids diet: Source was a hybrid sunflower oil with high oleic acid (80%) content. Snacks were almonds and hazelnuts. | 5 weeks | High Carb. diet: Enriched with bread, potato and rice. | PPG; HbA1c; FPG; Insulin sensitivity and response; birth weight |

***(Continued)***

***Supplementary S6. Characteristics of included studies (continued)***

| ***First author (year)*** | ***Country*** | ***Sample size (C/I group)*** | ***RCT design*** | ***Definition of GDM*** | ***Mean maternal age, years*** | ***Gestational age at enrollment*** | ***Baseline BMI kg/m^2^ (mean±SD)*** | ***Intervention method*** | ***Duration of intervention (weeks)*** | ***Comparison intervention*** | ***Outcomes*** |
| --- | --- | --- | --- | --- | --- | --- | --- | --- | --- | --- | --- |
| Louie et al. (2011) (51) | Australia | 47/45 | Parallel | Diagnosed by 75-g oral glucose tolerance test at 20-32 weeks based on modified Australasian Diabetes in Pregnancy Society criteria: fasting BGL ≥99 mg/dL, 1-h BGL ≥180 mg/dL, or 2-h BGL ≥144 mg/dL | C: 34±4.1  I: 32.4±4.5 | C: 29±4  I: 29.7±3.5 | Not reported | High fiber group: Target glycemic index ~60 (moderate), high fiber content, similar macronutrient composition as LGI group. | 6-7 weeks | Low glycemic index group: Target glycemic index ≤50, diet of 15-25% protein, 25-30% fat, 40-45% carbs. | Blood glucose; Insulin; HbA1c; HOMA2-IR; AWG; birth weight; birth length; head circumference; maternal weight gain; insulin requirement; LGA; SGA; macrosomia; PI; CSR |
| Lv et al. (2019) (32) | China | 67/67 | Parallel | IADPSG in 2010, the oral glucose tolerance test (OGTT) using 75g glucose considered that any of the following thresholds should be met or exceeded: fasting plasma glucose, 92 mg/dL; 1-hour plasma glucose, 180 mg/dL; and 2-hour plasma glucose, 153 mg/dL. | Not reported | Not reported | Not reported | Nutritional nursing intervention based on GL: The fixed nutrition recipes of the GL group adjusted the heat of food by the GL, where GL = GI × carbohydrate content/100. | Until delivery | Conventional nutritional nursing based on traditional food exchange method | FPG; PPG (2h); macrosomia; premature birth |

***(Continued)***

***Supplementary S6. Characteristics of included studies (continued)***

| ***First author (year)*** | ***Country*** | ***Sample size (C/I group)*** | ***RCT design*** | ***Definition of GDM*** | ***Mean maternal age, years*** | ***Gestational age at enrollment*** | ***Baseline BMI kg/m^2^ (mean±SD)*** | ***Intervention method*** | ***Duration of intervention (weeks)*** | ***Comparison intervention*** | ***Outcomes*** |
| --- | --- | --- | --- | --- | --- | --- | --- | --- | --- | --- | --- |
| Ma et al. (2014) (33) | China | 42/41 | Parallel | Screened with 50g glucose challenge test, diagnosed if met >=2 criteria: fasting >=104.4mg/dl, 1h >=190.8mg/dl, 2h >=165.6mg/dl, 3h >=145.8mg/dl | C: 30±3.5  I: 30.1±3.8 | C: 27.9±1.1  I: 27.5±1.1 | Not reported | Low-GL group received low glycemic load diet advice with food exchange lists containing low-GL foods every 2 weeks | 12-14 weeks until delivery | Control group received general dietary advice focused on energy, carbohydrate, protein and fat control every 2 weeks. 45-50% Carb., 20-24% Prot., 25-30% fat. | FPG; PPG (2h); HbA1c; maternal weight gain; birth weight; macrosomia; preterm birth; |
| Menek et al. (2024) (69) | Turkey | 15/15/15 | Parallel, single-blind | Diagnosed based on 75g oral glucose tolerance test between 24-28 weeks with fasting plasma glucose >92 mg/dL or 1-hr >180 mg/dL or 2-hr >153 mg/dL | C1: 34.18±2.16  C2:36.85±2.79  I: 35.06±4.31 | 24-28 | Not reported | Supervised home exercise group: home exercise program was taught and controlled by a physiotherapist. Low-moderate structured aerobic, resistance, stretching 3 days/week. | 8 weeks | 1.Home exercise group: without gynecologist.  2.Control group: maintained usual daily care | FPG; PPG (2h); CSR; preterm birth |

***(Continued)***

***Supplementary S6. Characteristics of included studies (continued)***

| ***First author (year)*** | ***Country*** | ***Sample size (C/I group)*** | ***RCT design*** | ***Definition of GDM*** | ***Mean maternal age, years*** | ***Gestational age at enrollment*** | ***Baseline BMI kg/m^2^ (mean±SD)*** | ***Intervention method*** | ***Duration of intervention (weeks)*** | ***Comparison intervention*** | ***Outcomes*** |
| --- | --- | --- | --- | --- | --- | --- | --- | --- | --- | --- | --- |
| Mijatovic et al. (2020) (52) | Australia | 17/16 | Parallel, two-arm | Diagnosed by 75-g OGTT using either 1998 ADIPS criteria (fasting ≥99mg/dl or 2h ≥144mg/dl) or 2010 IADPSG criteria (fasting ≥92mg/dl, 1h ≥180mg/dl, or 2h ≥153mg/dl) | C: 34.2 ± 3.04  I: 32.5 ± 2.88 | C: 28.6 ± 2.46  I: 28.4 ± 2 | Not reported | MLC diet: Target 135 g/day carbohydrate without energy restriction | 6 weeks | RC diet: Target 180-200 g/day carbohydrate | FPG; HbA1c; PPG; AWG; maternal weight gain; CSR; birth weight; LGA; SGA; macrosomia |
| Moreno-Castilia et al. (2013) (70) | Spain | 75/75 | Parallel | Diagnosed following the 2006 National Diabetes and Pregnancy Clinical Guidelines in Spain, using 100g oral glucose tolerance test and National Diabetes Data Group criteria | C: 32.1 ± 4.4  I: 33.5 ± 3.7 | C: 30.1 ± 3.5  I: 30.4 ± 3 | Not reported | Low-carbohydrate diet: 40% of total calories from carbohydrates, 20% protein, 40% fat. | Until delivery | Control diet: 55% of total calories from carbohydrates, 20% protein, 25% fat. | AWG; insulin requirement; maternal weight gain; CSR; LGA; SGA; macrosomia; newborn hypoglycemia |

***(Continued)***

***Supplementary S6. Characteristics of included studies (continued)***

| ***First author (year)*** | ***Country*** | ***Sample size (C/I group)*** | ***RCT design*** | ***Definition of GDM*** | ***Mean maternal age, years*** | ***Gestational age at enrollment*** | ***Baseline BMI kg/m^2^ (mean±SD)*** | ***Intervention method*** | ***Duration of intervention (weeks)*** | ***Comparison intervention*** | ***Outcomes*** |
| --- | --- | --- | --- | --- | --- | --- | --- | --- | --- | --- | --- |
| Moses et al (2009) (53) | Australia | 32/31 | Parallel | Australasian Diabetes in Pregnancy Society (ADIPS) criteria: fasting glucose ≥100 mg/dl and/or 2-h glucose ≥145 mg/dl after a 75-g glucose load | C:31.3±4.53  I: 30.8±3.89 | C: 29.9± 1.13  I: 30.3±1.12 | C:32.8±7.98  I: 32.0±6.62 | Low-GI diet based on previously verified low-GI foods, including pasta, grain breads, and unprocessed breakfast cereals with high fiber content. Advised to avoid white bread, processed breakfast cereals, potatoes, and some rice varieties. | until delivery | High GI group: High-fiber/low-sugar diet with no specific mention of GI. Potatoes, whole wheat bread, and specific high-fiber moderate-to-high GI breakfast cereals were recommended. | Insulin |
| Perichart-Perera et al. (2012) (71) | Mexico | 46/61 | Parallel | GDM diagnosed with 2 or more abnormal values (fasting ≥95 mg/dL, 1h ≥180 mg/dL, 2h ≥155 mg/dL, 3h ≥140 mg/dL) after 100g OGTT if 50g 1-hr glucose ≥130 mg/dL | C: 31.8 ± 5.3  I: 32.3 ± 4.8 | C: 20.7 ± 6.7  I: 22.5 ± 4.9 | Not reported | Low GI diet - excluded foods with GI>55, 40-45% CHO, moderate energy restriction for overweight/obese, CHO counting, fiber 20-35g/day | Until end of pregnancy | All types of CHO diet - 40-45% CHO, moderate energy restriction for overweight/obese, CHO counting, fiber 20-35g/day | FPG; PPG (2h breakfast/lunch/dinner); insulin use; maternal weight gain; macrosomia; SGA; LGA; |

***(Continued)***

***Supplementary S6. Characteristics of included studies (continued)***

| ***First author (year)*** | ***Country*** | ***Sample size (C/I group)*** | ***RCT design*** | ***Definition of GDM*** | ***Mean maternal age, years*** | ***Gestational age at enrollment*** | ***Baseline BMI kg/m^2^ (mean±SD)*** | ***Intervention method*** | ***Duration of intervention (weeks)*** | ***Comparison intervention*** | ***Outcomes*** |
| --- | --- | --- | --- | --- | --- | --- | --- | --- | --- | --- | --- |
| Rae et al. (2000) (54) | Australia | 54/63 | Parallel | Oral glucose tolerance test with fasting plasma glucose ≥92 mg/dL, and/or 2 hour plasma glucose ≥144 mg/dL | C: 30.6 ± 5.6  I: 30.2 ± 5.8 | C: 28.3 ± 4.6  I: 28.1 ± 5.8 | C: 38 ± 0.7  I: 37.9 ± 0.7 | Moderately energy restricted diabetic diet providing 6800-7600 kJ (1590-1776 kcal) per day, representing 70% of recommended dietary intake | until delivery | Diabetic diet not energy restricted, providing 8600-9500 kJ (2010-2220 kcal) per day | FPG; PPG (breakfast/lunch/dinner); HbA1c; AWG; birth weight; insulin use; CSR |
| Sanpawithayakul et al. (2023) (63) | Thailand | 48/48 | Triple-blind Parallel | Two-step approach: 50-g OGTT, followed by 100-g OGTT if blood glucose ≥140 mg/dL. GDM diagnosed if ≥2 values met Carpenter and Coustan criteria. | C: 33.6 ± 4.1  I: 33.1 ± 13.1 | C: 26.3 ± 4.9  I: 25.7 ± 5.5 | Not reported | RD43 rice (low to moderate GI rice, GI=56.9), consumed daily, amount based on ideal body weight and gestational age | 4 weeks | THM rice (high GI rice, GI=80.1), consumed daily, amount based on ideal body weight and gestational age | FPG; PPG (1h,2h); insulin requirement; |
| Sarathi et al. (2016) (72) | India | 30/32 | Parallel | Using International association of the diabetes and pregnancy study groups (IADPSG) criteria | C: 29.17±3.38  I: 29.43±2.98 | C:25.56±1.69  I:25.19±1.92 | Not reported | Soya based protein rich diet: Same as control diet except 25% of cereal replaced by soya (beans, chunks, granules or flour) in each major meal. Total calories 1600-2000 kcal/day, min carbohydrate 175 g/day. | Until delivery | High fiber complex carbohydrate diet: Total calories 1600-2000 kcal/day, min carbohydrate 175 g/day. 3 major meals (350-400 kcal) and 3-4 snacks (150-200 kcal). | AWG; CSR; FPG; PPG (2h breakfast/lunch/dinner); HbA1c; birth weight; LGA; maternal weight gain; Insulin requirement; newborn hypoglycemia |

***(Continued)***

***Supplementary S6. Characteristics of included studies (continued)***

| ***First author (year)*** | ***Country*** | ***Sample size (C/I group)*** | ***RCT design*** | ***Definition of GDM*** | ***Mean maternal age, years*** | ***Gestational age at enrollment*** | ***Baseline BMI kg/m^2^ (mean±SD)*** | ***Intervention method*** | ***Duration of intervention (weeks)*** | ***Comparison intervention*** | ***Outcomes*** |
| --- | --- | --- | --- | --- | --- | --- | --- | --- | --- | --- | --- |
| Sugino et al.(2022) (46) | USA | 16/18 | Parallel | Carpenter and Coustan criteria | C: 32.96 ± 3.08  I: 31.96 ± 4.77 | 30-31 weeks | C: 31.47 ± 4.95  I: 32.93 ± 5.69 | CHOICE diet: 60% complex carbohydrate/25% fat/15% protein, eucaloric, similar fiber (~29.3 g/day), fat percentage matched, simple sugars ≤18% kcal. | until delivery | CONV diet: 40% complex carbohydrate/45% fat/15% protein, eucaloric, similar fiber (~23.5 g/day), fat percentage matched, simple sugars ≤18% kcal. | AWG; birth weight; FPG; Insulin; HOMA-IR |
| Tan et al. (2022) (35) | China | 50/53 | Single-blinded parallel | Diagnosed with GDM according to the diagnostic criteria recommended by the International Diabetes and Pregnancy and Research Group | C:33.24 ± 4.59  I:31.91±3.95 | 24-28 weeks | Not reported | Exercise plan consisting of aerobic (brisk walking, exercise for pregnant women) and resistance exercise (upper limb resistance exercise). Moderate-intensity exercise for 30 min at least 5 times a week. | 12 weeks | Usual exercise program delivered by diabetes specialist nurses. | FPG; PPG (2h); macrosomia; CSR; birth weight; newborn hypoglycemia |

***(Continued)***

***Supplementary S6. Characteristics of included studies (continued)***

| ***First author (year)*** | ***Country*** | ***Sample size (C/I group)*** | ***RCT design*** | ***Definition of GDM*** | ***Mean maternal age, years*** | ***Gestational age at enrollment*** | ***Baseline BMI kg/m^2^ (mean±SD)*** | ***Intervention method*** | ***Duration of intervention (weeks)*** | ***Comparison intervention*** | ***Outcomes*** |
| --- | --- | --- | --- | --- | --- | --- | --- | --- | --- | --- | --- |
| Trout et al. (2016) (47) | USA | 31/37 | Parallel | Diagnosed based on Carpenter-Coustan criteria after a 1-hour 50g OGTT screening (≥135 mg/dL) followed by a 100g OGTT | C: 29.63 ± 5.19  I: 30.09 ± 6.15 | C:30.50 ± 2.85  I: 29.17 ± 2.78 | C: 31.80 ± 8.68  I: 33.84 ± 8.84 | Lower-carbohydrate diet (35-40% of total calories). | until delivery | Usual pregnancy diet (50-55% carbohydrates) | PPG (2h); FPG; CSR; AWG; birth weight; head circumference; macrosomia; maternal weight gain; insulin requirement; |
| Vinter et al. (2018) (62) | Denmark | 54/36 | Parallel | WHO 2013 GDM criteria: fasting venous plasma glucose ≥92 mg/dL and/or 2-h capillary blood glucose ≥153 mg/dL after a 75-g OGTT | C: 30 (27-32)  I: 29 (27-34) | 12-15 | C: 34.6 (32.7-37.3)  I: 34.3 (32.3-39.2) | Physical activity - encouraged 30-60 min moderate activity daily, provided pedometer, and free gym membership for 6 months with 1-hour weekly physiotherapist-led exercise classes. | Throughout pregnancy | Standard care | preterm birth; CSR; LGA; |
| Wang et al. (2015) (36) | China | 43/41 | Parallel | A 75g OGTT was performed at 24-28 weeks of pregnancy. Glucose levels after fasting, and 1 and 2h after glucose administration <92, <180, and <153 mg/dL respectively were considered normal. | C:29.7±4.64  I:30.3±4.17 | C:27.3±1.96  I:27.4±1.52 | Not reported | Oil-rich diet with 45-50g sunflower oil daily, carbohydrates 50-54%, fat 31-35% of total energy | until delivery | Low-oil diet with 20g sunflower oil daily, carbohydrates 55-60%, fat 25-30% of total energy | FBG; PPG (2h); AWG; preterm birth; birth weight; macrosomia; premature birth; insulin requirement; maternal weight gain |

***(Continued)***

***Supplementary S6. Characteristics of included studies (continued)***

| ***First author (year)*** | ***Country*** | ***Sample size (C/I group)*** | ***RCT design*** | ***Definition of GDM*** | ***Mean maternal age, years*** | ***Gestational age at enrollment*** | ***Baseline BMI kg/m^2^ (mean±SD)*** | ***Intervention method*** | ***Duration of intervention (weeks)*** | ***Comparison intervention*** | ***Outcomes*** |
| --- | --- | --- | --- | --- | --- | --- | --- | --- | --- | --- | --- |
| Wu et al. (2021) (37) | China | 70/68 | Parallel | The diagnosis of GDM was based on the diagnostic criteria published by the International Expert Group on Gestational Diabetes (IADPSG) in 2010. | C: 28.77 ± 4.01  I: 28.75 ± 3.93 | 24-28 | C: 21.84 ± 2.00  I: 22.00 ± 1.94 | Diversified and quantitative management model of exercise intervention, including aerobic exercise mainly, combined with resistance exercise. Moderate intensity exercise with accumulative time of at least 30 min per day and 150 min per week was encouraged. Specific implementations included structured courses, audio-visual materials, individualized exercise therapy recommendation, and WeChat follow-up. | Until delivery (at least 4 weeks) | Conventional sports and health education during pregnancy, with regular exercise classes at the pregnant women's school until the end of childbirth. | FPG; PPG (2h); macrosomia; CSR; maternal weight gain; newborn hypoglycemia; preterm birth |
| Xie et al. (2021) (39) | China | 46/43 | Parallel | Diagnosed based on the Oral Glucose Tolerance Test (OGTT) published by The International Association of Diabetes and Pregnancy Study Groups (IADPSG) in 2010 | C: 31.35 ± 4.72  I: 31.47 ± 4.06 | C: 28.02 ± 2.30  I: 28.14 ± 2.00 | Not reported | Moderate-intensity aerobic exercise: 50-60 mins, 3 times/week for at least 6 weeks. Exercise included steps, neck/arm/leg movements guided by sports medicine experts. (with Routine care) | At least 6 weeks | Control group: Routine care: Personalized diabetes diet intervention, online education, routine prenatal care | FPG; PPG (2h); CSR; macrosomia; birth weight; birth length; Apgar; insulin requirement; preterm birth; newborn hypoglycemia |

***(Continued)***

***Supplementary S6. Characteristics of included studies (continued)***

| ***First author (year)*** | ***Country*** | ***Sample size (C/I group)*** | ***RCT design*** | ***Definition of GDM*** | ***Mean maternal age, years*** | ***Gestational age at enrollment*** | ***Baseline BMI kg/m^2^ (mean±SD)*** | ***Intervention method*** | ***Duration of intervention (weeks)*** | ***Comparison intervention*** | ***Outcomes*** |
| --- | --- | --- | --- | --- | --- | --- | --- | --- | --- | --- | --- |
| Xie et al. (2022) (38) | China | 43/43 | Parallel | Diagnosed based on the oral glucose tolerance test, according to the International Association of Diabetes and Pregnancy Study Groups 2010 criteria | C: 31.47±4.06  I: 31.84±5.19 | C: 28.14±2.00  I: 28.02±2.01 | Not reported | Resistance exercises for upper and lower limb muscle training, including elbow flexion exercise, ankle extension exercise, resistance exercise of the upper limb, leg lift exercise, upper limb dorsiflexion exercise, and leg abduction exercise. Moderate intensity, 50-60 minutes, 3 times per week. | 6 weeks | Aerobic exercise mainly included step walking, neck stretching exercise, arm stretching exercise, leg exercise. Moderate intensity, 50-60 minutes, 3 times per week. | FPG; PPG (2h); preterm birth; CSR; birth weight; birth length; macrosomia; Apgar |
| Yao et al. (2015) (40) | China | 16/17 | Parallel | Diagnosed by a 100-g oral glucose tolerance test during pregnancy for 24-28 weeks. GDM defined as meeting 2 of the following criteria: fasting >95 mg/dL, 1-hour ≥180 mg/dL, 2-hour ≥155 mg/dL, 3-hour ≥140 mg/dL | C: 28.3 ± 5.1  I: 30.7 ± 5.6 | C: 25.7 ± 1.3  I: 26.9 ± 1.4 | C: 30.9 ± 3.6  I: 30.2 ± 4.1 | DASH diet: High in fruits, vegetables, whole grains, low-fat dairy, low saturated fats, cholesterol, refined grains and sweets. 2400 mg sodium/day. | 4 weeks | Control diet: 45-55% carbs, 15-20% protein, 25-30% total fat | AWG; CSR; macrosomia; birth weight; birth length; head circumference; PI; Apgar; FPG; Insulin requirement; insulin; HOMA-IR |

***(Continued)***

***Supplementary S6. Characteristics of included studies (continued)***

| ***First author (year)*** | ***Country*** | ***Sample size (C/I group)*** | ***RCT design*** | ***Definition of GDM*** | ***Mean maternal age, years*** | ***Gestational age at enrollment*** | ***Baseline BMI kg/m^2^ (mean±SD)*** | ***Intervention method*** | ***Duration of intervention (weeks)*** | ***Comparison intervention*** | ***Outcomes*** |
| --- | --- | --- | --- | --- | --- | --- | --- | --- | --- | --- | --- |
| Youngwanichsetha et al (2014) (64) | Thailand | 85/85 | Parallel | Diagnosed with gestational diabetes mellitus (GDM) class A1 with 24-30 weeks gestational age, having fasting blood glucose < 105 mg/dL, and 2-h postprandial blood glucose < 120 mg/dL | C: 31.24 ± 4.54  I: 32.58 ± 5.01 | 24-30 weeks | C: 27.05 ± 4.06  I: 27.09 ± 3.56 | Mindfulness eating: 5 steps - set goal, integrate nutrition therapy, consider portion size, be aware while eating, eat slowly for 30-45 mins.  Yoga exercise: 9 postures for 15-20 mins daily, 5 days/week | 8 weeks | Standard diabetes care | FPG; PPG(2h); HbA1c |
| Zhao et al. (2022) (41) | China | 46/43 | Parallel | Diagnosed according to Oral Glucose Tolerance Test published by International Association of Diabetes and Pregnancy study groups | C: 31.35 ± 4.72  I: 31.84 ± 5.19 | C:28.02 ± 2.3  I: 28.02 ± 2.01 | Not reported | Moderate-intensity resistance exercise including elbow flexion, ankle extension, upper/lower limb resistance, leg lifts, upper limb dorsiflexion, leg abduction. 50-60 mins, 3 times/week, on-site supervision, exercise intensity monitored by heart rate (≤220-age) and Borg scale (13-14). | At least 6 weeks | Routine prenatal care, online education, personalized diabetes diet | FPG; PPG (2h); Insulin requirement; maternal weight gain; preterm birth; CSR; macrosomia; birth weight; birth length; newborn hypoglycemia; Apgar |

***AGW:*** average gestational week at delivery; ***BMI:*** Body Mass Index; ***bpm:*** beats per minute; ***C:*** Comparison group; ***Carb***.: Carbohydrates; ***CGM:*** continuous glucose monitoring; ***CSR:*** Caesarean section rate; ***DASH:*** Dietary Approaches to Stop Hypertension; ***FPG:*** Fasting Plasma Glucose; ***GDM:*** gestational diabetes mellitus; ***GI:*** glycemic-index; ***GL***: glycemic load; ***HR:*** Heart rate; ***I:*** Intervention group; ***LGA:*** Large for gestational age; min: minutes; s:second; ***MLC:*** modestly lower carbohydrate diet; ***NFI:*** Need for insulin therapy after intervention; ***OGL:*** oral glucose load; ***OGTT:*** Oral Glucose Tolerance Test; ***PI:*** Ponderal index; ***PMW:*** Post-meal walking; ***PPG:*** Postprandial Glucose; ***Prot.:*** Protein; ***RC:*** routine care; ***SD:*** Standard Deviation;

***Supplementary S7: Model Selection***

| *Measures* | *Items* | *Fixed Model* | *Random Model* |
| --- | --- | --- | --- |
| *Fasting Glucose* | Dbar | 195.82  (56 data points) | 56.24  (56 data points) |
|  | pD | 42.95 | 54.93 |
|  | DIC | 238.77 | 111.17 |
|  | I^2^ | 72% | 2% |
| *2h Postprandial Glucose* | Dbar | 47.58  (40 data points) | 41.67  (40 data points) |
|  | pD | 33.00 | 37.48 |
|  | DIC | 80.58 | 79.15 |
|  | I^2^ | 18% | 6% |
| *Insulin Requirement* | Dbar | 43.45  (44 data points) | 43.40  (on 44 data points) |
|  | pD | 34.35 | 36.35 |
|  | DIC | 77.80 | 79.75 |
|  | I^2^ | 1% | 0.9% |
| *Birth Weight* | Dbar | 56.04  (56 data points) | 53.57  (56 data points) |
|  | pD | 42.03 | 45.59 |
|  | DIC | 98.07 | 99.15 |
|  | I^2^ | 2% | 0% |
| *Macrosomia* | Dbar | 42.66  (on 44 data points) | 40.12  (on 44 data points) |
|  | pD | 33.74 | 36.72 |
|  | DIC | 76.40 | 76.84 |
|  | I^2^ | 0% | 0% |
| *Preterm Birth* | Dbar | 29.77  (on 30 data points) | 27.48  (on 30 data points) |
|  | pD | 20.71 | 23.54 |
|  | DIC | 50.48 | 51.02 |
|  | I^2^ | 3% | 0% |

D_bar_, representing the goodness of fit, was utilized to assess the model fit in this NMA.

pD, the effective number of parameters, was employed to characterize the complexity of the models.

DIC (Deviance Information Criterion) = pD + D_bar_ , served as an overall measure of model fit.

We combined DIC and I² for model selection. Specifically, if I² < 50%, indicating low or moderate heterogeneity, and the difference in DIC between the fixed-effects and random-effects models is less than 3, we will prioritize the fixed-effects model. If the difference in DIC is greater than or equal to 3, we will choose the model with the smaller DIC. If I²≥ 50%, indicating high heterogeneity, we will select the random-effects model, unless the fixed-effects model has a substantially smaller DIC (with a difference greater than or equal to 3).

***Supplementary S8: Global inconsistency and heterogeneity assessment***

| *Measures* | *Items* | *Consistency Model* | *Inconsistency Model* |
| --- | --- | --- | --- |
| *Fasting Glucose* | Dbar | 56.20  (56 data points) | 56.01  (56 data points) |
|  | pD | 54.91 | 55.35 |
|  | DIC | 111.10 | 111.36 |
|  | I^2^ | 2% | 2% |
| *2h Postprandial Glucose* | Dbar | 47.57  (40 data points) | 45.04  (40 data points) |
|  | pD | 32.99 | 35.03 |
|  | DIC | 80.56 | 80.07 |
|  | I^2^ | 18% | 13% |
| *Insulin Requirement* | Dbar | 43.52  (44 data points) | 44.59  (44 data points) |
|  | pD | 34.43 | 35.68 |
|  | DIC | 77.95 | 80.27 |
|  | I^2^ | 1% | 4% |
| *Birth Weight* | Dbar | 56.08  (56 data points) | 54.42  (56 data points) |
|  | pD | 42.07 | 44.95 |
|  | DIC | 98.15 | 99.37 |
|  | I^2^ | 2% | 0% |
| *Macrosomia* | Dbar | 42.65  (44 data points) | 43.76  (44 data points) |
|  | pD | 33.74 | 34.89 |
|  | DIC | 76.38 | 78.65 |
|  | I^2^ | 0% | 2% |
| *Preterm Birth* | Dbar | 29.74  (30 data points) | 30.34  (30 data points) |
|  | pD | 20.68 | 21.94 |
|  | DIC | 50.43 | 51.98 |
|  | I^2^ | 3% | 3% |

We compared the consistent and inconsistent models. When I^2^≥50%, it was considered to have network heterogeneity, and when the difference between DICs was greater than 3, it was considered to have global inconsistency.

***Supplementary S9: Rank Probability and SUCRA Values***

*AE: Aerobic Exercise; ARE: Aerobic + Resistance Exercise; CON: Control; DAS: DASH; ER: Energy Restriction; HC: High Carbohydrates; HCC: High Complex Carbohydrates; HF: High Fiber; HFL: Higher fat/Lower Carbohydrates; HGI: High GI/GL; HMU: High MUFA (Monounsaturated Fatty Acid); LC: Low Carbohydrates; LGI: Low GI/GL; LGY: Low GI + Yoga; PMW: Postmeal Walking; RE: Resistance Exercise; SE: Structured Exercise; SOY: Soy Protein;*

*Green color represents values that have a probability exceeding 5% (0.05).*

*Blue color represents the highest rank probability for each group.*

*Red color represents Zero Event.*

***FASTING PLASMA GLUCOSE***

| *Rank* | *PMW* | *SE* | *DAS* | *Soy* | *AE* | *ARE* | *LGY* | *HCC* | *LGI* | *RE* | *HC* | *HF* | *Con* | *LC* | *HMU* | *HFL* |
| --- | --- | --- | --- | --- | --- | --- | --- | --- | --- | --- | --- | --- | --- | --- | --- | --- |
| *1^st^* | 0.41 | 0.02 | 0.48 | 0.09 | 0.00 | 0.00 | 0.00 | 0.00 | 0.00 | 0.00 | 0.00 | 0.00 | 0.00 | 0.00 | 0.00 | 0.00 |
| *2^nd^* | 0.23 | 0.18 | 0.40 | 0.18 | 0.00 | 0.00 | 0.00 | 0.00 | 0.00 | 0.00 | 0.00 | 0.00 | 0.00 | 0.00 | 0.00 | 0.00 |
| *3^rd^* | 0.15 | 0.46 | 0.10 | 0.26 | 0.00 | 0.01 | 0.01 | 0.00 | 0.00 | 0.00 | 0.00 | 0.00 | 0.00 | 0.00 | 0.00 | 0.00 |
| *4^th^* | 0.12 | 0.32 | 0.02 | 0.31 | 0.01 | 0.10 | 0.03 | 0.02 | 0.05 | 0.00 | 0.01 | 0.01 | 0.00 | 0.00 | 0.00 | 0.00 |
| *5^th^* | 0.03 | 0.02 | 0.00 | 0.08 | 0.06 | 0.30 | 0.09 | 0.12 | 0.25 | 0.00 | 0.01 | 0.03 | 0.00 | 0.00 | 0.01 | 0.00 |
| *6^th^* | 0.02 | 0.00 | 0.00 | 0.04 | 0.10 | 0.24 | 0.11 | 0.10 | 0.34 | 0.00 | 0.01 | 0.04 | 0.00 | 0.00 | 0.01 | 0.00 |
| *7^th^* | 0.01 | 0.00 | 0.00 | 0.03 | 0.17 | 0.18 | 0.15 | 0.13 | 0.23 | 0.01 | 0.02 | 0.06 | 0.00 | 0.00 | 0.01 | 0.00 |
| *8^th^* | 0.01 | 0.00 | 0.00 | 0.01 | 0.24 | 0.11 | 0.18 | 0.16 | 0.10 | 0.04 | 0.03 | 0.10 | 0.00 | 0.01 | 0.01 | 0.00 |
| *9^th^* | 0.00 | 0.00 | 0.00 | 0.01 | 0.22 | 0.05 | 0.17 | 0.17 | 0.03 | 0.13 | 0.04 | 0.13 | 0.00 | 0.02 | 0.02 | 0.01 |
| *10^th^* | 0.00 | 0.00 | 0.00 | 0.00 | 0.12 | 0.02 | 0.12 | 0.13 | 0.01 | 0.25 | 0.05 | 0.16 | 0.04 | 0.04 | 0.03 | 0.04 |
| *11^th^* | 0.00 | 0.00 | 0.00 | 0.00 | 0.04 | 0.01 | 0.07 | 0.08 | 0.00 | 0.25 | 0.07 | 0.15 | 0.16 | 0.06 | 0.04 | 0.09 |
| *12^th^* | 0.00 | 0.00 | 0.00 | 0.00 | 0.02 | 0.00 | 0.03 | 0.05 | 0.00 | 0.14 | 0.07 | 0.11 | 0.33 | 0.08 | 0.04 | 0.13 |
| *13^th^* | 0.00 | 0.00 | 0.00 | 0.00 | 0.01 | 0.00 | 0.02 | 0.02 | 0.00 | 0.09 | 0.09 | 0.09 | 0.24 | 0.14 | 0.05 | 0.25 |
| *14^th^* | 0.00 | 0.00 | 0.00 | 0.00 | 0.00 | 0.00 | 0.01 | 0.01 | 0.00 | 0.05 | 0.18 | 0.05 | 0.13 | 0.32 | 0.09 | 0.15 |
| *15^th^* | 0.00 | 0.00 | 0.00 | 0.00 | 0.00 | 0.00 | 0.00 | 0.01 | 0.00 | 0.03 | 0.35 | 0.04 | 0.08 | 0.18 | 0.17 | 0.14 |
| *16^th^* | 0.00 | 0.00 | 0.00 | 0.00 | 0.00 | 0.00 | 0.00 | 0.00 | 0.00 | 0.01 | 0.06 | 0.03 | 0.02 | 0.15 | 0.53 | 0.20 |
| *SUCRA* | 0.78 | 0.77 | 0.73 | 0.69 | 0.62 | 0.57 | 0.51 | 0.47 | 0.46 | 0.38 | 0.37 | 0.36 | 0.34 | 0.34 | 0.34 | 0.27 |

***2h POSTPRANDIAL GLUCOSE***

| *Rank* | *DAS* | *SE* | *LGY* | *ARE* | *LGI* | *HCC* | *RE* | *SOY* | *HFL* | *AE* | *CON* | *HC* | *HMU* | *LC* |
| --- | --- | --- | --- | --- | --- | --- | --- | --- | --- | --- | --- | --- | --- | --- |
| *1^st^* | 0.83 | 0.09 | 0.00 | 0.00 | 0.00 | 0.01 | 0.00 | 0.07 | 0.00 | 0.00 | 0.00 | 0.00 | 0.00 | 0.00 |
| *2^nd^* | 0.09 | 0.65 | 0.04 | 0.02 | 0.00 | 0.06 | 0.00 | 0.14 | 0.00 | 0.00 | 0.00 | 0.00 | 0.00 | 0.00 |
| *3^rd^* | 0.03 | 0.20 | 0.31 | 0.20 | 0.04 | 0.13 | 0.00 | 0.08 | 0.01 | 0.00 | 0.00 | 0.00 | 0.00 | 0.00 |
| *4^th^* | 0.02 | 0.05 | 0.29 | 0.33 | 0.10 | 0.11 | 0.01 | 0.05 | 0.03 | 0.00 | 0.00 | 0.01 | 0.01 | 0.00 |
| *5^th^* | 0.01 | 0.00 | 0.21 | 0.25 | 0.26 | 0.11 | 0.05 | 0.05 | 0.05 | 0.00 | 0.00 | 0.01 | 0.01 | 0.00 |
| *6^th^* | 0.01 | 0.00 | 0.10 | 0.14 | 0.23 | 0.13 | 0.21 | 0.05 | 0.10 | 0.00 | 0.00 | 0.01 | 0.01 | 0.00 |
| *7^th^* | 0.01 | 0.00 | 0.04 | 0.05 | 0.19 | 0.14 | 0.23 | 0.05 | 0.17 | 0.05 | 0.02 | 0.02 | 0.01 | 0.00 |
| *8^th^* | 0.01 | 0.00 | 0.01 | 0.01 | 0.11 | 0.10 | 0.26 | 0.05 | 0.22 | 0.11 | 0.08 | 0.02 | 0.02 | 0.00 |
| *9^th^* | 0.00 | 0.00 | 0.00 | 0.00 | 0.04 | 0.07 | 0.20 | 0.05 | 0.19 | 0.21 | 0.17 | 0.03 | 0.02 | 0.00 |
| *10^th^* | 0.00 | 0.00 | 0.00 | 0.00 | 0.01 | 0.07 | 0.02 | 0.04 | 0.11 | 0.33 | 0.34 | 0.04 | 0.03 | 0.01 |
| *11^th^* | 0.00 | 0.00 | 0.00 | 0.00 | 0.00 | 0.04 | 0.01 | 0.14 | 0.08 | 0.22 | 0.30 | 0.08 | 0.05 | 0.07 |
| *12^th^* | 0.00 | 0.00 | 0.00 | 0.00 | 0.00 | 0.02 | 0.00 | 0.06 | 0.02 | 0.05 | 0.06 | 0.26 | 0.15 | 0.38 |
| *13^th^* | 0.00 | 0.00 | 0.00 | 0.00 | 0.00 | 0.01 | 0.00 | 0.05 | 0.01 | 0.03 | 0.03 | 0.39 | 0.28 | 0.20 |
| *14^th^* | 0.00 | 0.00 | 0.00 | 0.00 | 0.00 | 0.00 | 0.00 | 0.11 | 0.00 | 0.00 | 0.00 | 0.12 | 0.42 | 0.33 |
| *SUCRA* | 0.96 | 0.91 | 0.75 | 0.73 | 0.62 | 0.60 | 0.51 | 0.50 | 0.45 | 0.32 | 0.3 | 0.16 | 0.11 | 0.09 |

***INSULIN REQUIREMENTS***

| *Rank* | *SE* | *DAS* | *RE* | *HFL* | *HC* | *AE* | *SOY* | *CON* | *LC* | *ER* | *LGI* | *HF* | *HCC* | *HGI* |
| --- | --- | --- | --- | --- | --- | --- | --- | --- | --- | --- | --- | --- | --- | --- |
| *1^st^* | 0.96 | 0.01 | 0.00 | 0.00 | 0.02 | 0.00 | 0.00 | 0.00 | 0.00 | 0.00 | 0.00 | 0.00 | 0.00 | 0.00 |
| *2^nd^* | 0.01 | 0.31 | 0.16 | 0.08 | 0.32 | 0.03 | 0.07 | 0.00 | 0.00 | 0.00 | 0.00 | 0.00 | 0.00 | 0.00 |
| *3^rd^* | 0.00 | 0.31 | 0.32 | 0.14 | 0.08 | 0.07 | 0.06 | 0.00 | 0.00 | 0.00 | 0.00 | 0.00 | 0.02 | 0.00 |
| *4^th^* | 0.00 | 0.21 | 0.29 | 0.22 | 0.07 | 0.12 | 0.05 | 0.00 | 0.00 | 0.02 | 0.00 | 0.00 | 0.01 | 0.00 |
| *5^th^* | 0.00 | 0.12 | 0.15 | 0.24 | 0.10 | 0.22 | 0.08 | 0.00 | 0.01 | 0.05 | 0.01 | 0.01 | 0.02 | 0.00 |
| *6^th^* | 0.00 | 0.04 | 0.05 | 0.16 | 0.09 | 0.26 | 0.11 | 0.04 | 0.05 | 0.11 | 0.05 | 0.02 | 0.03 | 0.00 |
| *7^th^* | 0.00 | 0.01 | 0.01 | 0.07 | 0.05 | 0.13 | 0.09 | 0.15 | 0.15 | 0.15 | 0.09 | 0.05 | 0.04 | 0.00 |
| *8^th^* | 0.00 | 0.00 | 0.00 | 0.03 | 0.03 | 0.07 | 0.04 | 0.25 | 0.21 | 0.12 | 0.12 | 0.07 | 0.04 | 0.00 |
| *9^th^* | 0.00 | 0.00 | 0.00 | 0.02 | 0.03 | 0.04 | 0.04 | 0.26 | 0.22 | 0.11 | 0.17 | 0.09 | 0.02 | 0.00 |
| *10^th^* | 0.00 | 0.00 | 0.00 | 0.01 | 0.03 | 0.03 | 0.06 | 0.18 | 0.17 | 0.11 | 0.23 | 0.15 | 0.03 | 0.01 |
| *11^th^* | 0.00 | 0.00 | 0.00 | 0.01 | 0.04 | 0.02 | 0.07 | 0.08 | 0.11 | 0.14 | 0.19 | 0.25 | 0.04 | 0.03 |
| *12^th^* | 0.00 | 0.00 | 0.00 | 0.01 | 0.05 | 0.01 | 0.16 | 0.02 | 0.05 | 0.10 | 0.11 | 0.23 | 0.07 | 0.20 |
| *13^th^* | 0.00 | 0.00 | 0.00 | 0.00 | 0.05 | 0.01 | 0.17 | 0.01 | 0.02 | 0.07 | 0.02 | 0.14 | 0.19 | 0.32 |
| *14^th^* | 0.00 | 0.00 | 0.00 | 0.00 | 0.05 | 0.00 | 0.00 | 0.00 | 0.00 | 0.02 | 0.00 | 0.00 | 0.48 | 0.44 |
| *SUCRA* | 0.99 | 0.82 | 0.80 | 0.70 | 0.64 | 0.62 | 0.43 | 0.40 | 0.39 | 0.38 | 0.34 | 0.26 | 0.15 | 0.07 |

***BIRTH WEIGHT***

| *Rank* | *DAS* | *HCC* | *LGI* | *HF* | *SOY* | *ARE* | *PMW* | *HFL* | *HMU* | *RE* | *HC* | *LC* | *SE* | *AE* | *CON* |
| --- | --- | --- | --- | --- | --- | --- | --- | --- | --- | --- | --- | --- | --- | --- | --- |
| *1^st^* | 0.94 | 0.00 | 0.00 | 0.01 | 0.00 | 0.00 | 0.02 | 0.00 | 0.03 | 0.00 | 0.00 | 0.00 | 0.00 | 0.00 | 0.00 |
| *2^nd^* | 0.06 | 0.24 | 0.04 | 0.19 | 0.10 | 0.03 | 0.16 | 0.00 | 0.14 | 0.00 | 0.04 | 0.01 | 0.00 | 0.00 | 0.00 |
| *3^rd^* | 0.00 | 0.25 | 0.13 | 0.14 | 0.12 | 0.07 | 0.10 | 0.01 | 0.07 | 0.01 | 0.08 | 0.01 | 0.00 | 0.00 | 0.00 |
| *4^th^* | 0.00 | 0.18 | 0.22 | 0.11 | 0.12 | 0.09 | 0.07 | 0.03 | 0.05 | 0.02 | 0.07 | 0.03 | 0.01 | 0.00 | 0.00 |
| *5^th^* | 0.00 | 0.12 | 0.22 | 0.09 | 0.11 | 0.11 | 0.06 | 0.06 | 0.04 | 0.04 | 0.06 | 0.04 | 0.02 | 0.00 | 0.00 |
| *6^th^* | 0.00 | 0.08 | 0.17 | 0.08 | 0.10 | 0.12 | 0.05 | 0.09 | 0.04 | 0.08 | 0.06 | 0.06 | 0.04 | 0.01 | 0.00 |
| *7^th^* | 0.00 | 0.05 | 0.11 | 0.07 | 0.09 | 0.12 | 0.05 | 0.12 | 0.04 | 0.12 | 0.06 | 0.08 | 0.06 | 0.02 | 0.00 |
| *8^th^* | 0.00 | 0.03 | 0.06 | 0.06 | 0.08 | 0.11 | 0.05 | 0.13 | 0.04 | 0.14 | 0.06 | 0.09 | 0.09 | 0.05 | 0.01 |
| *9^th^* | 0.00 | 0.02 | 0.03 | 0.05 | 0.07 | 0.09 | 0.04 | 0.12 | 0.04 | 0.15 | 0.07 | 0.10 | 0.10 | 0.08 | 0.04 |
| *10^th^* | 0.00 | 0.01 | 0.01 | 0.04 | 0.05 | 0.08 | 0.04 | 0.12 | 0.04 | 0.14 | 0.06 | 0.10 | 0.12 | 0.11 | 0.09 |
| *11^th^* | 0.00 | 0.00 | 0.00 | 0.04 | 0.04 | 0.06 | 0.04 | 0.09 | 0.03 | 0.12 | 0.05 | 0.09 | 0.12 | 0.14 | 0.17 |
| *12^th^* | 0.00 | 0.00 | 0.00 | 0.03 | 0.04 | 0.05 | 0.04 | 0.08 | 0.03 | 0.08 | 0.05 | 0.09 | 0.12 | 0.16 | 0.22 |
| *13^th^* | 0.00 | 0.00 | 0.00 | 0.03 | 0.03 | 0.03 | 0.05 | 0.06 | 0.04 | 0.06 | 0.08 | 0.13 | 0.11 | 0.16 | 0.23 |
| *14^th^* | 0.00 | 0.00 | 0.00 | 0.03 | 0.03 | 0.03 | 0.07 | 0.05 | 0.08 | 0.03 | 0.15 | 0.10 | 0.11 | 0.16 | 0.17 |
| *15^th^* | 0.00 | 0.00 | 0.00 | 0.04 | 0.02 | 0.02 | 0.16 | 0.03 | 0.28 | 0.01 | 0.10 | 0.07 | 0.09 | 0.11 | 0.07 |
| *SUCRA* | 0.99 | 0.78 | 0.70 | 0.63 | 0.60 | 0.54 | 0.50 | 0.43 | 0.43 | 0.42 | 0.42 | 0.35 | 0.30 | 0.22 | 0.20 |

***MACROSOMIA***

| *Rank* | *DAS* | *ARE* | *HC* | *LGI* | *RE* | *HFL* | *LC* | *SOY* | *PMW* | *SE* | *AE* | *CON* | *HF* |
| --- | --- | --- | --- | --- | --- | --- | --- | --- | --- | --- | --- | --- | --- |
| *1^st^* | 0.18 | 0.09 | 0.63 | 0.00 | 0.00 | 0.04 | 0.00 | 0.01 | 0.04 | 0.00 | 0.00 | 0.00 | 0.00 |
| *2^nd^* | 0.43 | 0.22 | 0.02 | 0.03 | 0.03 | 0.11 | 0.02 | 0.04 | 0.10 | 0.00 | 0.00 | 0.00 | 0.01 |
| *3^rd^* | 0.24 | 0.18 | 0.01 | 0.09 | 0.07 | 0.12 | 0.06 | 0.08 | 0.10 | 0.01 | 0.01 | 0.00 | 0.03 |
| *4^th^* | 0.09 | 0.12 | 0.01 | 0.15 | 0.13 | 0.10 | 0.11 | 0.12 | 0.09 | 0.04 | 0.03 | 0.00 | 0.03 |
| *5^th^* | 0.03 | 0.08 | 0.01 | 0.16 | 0.15 | 0.07 | 0.14 | 0.12 | 0.07 | 0.08 | 0.05 | 0.00 | 0.04 |
| *6^th^* | 0.01 | 0.06 | 0.00 | 0.15 | 0.14 | 0.06 | 0.14 | 0.11 | 0.06 | 0.13 | 0.07 | 0.01 | 0.04 |
| *7^th^* | 0.00 | 0.05 | 0.00 | 0.12 | 0.13 | 0.06 | 0.14 | 0.10 | 0.05 | 0.18 | 0.09 | 0.04 | 0.04 |
| *8^th^* | 0.00 | 0.04 | 0.00 | 0.10 | 0.11 | 0.05 | 0.12 | 0.09 | 0.05 | 0.19 | 0.10 | 0.10 | 0.04 |
| *9^th^* | 0.00 | 0.04 | 0.00 | 0.07 | 0.09 | 0.05 | 0.10 | 0.08 | 0.05 | 0.17 | 0.11 | 0.20 | 0.04 |
| *10^th^* | 0.00 | 0.03 | 0.00 | 0.06 | 0.07 | 0.06 | 0.07 | 0.08 | 0.06 | 0.12 | 0.13 | 0.26 | 0.06 |
| *11^th^* | 0.00 | 0.04 | 0.01 | 0.04 | 0.05 | 0.08 | 0.06 | 0.07 | 0.09 | 0.06 | 0.17 | 0.24 | 0.09 |
| *12^th^* | 0.00 | 0.03 | 0.01 | 0.02 | 0.03 | 0.12 | 0.03 | 0.06 | 0.14 | 0.02 | 0.16 | 0.13 | 0.23 |
| *13^th^* | 0.00 | 0.02 | 0.30 | 0.00 | 0.01 | 0.08 | 0.01 | 0.03 | 0.11 | 0.01 | 0.07 | 0.03 | 0.34 |
| *SUCRA* | 0.88 | 0.69 | 0.68 | 0.56 | 0.53 | 0.51 | 0.51 | 0.50 | 0.45 | 0.43 | 0.31 | 0.25 | 0.21 |

***PRETERM BIRTH***

| *Rank* | *AE* | *HFL* | *SE* | *LG* | *HF* | *CON* | *SOY* | *RE* |
| --- | --- | --- | --- | --- | --- | --- | --- | --- |
| *1^st^* | 0.17 | 0.65 | 0.04 | 0.02 | 0.09 | 0.00 | 0.03 | 0.00 |
| *2^nd^* | 0.42 | 0.01 | 0.16 | 0.10 | 0.22 | 0.01 | 0.08 | 0.00 |
| *3^rd^* | 0.21 | 0.01 | 0.27 | 0.23 | 0.12 | 0.08 | 0.06 | 0.02 |
| *4^th^* | 0.09 | 0.00 | 0.24 | 0.28 | 0.08 | 0.23 | 0.05 | 0.03 |
| *5^th^* | 0.06 | 0.00 | 0.16 | 0.21 | 0.08 | 0.34 | 0.05 | 0.08 |
| *6^th^* | 0.04 | 0.01 | 0.10 | 0.11 | 0.14 | 0.26 | 0.10 | 0.25 |
| *7^th^* | 0.01 | 0.02 | 0.04 | 0.03 | 0.16 | 0.07 | 0.26 | 0.41 |
| *8^th^* | 0.00 | 0.29 | 0.01 | 0.00 | 0.10 | 0.01 | 0.37 | 0.22 |
| *SUCRA* | 0.77 | 0.68 | 0.60 | 0.56 | 0.52 | 0.43 | 0.26 | 0.19 |

***Supplementary S10: Cumulative Rank Curve***

***FASTING PLASMA GLUCOSE***


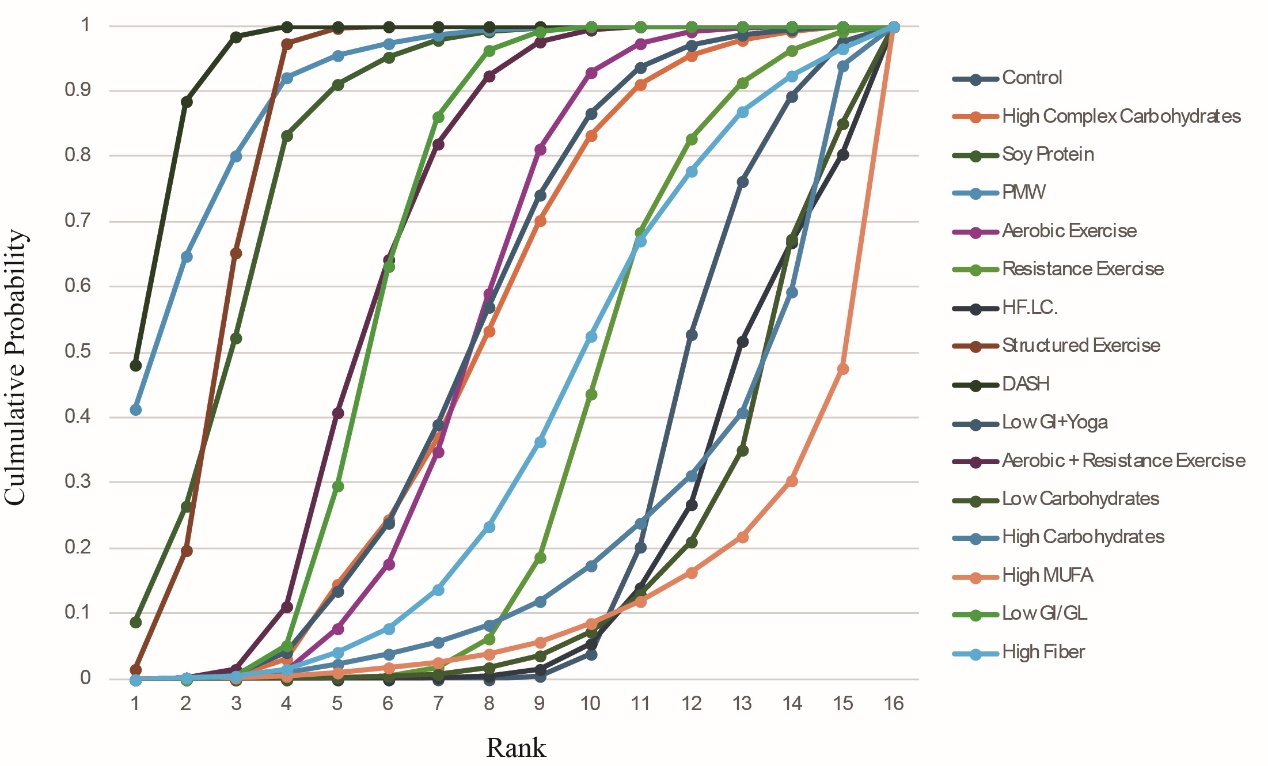


***2h POSTPRANDIAL GLUCOSE***

***
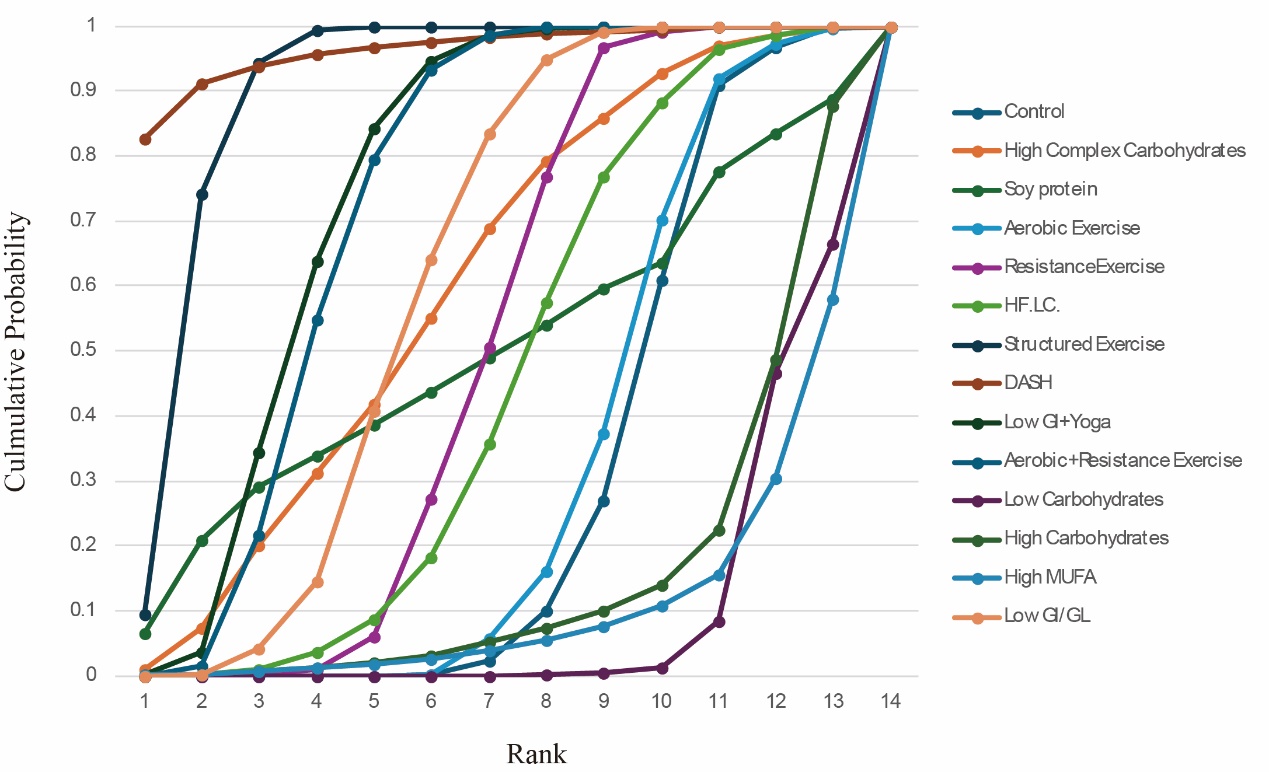
***

***INSULIN REQUIREMENTS***

***
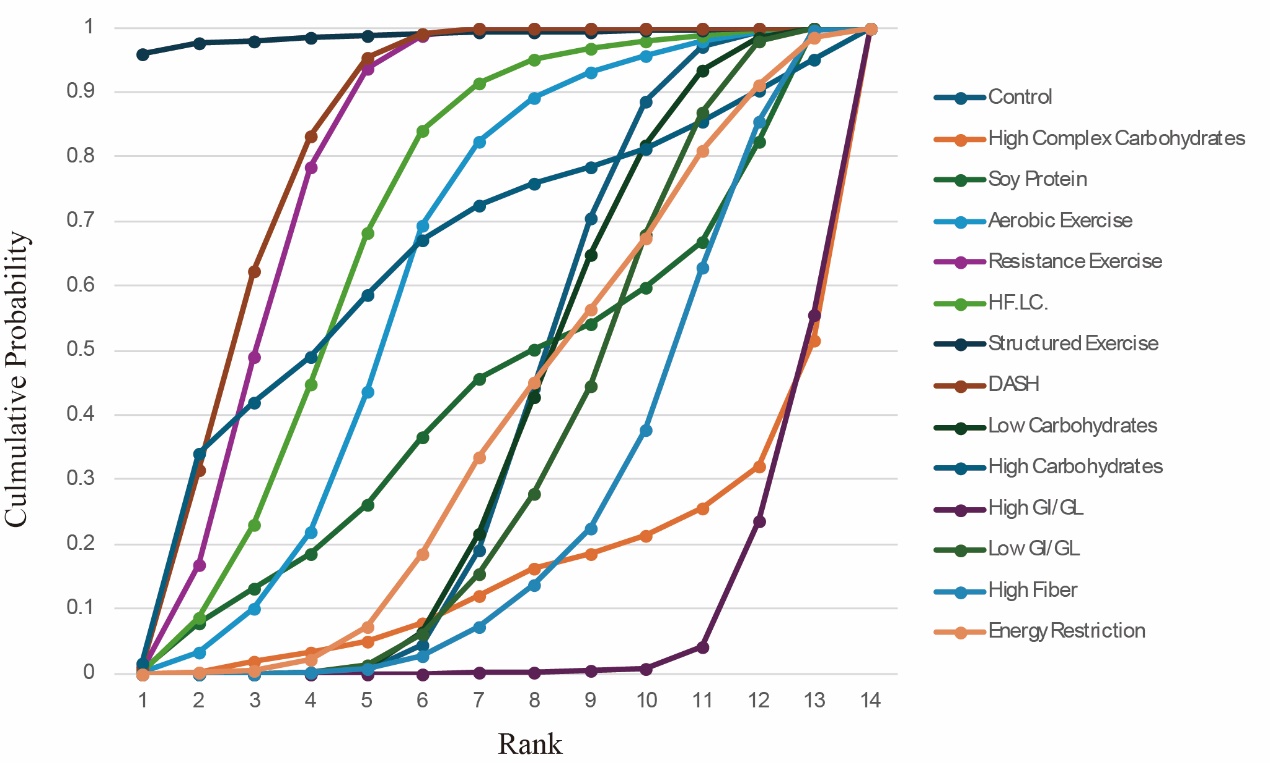
***

***BIRTH WEIGHT***

***
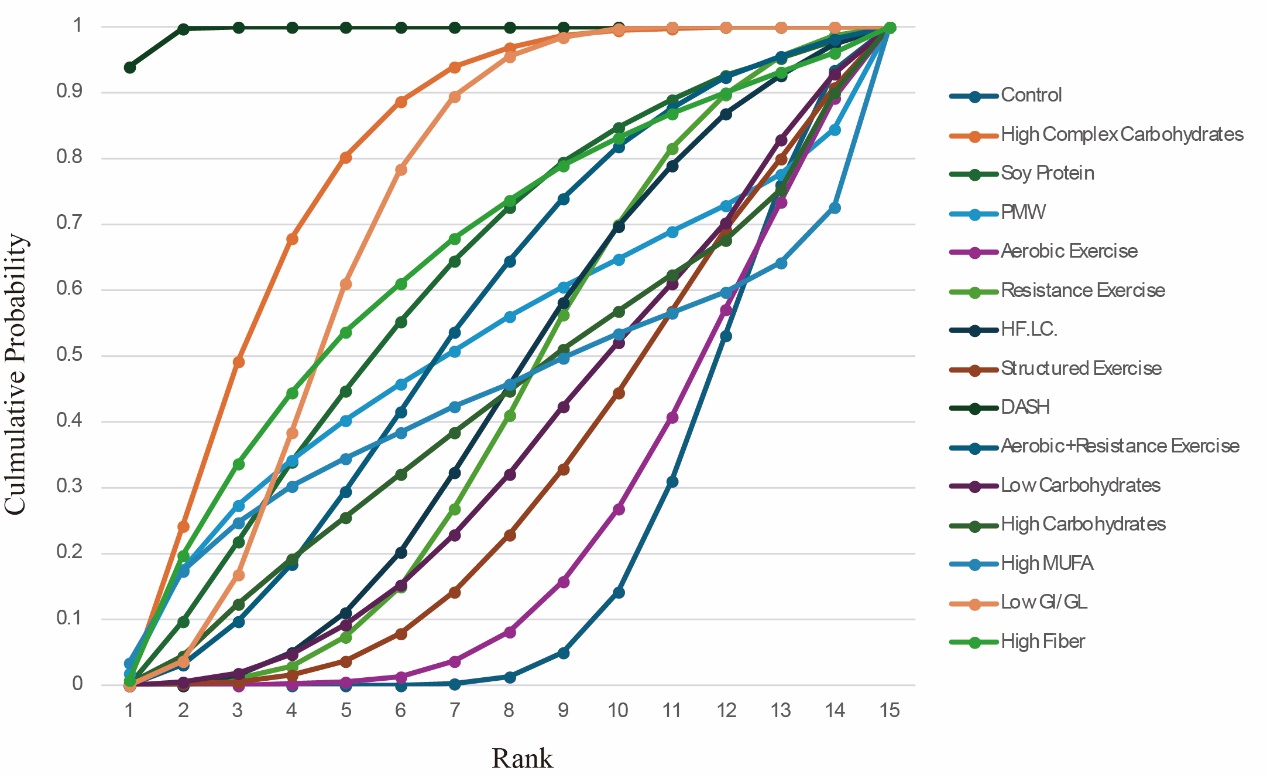
***

***MACROSOMIA***

***
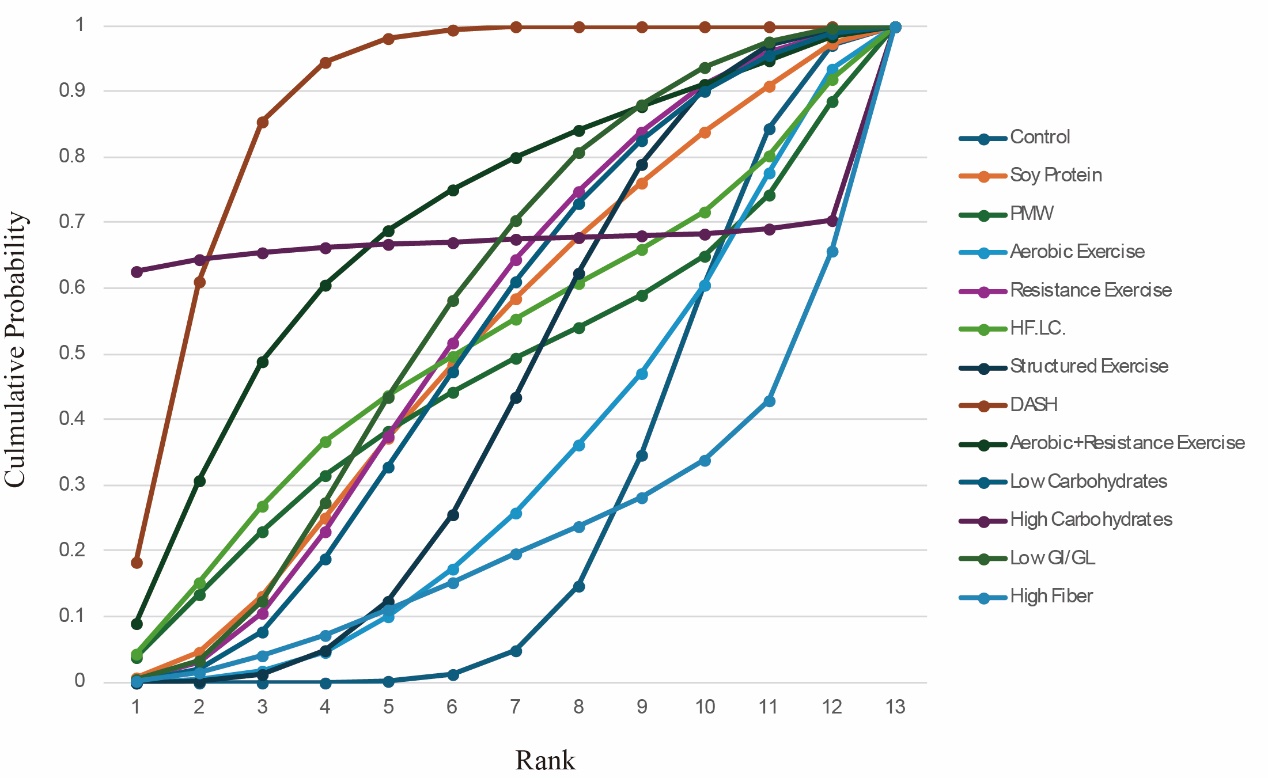
***

***PRETERM BIRTH***

***
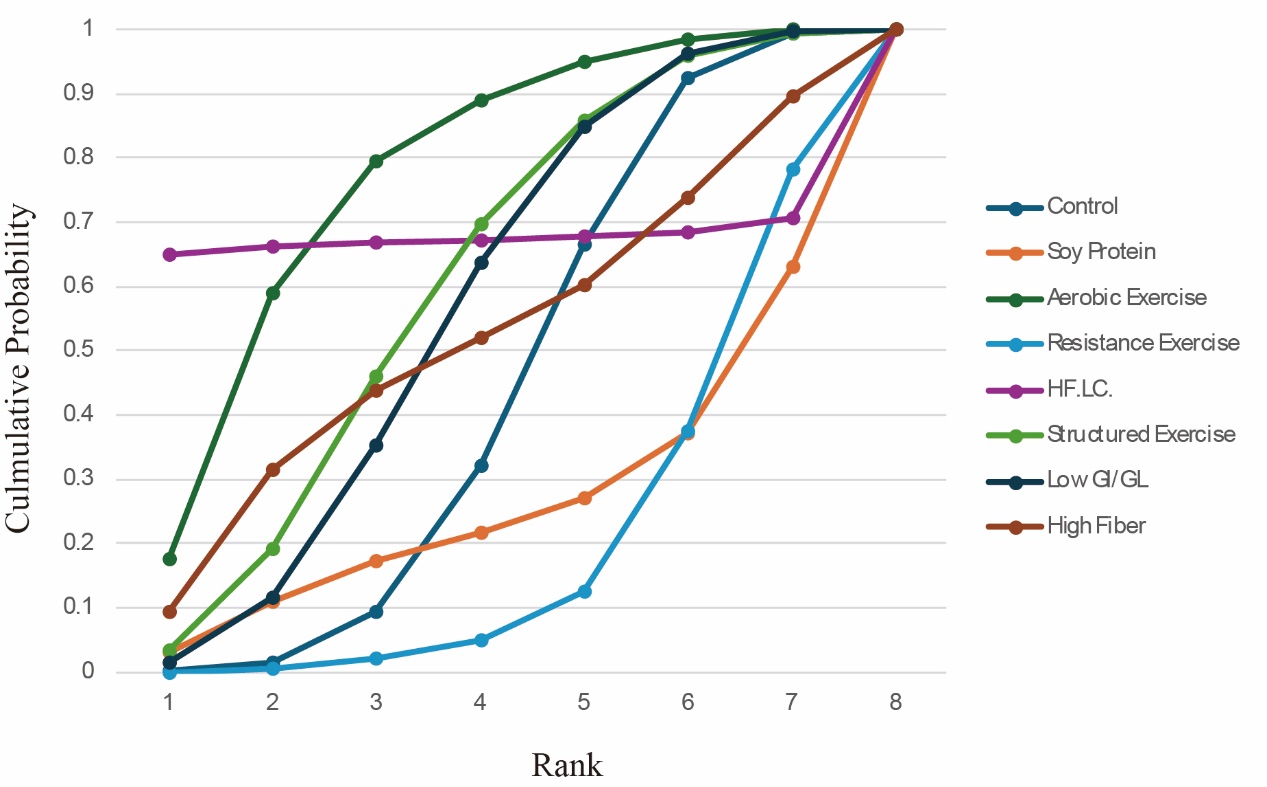
***

***Supplementary S11: The Grading of Recommendations, Assessment, Development, and Evaluation (GRADE)***

*AE: Aerobic Exercise; ARE: Aerobic + Resistance Exercise; CON: Control; ER: Energy Restriction; HC: High Carbohydrates; HCC: High Complex Carbohydrates; HF: High Fiber; HFL: Higher fat/Lower Carbohydrates; HGI: High GI/GL; HMU: High MUFA (Monounsaturated Fatty Acid); LC: Low Carbohydrates; LGI: Low GI/GL; LGY: Low GI + Yoga; PMW: Postmeal Walking; RE: Resistance Exercise; SE: Structured Exercise; Soy: Soy Protein;*

*The table below shows the rating of downgrading for each comparison under the factors of limitation, indirectness, inconsistency, imprecision, and publication bias, where 1 represents serious and 2 represents very serious downgrading, and null indicates no downgrading. In the GRADE item, 4 represents high quality evidence, 3 moderate quality, 2 low quality, and -1,0, and 1 very low quality evidence.*

***FASTING PLASMA GLUCOSE***

| ***Comparison*** | ***Study Number*** | ***Limitation*** | ***Indirection*** | ***Inconsistency*** | ***Imprecision*** | ***Publication bias*** | ***Comparison Type*** | ***GRADE*** |
| --- | --- | --- | --- | --- | --- | --- | --- | --- |
| CON vs DASH | 2 | 2 |  |  | 1 |  | Direct | 1 |
| CON vs LC | 1 | 2 |  |  | 1 |  | Direct | 1 |
| CON vs LGI | 4 | 2 |  | 1 | 1 | 1 | Direct | -1 |
| CON vs SOY | 1 | 2 |  | 1 | 1 |  | Direct | 0 |
| CON vs AE | 1 | 2 |  | 1 | 1 |  | Direct | 0 |
| CON vs RE | 1 | 2 |  |  | 1 |  | Direct | 1 |
| CON vs HFL | 1 | 2 |  | 1 | 1 |  | Direct | 0 |
| CON vs SE | 4 | 2 |  | 1 |  |  | Direct | 1 |
| CON vs LGY | 1 | 2 |  |  | 1 |  | Direct | 1 |
| CON vs ARE | 1 | 2 |  |  | 1 |  | Direct | 1 |
| LC vs HC | 1 | 2 |  |  | 1 |  | Direct | 1 |
| HMU vs HC | 1 | 1 |  |  | 1 |  | Direct | 2 |
| LGI vs HF | 1 | 0 |  |  | 1 |  | Direct | 3 |
| HCC vs SOY | 1 | 2 |  | 1 | 1 |  | Direct | 0 |
| HCC vs HFL | 3 | 2 |  |  | 1 |  | Direct | 1 |
| PMW vs AE | 1 | 2 |  |  | 1 |  | Direct | 1 |
| AE vs RE | 1 | 2 |  | 1 | 1 | 1 | Direct | -1 |
| AE vs HFL | 1 | 2 |  | 1 | 1 |  | Direct | 0 |
| RE vs HFL | 1 | 2 |  | 1 | 1 |  | Direct | 0 |
| CON vs HC |  | 2 |  |  | 1 |  | Indirect | 1 |
| CON vs HMU |  | 2 |  |  | 1 |  | Indirect | 1 |
| CON vs HF |  | 2 |  |  | 1 |  | Indirect | 1 |
| CON vs HCC |  | 2 |  |  | 1 |  | Indirect | 1 |
| CON vs PMW |  | 1 |  |  | 1 |  | Indirect | 2 |
| DASH vs LC |  | 2 |  |  | 1 |  | Indirect | 1 |
| DASH vs HC |  | 2 |  |  | 1 |  | Indirect | 1 |
| DASH vs HMU |  | 2 |  |  | 1 |  | Indirect | 1 |
| DASH vs LGI |  | 2 |  |  | 1 |  | Indirect | 1 |
| DASH vs HF |  | 2 |  |  | 1 |  | Indirect | 1 |
| DASH vs HCC |  | 2 |  |  | 1 |  | Indirect | 1 |
| DASH vs SOY |  | 2 |  |  | 1 |  | Indirect | 1 |
| DASH vs PMW |  | 2 |  |  | 1 |  | Indirect | 1 |
| DASH vs AE |  | 2 |  |  | 1 |  | Indirect | 1 |
| DASH vs RE |  | 2 |  |  | 1 |  | Indirect | 1 |
| DASH vs HFL |  | 2 |  |  | 1 |  | Indirect | 1 |
| DASH vs SE |  | 2 |  |  | 1 |  | Indirect | 1 |
| DASH vs LGY |  | 2 |  |  | 1 |  | Indirect | 1 |
| DASH vs ARE |  | 1 | 1 |  | 1 |  | Indirect | 1 |
| LC vs HMU |  | 2 |  |  | 1 |  | Indirect | 1 |
| LC vs LGI |  | 2 |  |  | 1 |  | Indirect | 1 |
| LC vs HF |  | 2 |  |  | 1 |  | Indirect | 1 |
| LC vs HCC |  | 2 |  |  | 1 |  | Indirect | 1 |
| LC vs SOY |  | 2 |  |  | 1 |  | Indirect | 1 |
| LC vs PMW |  | 2 |  |  | 1 |  | Indirect | 1 |
| LC vs AE |  | 2 |  |  | 1 |  | Indirect | 1 |
| LC vs RE |  | 2 |  |  | 1 |  | Indirect | 1 |
| LC vs HFL |  | 2 |  |  | 1 |  | Indirect | 1 |
| LC vs SE |  | 2 |  |  | 1 |  | Indirect | 1 |
| LC vs LGY |  | 2 |  |  | 1 |  | Indirect | 1 |
| LC vs ARE |  | 1 | 1 |  | 1 |  | Indirect | 1 |
| HC vs LGI |  | 2 |  |  | 1 |  | Indirect | 1 |
| HC vs HF |  | 2 |  |  | 1 |  | Indirect | 1 |
| HC vs HCC |  | 2 |  |  | 1 |  | Indirect | 1 |
| HC vs SOY |  | 2 |  |  | 1 |  | Indirect | 1 |
| HC vs PMW |  | 2 |  |  | 1 |  | Indirect | 1 |
| HC vs AE |  | 2 |  |  | 1 |  | Indirect | 1 |
| HC vs RE |  | 2 |  |  | 1 |  | Indirect | 1 |
| HC vs HFL |  | 2 |  |  | 1 |  | Indirect | 1 |
| HC vs SE |  | 2 |  |  | 1 |  | Indirect | 1 |
| HC vs LGY |  | 2 |  |  | 1 |  | Indirect | 1 |
| HC vs ARE |  | 1 | 1 |  | 1 |  | Indirect | 1 |
| HMU vs HF |  | 2 |  |  | 1 |  | Indirect | 1 |
| LGI vs HMU |  | 2 |  |  | 1 |  | Indirect | 1 |
| LGI vs HCC |  | 2 |  |  | 1 |  | Indirect | 1 |
| LGI vs SOY |  | 2 |  |  | 1 |  | Indirect | 1 |
| LGI vs PMW |  | 2 |  |  | 1 |  | Indirect | 1 |
| LGI vs AE |  | 2 |  |  | 1 |  | Indirect | 1 |
| LGI vs RE |  | 2 |  |  | 1 |  | Indirect | 1 |
| LGI vs HFL |  | 2 |  |  | 1 |  | Indirect | 1 |
| LGI vs SE |  | 2 |  |  | 1 |  | Indirect | 1 |
| LGI vs LGY |  | 2 |  |  | 1 |  | Indirect | 1 |
| LGI vs ARE |  | 2 | 1 |  | 1 |  | Indirect | 0 |
| HCC vs HMU |  | 2 |  |  | 1 |  | Indirect | 1 |
| HCC vs HF |  | 2 |  |  | 1 |  | Indirect | 1 |
| HCC vs PMW |  | 2 |  |  | 1 |  | Indirect | 1 |
| HCC vs AE |  | 2 |  |  | 1 |  | Indirect | 1 |
| HCC vs RE |  | 2 |  |  | 1 |  | Indirect | 1 |
| HCC vs HFL |  | 2 |  |  | 1 |  | Indirect | 1 |
| HCC vs SE |  | 2 |  |  | 1 |  | Indirect | 1 |
| HCC vs LGY |  | 2 |  |  | 1 |  | Indirect | 1 |
| HCC vs ARE |  | 2 | 1 |  | 1 |  | Indirect | 0 |
| SOY vs HMU |  | 2 |  |  | 1 |  | Indirect | 1 |
| SOY vs HF |  | 2 |  |  | 1 |  | Indirect | 1 |
| SOY vs PMW |  | 2 |  |  | 1 |  | Indirect | 1 |
| SOY vs AE |  | 2 |  |  | 1 |  | Indirect | 1 |
| SOY vs RE |  | 2 |  |  | 1 |  | Indirect | 1 |
| SOY vs HFL |  | 2 |  |  | 1 |  | Indirect | 1 |
| SOY vs SE |  | 2 |  |  | 1 |  | Indirect | 1 |
| SOY vs LGY |  | 1 |  |  | 1 |  | Indirect | 2 |
| SOY vs ARE |  | 2 | 1 |  | 1 |  | Indirect | 0 |
| PMW vs HMU |  | 2 |  |  | 1 |  | Indirect | 1 |
| PMW vs HF |  | 2 |  |  | 1 |  | Indirect | 1 |
| PMW vs RE |  | 2 |  |  | 1 |  | Indirect | 1 |
| PMW vs HFL |  | 2 |  |  | 1 |  | Indirect | 1 |
| PMW vs SE |  | 2 |  |  | 1 |  | Indirect | 1 |
| PMW vs LGY |  | 2 |  |  | 1 |  | Indirect | 1 |
| PMW vs ARE |  | 2 | 1 |  | 1 |  | Indirect | 0 |
| AE vs HMU |  | 2 |  |  | 1 |  | Indirect | 1 |
| AE vs HF |  | 2 |  |  | 1 |  | Indirect | 1 |
| AE vs SE |  | 2 |  |  | 1 |  | Indirect | 1 |
| AE vs LGY |  | 1 |  |  | 1 |  | Indirect | 2 |
| AE vs ARE |  | 2 | 1 |  | 1 |  | Indirect | 0 |
| RE vs HMU |  | 2 |  |  | 1 |  | Indirect | 1 |
| RE vs HF |  | 2 |  |  | 1 |  | Indirect | 1 |
| RE vs SE |  | 2 |  |  | 1 |  | Indirect | 1 |
| RE vs LGY |  | 1 |  |  | 1 |  | Indirect | 2 |
| RE vs ARE |  | 2 | 1 | 1 | 1 |  | Indirect | -1 |
| HFL vs HMU |  | 2 |  |  | 1 |  | Indirect | 1 |
| HFL vs HF |  | 2 |  |  | 1 |  | Indirect | 1 |
| HFL vs SE |  | 2 |  |  | 1 |  | Indirect | 1 |
| HFL vs LGY |  | 1 |  |  | 1 |  | Indirect | 2 |
| HFL vs ARE |  | 2 | 1 |  | 1 |  | Indirect | 0 |
| SE vs HMU |  | 2 |  |  | 1 |  | Indirect | 1 |
| SE vs HF |  | 2 |  |  | 1 |  | Indirect | 1 |
| SE vs LGY |  | 1 |  |  | 1 |  | Indirect | 2 |
| SE vs AR |  | 2 | 1 |  | 1 |  | Indirect | 0 |
| LGY vs HMU |  | 2 |  |  | 1 |  | Indirect | 1 |
| LGY vs HF |  | 1 |  |  | 1 |  | Indirect | 2 |
| LGY vs ARE |  | 2 | 1 |  | 1 |  | Indirect | 0 |
| AR vs HMU |  | 1 | 1 |  | 1 |  | Indirect | 1 |
| AR vs HF |  | 2 | 1 |  | 1 |  | Indirect | 0 |
| Rank |  | 2 |  |  | 1 |  |  | 1 |

***2h POSTPRANDIAL GLUCOSE***

| ***Comparison*** | ***Study Number*** | ***Limitation*** | ***Indirection*** | ***Inconsistency*** | ***Imprecision*** | ***Publication bias*** | ***Comparison Type*** | ***GRADE*** |
| --- | --- | --- | --- | --- | --- | --- | --- | --- |
| CON vs DASH | 1 | 2 |  | 1 |  |  | Direct | 1 |
| CON vs LC | 1 | 2 |  |  |  |  | Direct | 2 |
| CON vs LGI | 4 | 2 |  |  |  |  | Direct | 2 |
| CON vs AE | 1 | 2 |  |  | 1 |  | Direct | 1 |
| CON vs RE | 1 | 2 |  |  |  |  | Direct | 2 |
| CON vs HFL | 1 | 2 |  | 1 | 1 |  | Direct | 0 |
| CON vs SE | 3 | 2 |  | 1 |  | 1 | Direct | 0 |
| CON vs LGY | 1 | 2 |  | 1 |  |  | Direct | 1 |
| CON vs ARE | 1 | 2 |  | 1 |  |  | Direct | 1 |
| LC vs HC | 1 | 2 |  |  | 1 |  | Direct | 1 |
| HMU vs HC | 1 | 1 |  |  | 1 |  | Direct | 2 |
| HCC vs SOY | 1 | 2 |  |  | 1 |  | Direct | 1 |
| HCC vs HFL | 1 | 2 |  |  | 1 |  | Direct | 1 |
| AE vs RE | 1 | 2 |  |  |  |  | Direct | 2 |
| RE vs HFL | 1 | 2 |  | 1 | 1 |  | Direct | 0 |
| CON vs HC |  | 2 |  |  | 1 |  | Indirect | 1 |
| CON vs HMU |  | 2 |  | 1 | 1 |  | Indirect | 0 |
| CON vs HCC |  | 2 |  |  | 1 |  | Indirect | 1 |
| CON vs SOY |  | 2 |  |  | 1 |  | Indirect | 1 |
| DASH vs HMU |  | 2 |  | 1 |  |  | Indirect | 1 |
| DASH vs LC |  | 2 |  | 1 |  |  | Indirect | 1 |
| LC vs HMU |  | 2 |  |  | 1 |  | Indirect | 1 |
| LC vs LGI |  | 2 |  | 1 |  |  | Indirect | 1 |
| LC vs HCC |  | 2 |  |  |  |  | Indirect | 2 |
| LC vs SOY |  | 2 |  |  | 1 |  | Indirect | 1 |
| LC vs AE |  | 2 |  |  |  |  | Indirect | 2 |
| LC vs RE |  | 2 |  | 1 |  |  | Indirect | 1 |
| LC vs HFL |  | 2 |  |  |  |  | Indirect | 2 |
| LC vs SE |  | 2 |  |  |  |  | Indirect | 2 |
| LC vs LGY |  | 2 |  | 1 |  |  | Indirect | 1 |
| LC vs ARE |  | 2 | 1 | 1 |  |  | Indirect | 0 |
| HC vs DASH |  | 2 |  | 1 |  |  | Indirect | 1 |
| HC vs LGI |  | 2 |  |  | 1 |  | Indirect | 1 |
| HC vs HCC |  | 2 |  |  | 1 |  | Indirect | 1 |
| HC vs SOY |  | 2 |  |  | 1 |  | Indirect | 1 |
| HC vs AE |  | 2 |  |  | 1 |  | Indirect | 1 |
| HC vs RE |  | 2 |  |  | 1 |  | Indirect | 1 |
| HC vs HFL |  | 2 |  |  | 1 |  | Indirect | 1 |
| HC vs SE |  | 2 |  | 1 |  |  | Indirect | 1 |
| HC vs LGY |  | 2 |  |  |  |  | Indirect | 2 |
| HC vs ARE |  | 2 | 1 |  |  |  | Indirect | 1 |
| DASH vs LGI |  | 2 |  |  | 1 |  | Indirect | 1 |
| LGI vs HMU |  | 2 |  |  | 1 |  | Indirect | 1 |
| LGI vs HCC |  | 2 |  |  | 1 |  | Indirect | 1 |
| LGI vs SOY |  | 2 |  |  | 1 |  | Indirect | 1 |
| LGI vs AE |  | 2 |  |  |  |  | Indirect | 2 |
| LGI vs RE |  | 2 |  |  | 1 |  | Indirect | 1 |
| LGI vs HFL |  | 2 |  |  | 1 |  | Indirect | 1 |
| LGI vs SE |  | 2 |  | 1 |  |  | Indirect | 1 |
| LGI vs LGY |  | 2 |  |  | 1 |  | Indirect | 1 |
| LGI vs ARE |  | 2 | 1 |  | 1 |  | Indirect | 0 |
| HCC vs DASH |  | 2 |  |  | 1 |  | Indirect | 1 |
| HCC vs HMU |  | 2 |  |  | 1 |  | Indirect | 1 |
| HCC vs AE |  | 2 |  |  | 1 |  | Indirect | 1 |
| HCC vs RE |  | 2 |  |  | 1 |  | Indirect | 1 |
| HCC vs SE |  | 2 |  |  | 1 |  | Indirect | 1 |
| HCC vs LGY |  | 2 |  |  | 1 |  | Indirect | 1 |
| HCC vs ARE |  | 2 | 1 |  | 1 |  | Indirect | 0 |
| SOY vs DASH |  | 2 |  |  | 1 |  | Indirect | 1 |
| SOY vs HMU |  | 2 |  |  | 1 |  | Indirect | 1 |
| SOY vs AE |  | 2 |  |  | 1 |  | Indirect | 1 |
| SOY vs RE |  | 2 |  |  | 1 |  | Indirect | 1 |
| SOY vs HFL |  | 2 |  |  | 1 |  | Indirect | 1 |
| SOY vs SE |  | 2 |  |  | 1 |  | Indirect | 1 |
| SOY vs LGY |  | 2 |  |  | 1 |  | Indirect | 1 |
| SOY vs ARE |  | 2 | 1 |  | 1 |  | Indirect | 0 |
| DASH vs AE |  | 2 |  | 1 |  |  | Indirect | 1 |
| AE vs HMU |  | 2 |  |  | 1 |  | Indirect | 1 |
| AE vs HFL |  | 2 |  |  | 1 |  | Indirect | 1 |
| AE vs SE |  | 2 |  | 1 |  |  | Indirect | 1 |
| AE vs LGY |  | 2 |  |  |  |  | Indirect | 2 |
| AE vs ARE |  | 2 | 1 |  |  |  | Indirect | 1 |
| DASH vs RE |  | 2 |  |  |  |  | Indirect | 2 |
| RE vs HMU |  | 2 |  |  | 1 |  | Indirect | 1 |
| RE vs SE |  | 2 |  | 1 |  |  | Indirect | 1 |
| RE vs LGY |  | 2 |  |  |  |  | Indirect | 2 |
| RE vs ARE |  | 2 | 1 |  |  |  | Indirect | 1 |
| DASH vs HFL |  | 2 |  |  |  |  | Indirect | 2 |
| HFL vs HMU |  | 2 |  |  | 1 |  | Indirect | 1 |
| HFL vs SE |  | 2 |  | 1 |  |  | Indirect | 1 |
| HFL vs LGY |  | 2 |  |  | 1 |  | Indirect | 1 |
| HFL vs ARE |  | 2 | 1 |  | 1 |  | Indirect | 0 |
| DASH vs SE |  | 2 |  |  | 1 |  | Indirect | 1 |
| SE vs HMU |  | 2 |  |  |  |  | Indirect | 2 |
| SE vs LGY |  | 2 |  |  |  |  | Indirect | 2 |
| SE vs ARE |  | 2 | 1 |  |  |  | Indirect | 1 |
| DASH vs LGY |  | 2 |  |  | 1 |  | Indirect | 1 |
| LGY vs HMU |  | 2 |  |  |  |  | Indirect | 2 |
| LGY vs ARE |  | 2 | 1 |  | 1 |  | Indirect | 0 |
| DASH vs ARE |  | 2 | 1 |  | 1 |  | Indirect | 0 |
| AR vs HMU |  | 2 | 1 |  |  |  | Indirect | 1 |
| Rank |  | 2 |  |  | 1 |  |  | 1 |

***INSULIN REQUIREMENTS***

| ***Comparison*** | ***Study Number*** | ***Limitation*** | ***Indirection*** | ***Inconsistency*** | ***Imprecision*** | ***Publication bias*** | ***Comparison Type*** | ***GRADE*** |
| --- | --- | --- | --- | --- | --- | --- | --- | --- |
| CON vs DASH | 3 | 2 |  |  | 0 |  | Direct | 2 |
| CON vs LC | 3 | 2 |  |  | 0 |  | Direct | 2 |
| CON vs LGI | 1 | 2 |  |  | 0 |  | Direct | 2 |
| CON vs ER | 1 | 1 |  | 1 | 0 |  | Direct | 2 |
| CON vs SOY | 1 | 2 |  |  | 1 |  | Direct | 1 |
| CON vs AE | 3 | 2 |  | 1 | 1 |  | Direct | 0 |
| CON vs RE | 2 | 2 |  | 1 | 0 |  | Direct | 1 |
| CON vs SE | 1 | 2 |  |  | 0 |  | Direct | 2 |
| LC vs HC | 1 | 2 |  |  | 1 |  | Direct | 1 |
| HGI vs LGI | 2 | 0 |  |  | 0 |  | Direct | 4 |
| LGI vs HF | 1 | 0 |  |  | 1 |  | Direct | 3 |
| HCC vs SOY | 1 | 2 |  |  | 0 |  | Direct | 2 |
| AE vs RE | 1 | 2 |  |  | 1 |  | Direct | 1 |
| RE vs HFL | 1 | 2 |  |  | 1 |  | Direct | 1 |
| CON vs HC |  | 1 |  |  | 1 |  | Indirect | 2 |
| CON vs HGI |  | 2 |  |  | 0 |  | Indirect | 2 |
| CON vs HF |  | 2 |  |  | 0 |  | Indirect | 2 |
| CON vs HCC |  | 1 |  |  | 1 |  | Indirect | 2 |
| CON vs HFL |  | 2 |  |  | 1 |  | Indirect | 1 |
| DASH vs LC |  | 2 |  |  | 0 |  | Indirect | 2 |
| DASH vs HC |  | 7 |  |  | 1 |  | Indirect | -4 |
| DASH vs HGI |  | 2 |  |  | 0 |  | Indirect | 2 |
| DASH vs LGI |  | 2 |  | 1 | 0 |  | Indirect | 1 |
| DASH vs HF |  | 2 |  | 1 | 0 |  | Indirect | 1 |
| DASH vs ER |  | 2 |  |  | 0 |  | Indirect | 2 |
| DASH vs HCC |  | 2 |  | 1 | 1 |  | Indirect | 0 |
| DASH vs SOY |  | 2 |  |  | 1 |  | Indirect | 1 |
| DASH vs AE |  | 2 |  |  | 1 |  | Indirect | 1 |
| DASH vs RE |  | 2 |  |  | 1 |  | Indirect | 1 |
| DASH vs HFL |  | 1 |  |  | 1 |  | Indirect | 2 |
| DASH vs SE |  | 2 |  |  | 0 |  | Indirect | 2 |
| LC vs HGI |  | 1 |  |  | 0 |  | Indirect | 3 |
| LC vs LGI |  | 2 |  |  | 1 |  | Indirect | 1 |
| LC vs HF |  | 2 |  |  | 1 |  | Indirect | 1 |
| LC vs ER |  | 2 |  |  | 1 |  | Indirect | 1 |
| LC vs HCC |  | 2 |  |  | 1 |  | Indirect | 1 |
| LC vs SOY |  | 2 |  |  | 1 |  | Indirect | 1 |
| LC vs AE |  | 2 |  |  | 1 |  | Indirect | 1 |
| LC vs RE |  | 2 |  |  | 0 |  | Indirect | 2 |
| LC vs HFL |  | 1 |  | 1 | 1 |  | Indirect | 1 |
| LC vs SE |  | 2 |  |  | 0 |  | Indirect | 2 |
| HC vs HF |  | 1 |  |  | 1 |  | Indirect | 2 |
| HC vs HFL |  | 2 |  |  | 1 |  | Indirect | 1 |
| HGI vs HC |  | 1 |  |  | 1 |  | Indirect | 2 |
| HGI vs HF |  | 1 |  |  | 0 |  | Indirect | 3 |
| HGI vs ER |  | 1 |  |  | 1 |  | Indirect | 2 |
| HGI vs HCC |  | 1 |  |  | 1 |  | Indirect | 2 |
| HGI vs SOY |  | 2 |  | 1 | 1 |  | Indirect | 0 |
| HGI vs AE |  | 1 |  |  | 0 |  | Indirect | 3 |
| HGI vs RE |  | 2 |  |  | 0 |  | Indirect | 2 |
| HGI vs HFL |  | 2 |  |  | 0 |  | Indirect | 2 |
| HGI vs SE |  | 2 |  |  | 0 |  | Indirect | 2 |
| LGI vs HC |  | 2 |  |  | 1 |  | Indirect | 1 |
| LGI vs ER |  | 2 |  |  | 1 |  | Indirect | 1 |
| LGI vs HCC |  | 2 |  |  | 1 |  | Indirect | 1 |
| LGI vs SOY |  | 2 |  |  | 1 |  | Indirect | 1 |
| LGI vs AE |  | 2 |  | 1 | 1 |  | Indirect | 0 |
| LGI vs RE |  | 2 |  |  | 1 |  | Indirect | 1 |
| LGI vs HFL |  | 2 |  | 1 | 1 |  | Indirect | 0 |
| LGI vs SE |  | 2 |  |  | 0 |  | Indirect | 2 |
| HF vs HFL |  | 2 |  | 1 | 1 |  | Indirect | 0 |
| ER vs HC |  | 2 |  |  | 1 |  | Indirect | 1 |
| ER vs HF |  | 2 |  |  | 1 |  | Indirect | 1 |
| ER vs HCC |  | 2 |  |  | 1 |  | Indirect | 1 |
| ER vs SOY |  | 1 |  |  | 1 |  | Indirect | 2 |
| ER vs AE |  | 2 |  | 1 | 1 |  | Indirect | 0 |
| ER vs RE |  | 2 |  |  | 0 |  | Indirect | 2 |
| ER vs HFL |  | 2 |  | 1 | 1 |  | Indirect | 0 |
| ER vs SE |  | 2 |  |  | 0 |  | Indirect | 2 |
| HCC vs HC |  | 2 |  |  | 1 |  | Indirect | 1 |
| HCC vs HF |  | 2 |  |  | 1 |  | Indirect | 1 |
| HCC vs AE |  | 2 |  | 1 | 1 |  | Indirect | 0 |
| HCC vs RE |  | 2 |  |  | 1 |  | Indirect | 1 |
| HCC vs HFL |  | 2 |  |  | 1 |  | Indirect | 1 |
| HCC vs SE |  | 2 |  | 1 | 0 |  | Indirect | 1 |
| SOY vs HC |  | 2 |  |  | 1 |  | Indirect | 1 |
| SOY vs HF |  | 0 |  |  | 1 |  | Indirect | 3 |
| SOY vs AE |  | 2 |  |  | 1 |  | Indirect | 1 |
| SOY vs RE |  | 2 |  | 1 | 1 |  | Indirect | 0 |
| SOY vs HFL |  | 2 |  |  | 1 |  | Indirect | 1 |
| SOY vs SE |  | 1 |  |  | 0 |  | Indirect | 3 |
| AE vs HC |  | 2 |  |  | 1 |  | Indirect | 1 |
| AE vs HF |  | 2 |  | 1 | 1 |  | Indirect | 0 |
| AE vs HFL |  | 2 |  |  | 1 |  | Indirect | 1 |
| AE vs SE |  | 1 |  |  | 0 |  | Indirect | 3 |
| RE vs HC |  | 2 |  |  | 1 |  | Indirect | 1 |
| RE vs HF |  | 2 |  |  | 0 |  | Indirect | 2 |
| RE vs SE |  | 2 |  |  | 1 |  | Indirect | 1 |
| SE vs HC |  | 2 |  |  | 0 |  | Indirect | 2 |
| SE vs HF |  | 2 |  |  | 0 |  | Indirect | 2 |
| SE vs HFL |  | 2 |  |  | 0 |  | Indirect | 2 |
| Rank |  | 2 |  |  | 1 |  |  | 1 |

***BIRTH WEIGHT***

| ***Comparison*** | ***Study Number*** | ***Limitation*** | ***Indirection*** | ***Inconsistency*** | ***Imprecision*** | ***Publication bias*** | ***Comparison Type*** | ***GRADE*** |
| --- | --- | --- | --- | --- | --- | --- | --- | --- |
| CON vs DASH | 3 | 2 |  |  | 0 |  | Direct | 2 |
| CON vs LC | 2 | 2 |  |  | 1 |  | Direct | 1 |
| CON vs LGI | 3 | 2 |  |  | 0 |  | Direct | 2 |
| CON vs SOY | 1 | 2 |  |  | 1 |  | Direct | 1 |
| CON vs AE | 3 | 2 |  |  | 1 |  | Direct | 1 |
| CON vs RE | 2 | 2 |  |  | 1 |  | Direct | 1 |
| CON vs HFL | 1 | 2 |  | 1 | 1 |  | Direct | 0 |
| CON vs SE | 2 | 2 |  |  | 1 |  | Direct | 1 |
| CON vs ARE | 1 | 2 |  |  | 1 |  | Direct | 1 |
| LC vs HC | 1 | 2 |  |  | 1 |  | Direct | 1 |
| HMU vs HC | 1 | 2 |  |  | 1 |  | Direct | 1 |
| LGI vs HF | 1 | 2 |  |  | 1 |  | Direct | 1 |
| HCC vs SOY | 1 | 2 |  |  | 1 |  | Direct | 1 |
| HCC vs HFL | 3 | 2 |  | 1 | 0 |  | Direct | 1 |
| PMW vs AE | 1 | 2 |  |  | 1 |  | Direct | 4 |
| AE vs RE | 1 | 2 |  |  | 1 |  | Direct | 4 |
| AE vs HFL | 1 | 2 |  | 1 | 1 |  | Direct | 0 |
| CON vs HC |  | 2 |  |  | 1 |  | Indirect | 1 |
| CON vs HMU |  | 2 |  | 1 | 1 |  | Indirect | 0 |
| CON vs HF |  | 2 |  |  | 1 |  | Indirect | 1 |
| CON vs HCC |  | 2 |  |  | 1 |  | Indirect | 1 |
| CON vs PMW |  | 2 |  |  | 1 |  | Indirect | 1 |
| DASH vs LC |  | 2 |  |  | 0 |  | Indirect | 2 |
| DASH vs HC |  | 2 |  |  | 0 |  | Indirect | 2 |
| DASH vs HMU |  | 2 |  |  | 1 |  | Indirect | 1 |
| DASH vs LGI |  | 2 |  |  | 0 |  | Indirect | 2 |
| DASH vs HF |  | 2 |  |  | 0 |  | Indirect | 2 |
| DASH vs HCC |  | 2 |  |  | 0 |  | Indirect | 2 |
| DASH vs SOY |  | 2 |  |  | 0 |  | Indirect | 2 |
| DASH vs PMW |  | 2 |  |  | 0 |  | Indirect | 2 |
| DASH vs AE |  | 2 |  |  | 0 |  | Indirect | 2 |
| DASH vs RE |  | 2 |  |  | 0 |  | Indirect | 2 |
| DASH vs HFL |  | 2 |  |  | 0 |  | Indirect | 2 |
| DASH vs SE |  | 2 |  |  | 0 |  | Indirect | 2 |
| DASH vs ARE |  | 1 | 1 |  | 0 |  | Indirect | 2 |
| LC vs HMU |  | 2 |  |  | 1 |  | Indirect | 1 |
| LC vs LGI |  | 2 |  |  | 1 |  | Indirect | 1 |
| LC vs HF |  | 2 |  |  | 1 |  | Indirect | 1 |
| LC vs HCC |  | 2 |  |  | 1 |  | Indirect | 1 |
| LC vs SOY |  | 2 |  |  | 1 |  | Indirect | 1 |
| LC vs PMW |  | 2 |  |  | 1 |  | Indirect | 1 |
| LC vs AE |  | 2 |  |  | 1 |  | Indirect | 1 |
| LC vs RE |  | 2 |  |  | 1 |  | Indirect | 1 |
| LC vs HFL |  | 2 |  |  | 1 |  | Indirect | 1 |
| LC vs SE |  | 2 |  |  | 1 |  | Indirect | 1 |
| LC vs ARE |  | 1 | 1 |  | 1 |  | Indirect | 1 |
| HC vs LGI |  | 2 |  |  | 1 |  | Indirect | 1 |
| HC vs HF |  | 2 |  |  | 1 |  | Indirect | 1 |
| HC vs HCC |  | 2 |  |  | 1 |  | Indirect | 1 |
| HC vs SOY |  | 2 |  |  | 1 |  | Indirect | 1 |
| HC vs PMW |  | 2 |  |  | 1 |  | Indirect | 1 |
| HC vs AE |  | 2 |  |  | 1 |  | Indirect | 1 |
| HC vs RE |  | 2 |  |  | 1 |  | Indirect | 1 |
| HC vs HFL |  | 2 |  |  | 1 |  | Indirect | 1 |
| HC vs SE |  | 2 |  |  | 1 |  | Indirect | 1 |
| HC vs ARE |  | 2 | 1 |  | 1 |  | Indirect | 0 |
| HMU vs HF |  | 2 |  |  | 1 |  | Indirect | 1 |
| LGI vs HMU |  | 2 |  |  | 1 |  | Indirect | 1 |
| LGI vs HCC |  | 2 |  |  | 1 |  | Indirect | 1 |
| LGI vs SOY |  | 2 |  |  | 1 |  | Indirect | 1 |
| LGI vs PMW |  | 2 |  |  | 1 |  | Indirect | 1 |
| LGI vs AE |  | 2 |  |  | 0 |  | Indirect | 2 |
| LGI vs RE |  | 2 |  |  | 1 |  | Indirect | 1 |
| LGI vs HFL |  | 2 |  |  | 1 |  | Indirect | 1 |
| LGI vs SE |  | 2 |  |  | 1 |  | Indirect | 1 |
| LGI vs ARE |  | 2 | 1 |  | 1 |  | Indirect | 0 |
| HCC vs HMU |  | 2 |  |  | 1 |  | Indirect | 1 |
| HCC vs HF |  | 2 |  |  | 1 |  | Indirect | 1 |
| HCC vs PMW |  | 2 |  |  | 1 |  | Indirect | 1 |
| HCC vs AE |  | 2 |  |  | 0 |  | Indirect | 2 |
| HCC vs RE |  | 2 |  | 1 | 1 |  | Indirect | 0 |
| HCC vs SE |  | 2 |  |  | 1 |  | Indirect | 1 |
| HCC vs ARE |  | 2 | 1 |  | 1 |  | Indirect | 0 |
| SOY vs HMU |  | 2 |  |  | 1 |  | Indirect | 1 |
| SOY vs HF |  | 2 |  |  | 1 |  | Indirect | 1 |
| SOY vs PMW |  | 2 |  |  | 1 |  | Indirect | 1 |
| SOY vs AE |  | 2 |  |  | 1 |  | Indirect | 1 |
| SOY vs RE |  | 2 |  |  | 1 |  | Indirect | 1 |
| SOY vs HFL |  | 2 |  |  | 1 |  | Indirect | 1 |
| SOY vs SE |  | 1 |  |  | 1 |  | Indirect | 2 |
| SOY vs ARE |  | 2 | 1 |  | 1 |  | Indirect | 0 |
| PMW vs HMU |  | 2 |  | 1 | 1 |  | Indirect | 0 |
| PMW vs HF |  | 2 |  |  | 1 |  | Indirect | 1 |
| PMW vs RE |  | 2 |  |  | 1 |  | Indirect | 1 |
| PMW vs HFL |  | 1 |  |  | 1 |  | Indirect | 2 |
| PMW vs SE |  | 1 |  |  | 1 |  | Indirect | 2 |
| PMW vs ARE |  | 2 | 1 |  | 1 |  | Indirect | 0 |
| AE vs HMU |  | 2 |  |  | 1 |  | Indirect | 1 |
| AE vs HF |  | 2 |  |  | 1 |  | Indirect | 1 |
| AE vs SE |  | 2 |  |  | 1 |  | Indirect | 1 |
| AE vs ARE |  | 2 | 1 |  | 1 |  | Indirect | 0 |
| RE vs HMU |  | 2 |  | 1 | 1 |  | Indirect | 0 |
| RE vs HF |  | 2 |  |  | 1 |  | Indirect | 1 |
| RE vs HFL |  | 2 |  |  | 1 |  | Indirect | 1 |
| RE vs SE |  | 2 |  |  | 1 |  | Indirect | 1 |
| RE vs ARE |  | 2 | 1 |  | 1 |  | Indirect | 0 |
| HFL vs HMU |  | 2 |  |  | 1 |  | Indirect | 1 |
| HFL vs HF |  | 2 |  |  | 1 |  | Indirect | 1 |
| HFL vs SE |  | 2 |  |  | 1 |  | Indirect | 1 |
| HFL vs ARE |  | 2 | 1 |  | 1 |  | Indirect | 0 |
| SE vs HMU |  | 2 |  |  | 1 |  | Indirect | 1 |
| SE vs HF |  | 2 |  |  | 1 |  | Indirect | 1 |
| SE vs ARE |  | 2 | 1 |  | 1 |  | Indirect | 0 |
| ARE vs HMU |  | 1 | 1 |  | 1 |  | Indirect | 1 |
| ARE vs HF |  | 1 | 1 |  | 1 |  | Indirect | 1 |
| Rank |  | 2 |  |  | 1 |  |  | 1 |

***MACROSOMIA***

| ***Comparison*** | ***Study Number*** | ***Limitation*** | ***Indirection*** | ***Inconsistency*** | ***Imprecision*** | ***Publication bias*** | ***Comparison Type*** | ***GRADE*** |
| --- | --- | --- | --- | --- | --- | --- | --- | --- |
| CON vs DASH | 2 | 2 |  |  | 0 |  | Direct | 2 |
| CON vs LC | 3 | 2 |  |  | 1 |  | Direct | 1 |
| CON vs LGI | 4 | 2 |  |  | 1 |  | Direct | 1 |
| CON vs SOY | 1 | 2 |  |  | 1 |  | Direct | 1 |
| CON vs AE | 1 | 2 |  |  | 1 |  | Direct | 1 |
| CON vs RE | 2 | 2 |  |  | 1 |  | Direct | 1 |
| CON vs HFL | 1 | 2 |  |  | 1 |  | Direct | 1 |
| CON vs SE | 3 | 2 |  | 1 | 1 |  | Direct | 0 |
| CON vs ARE | 1 | 2 |  |  | 1 |  | Direct | 1 |
| LC vs HC | 1 | 2 |  |  | 1 |  | Direct | 1 |
| LGI vs HF | 1 | 0 |  |  | 1 |  | Direct | 3 |
| PMW vs AE | 1 | 2 |  |  | 1 |  | Direct | 1 |
| AE vs RE | 1 | 2 |  | 1 | 1 |  | Direct | 0 |
| CON vs HC |  | 1 |  |  | 1 |  | Indirect | 2 |
| CON vs HF |  | 2 |  | 1 | 1 |  | Indirect | 0 |
| CON vs PMW |  | 2 |  |  | 1 |  | Indirect | 1 |
| DASH vs LC |  | 2 |  |  | 0 |  | Indirect | 2 |
| DASH vs HC |  | 2 |  |  | 1 |  | Indirect | 1 |
| DASH vs LGI |  | 2 |  |  | 1 |  | Indirect | 1 |
| DASH vs HF |  | 1 |  |  | 0 |  | Indirect | 3 |
| DASH vs SOY |  | 2 |  |  | 1 |  | Indirect | 1 |
| DASH vs PMW |  | 2 |  |  | 1 |  | Indirect | 1 |
| DASH vs AE |  | 2 |  |  | 0 |  | Indirect | 2 |
| DASH vs RE |  | 2 |  |  | 1 |  | Indirect | 1 |
| DASH vs HFL |  | 2 |  |  | 1 |  | Indirect | 1 |
| DASH vs SE |  | 2 |  |  | 0 |  | Indirect | 2 |
| DASH vs ARE |  | 2 | 1 |  | 1 |  | Indirect | 0 |
| LC vs LGI |  | 2 |  |  | 1 |  | Indirect | 1 |
| LC vs HF |  | 2 |  |  | 1 |  | Indirect | 1 |
| LC vs SOY |  | 1 |  |  | 1 |  | Indirect | 2 |
| LC vs PMW |  | 2 |  |  | 1 |  | Indirect | 1 |
| LC vs AE |  | 2 |  |  | 1 |  | Indirect | 1 |
| LC vs RE |  | 2 |  |  | 1 |  | Indirect | 1 |
| LC vs HFL |  | 2 |  |  | 1 |  | Indirect | 1 |
| LC vs SE |  | 2 |  |  | 1 |  | Indirect | 1 |
| LC vs ARE |  | 2 | 1 |  | 1 |  | Indirect | 0 |
| HC vs HF |  | 2 |  |  | 1 |  | Indirect | 1 |
| LGI vs HCC |  | 1 |  |  | 1 |  | Indirect | 2 |
| LGI vs SOY |  | 2 |  |  | 1 |  | Indirect | 1 |
| LGI vs PMW |  | 2 |  |  | 1 |  | Indirect | 1 |
| LGI vs AE |  | 2 |  |  | 1 |  | Indirect | 1 |
| LGI vs RE |  | 2 |  |  | 1 |  | Indirect | 1 |
| LGI vs HFL |  | 2 |  |  | 1 |  | Indirect | 1 |
| LGI vs SE |  | 2 |  |  | 1 |  | Indirect | 1 |
| LGI vs ARE |  | 1 | 1 |  | 1 |  | Indirect | 1 |
| SOY vs HC |  | 2 |  |  | 1 |  | Indirect | 1 |
| SOY vs HF |  | 2 |  |  | 1 |  | Indirect | 1 |
| SOY vs PMW |  | 2 |  |  | 1 |  | Indirect | 1 |
| SOY vs AE |  | 2 |  |  | 1 |  | Indirect | 1 |
| SOY vs RE |  | 2 |  |  | 1 |  | Indirect | 1 |
| SOY vs HFL |  | 2 |  |  | 1 |  | Indirect | 1 |
| SOY vs SE |  | 2 |  |  | 1 |  | Indirect | 1 |
| SOY vs ARE |  | 1 | 1 |  | 1 |  | Indirect | 1 |
| PMW vs HC |  | 1 |  |  | 1 |  | Indirect | 2 |
| PMW vs HF |  | 1 |  |  | 1 |  | Indirect | 2 |
| PMW vs RE |  | 1 |  |  | 1 |  | Indirect | 2 |
| PMW vs HFL |  | 2 |  |  | 1 |  | Indirect | 1 |
| PMW vs SE |  | 2 |  |  | 1 |  | Indirect | 1 |
| PMW vs ARE |  | 2 | 1 |  | 1 |  | Indirect | 0 |
| AE vs HC |  | 2 |  |  | 1 |  | Indirect | 1 |
| AE vs HF |  | 2 |  |  | 1 |  | Indirect | 1 |
| AE vs HFL |  | 2 |  |  | 1 |  | Indirect | 1 |
| AE vs SE |  | 2 |  |  | 1 |  | Indirect | 1 |
| AE vs ARE |  | 2 | 1 |  | 1 |  | Indirect | 0 |
| RE vs HC |  | 2 |  |  | 1 |  | Indirect | 1 |
| RE vs HF |  | 2 |  |  | 1 |  | Indirect | 1 |
| RE vs HFL |  | 2 |  |  | 1 |  | Indirect | 1 |
| RE vs SE |  | 2 |  |  | 1 |  | Indirect | 1 |
| RE vs ARE |  | 2 | 1 |  | 1 |  | Indirect | 0 |
| HFL vs HC |  | 2 |  |  | 1 |  | Indirect | 1 |
| HFL vs HF |  | 2 |  |  | 1 |  | Indirect | 1 |
| HFL vs SE |  | 2 |  |  | 1 |  | Indirect | 1 |
| HFL vs ARE |  | 2 | 1 |  | 1 |  | Indirect | 0 |
| SE vs HC |  | 2 |  |  | 1 |  | Indirect | 1 |
| SE vs HF |  | 2 |  |  | 1 |  | Indirect | 1 |
| SE vs ARE |  | 2 | 1 |  | 1 |  | Indirect | 0 |
| ARE vs HC |  | 2 | 1 |  | 1 |  | Indirect | 0 |
| ARE vs HF |  | 2 | 1 |  | 1 |  | Indirect | 0 |
| Rank |  | 2 |  |  | 1 | 1 |  | 0 |

***PRETERM BIRTH***

| ***Comparison*** | ***Study Number*** | ***Limitation*** | ***Indirection*** | ***Inconsistency*** | ***Imprecision*** | ***Publication bias*** | ***Comparison Type*** | ***GRADE*** |
| --- | --- | --- | --- | --- | --- | --- | --- | --- |
| CON vs LGI | 3 | 2 |  | 1 | 1 |  | ***Direct*** | 0 |
| CON vs SOY | 1 | 2 |  |  | 1 |  | ***Direct*** | 1 |
| CON vs AE | 2 | 2 |  | 1 | 1 |  | ***Direct*** | 0 |
| CON vs RE | 2 | 2 |  |  | 1 |  | ***Direct*** | 1 |
| CON vs HFL | 1 | 2 |  |  | 1 |  | ***Direct*** | 1 |
| CON vs SE | 4 | 2 |  | 1 | 1 |  | ***Direct*** | 0 |
| LG vs HF | 1 | 0 |  | 1 | 1 |  | ***Direct*** | 2 |
| AE vs RE | 1 | 2 |  |  | 0 |  | ***Direct*** | 2 |
| CON vs HF |  | 1 |  |  | 1 |  | ***Indirect*** | 2 |
| LG vs SOY |  | 2 |  |  | 1 |  | ***Indirect*** | 1 |
| LGI vs AE |  | 2 |  |  | 1 |  | ***Indirect*** | 1 |
| LGI vs RE |  | 2 |  |  | 1 |  | ***Indirect*** | 1 |
| LGI vs HFL |  | 2 |  |  | 1 |  | ***Indirect*** | 1 |
| LGI vs SE |  | 2 |  |  | 1 |  | ***Indirect*** | 1 |
| SOY vs HF |  | 2 |  |  | 1 |  | ***Indirect*** | 1 |
| SOY vs AE |  | 2 |  |  | 1 |  | ***Indirect*** | 1 |
| SOY vs RE |  | 2 |  |  | 1 |  | ***Indirect*** | 1 |
| SOY vs HFL |  | 2 |  |  | 1 |  | ***Indirect*** | 1 |
| SOY vs SE |  | 1 |  |  | 1 |  | ***Indirect*** | 2 |
| AE vs HF |  | 2 |  |  | 1 |  | ***Indirect*** | 1 |
| AE vs HFL |  | 2 |  |  | 1 |  | ***Indirect*** | 1 |
| AE vs SE |  | 1 |  |  | 1 |  | ***Indirect*** | 2 |
| RE vs HF |  | 2 |  |  | 1 |  | ***Indirect*** | 1 |
| RE vs HFL |  | 2 |  |  | 1 |  | ***Indirect*** | 1 |
| RE vs SE |  | 1 |  | 1 | 1 |  | ***Indirect*** | 1 |
| HFL vs HF |  | 2 |  |  | 1 |  | ***Indirect*** | 1 |
| HFL vs SE |  | 1 |  |  | 1 |  | ***Indirect*** | 2 |
| SE vs HF |  | 1 |  |  | 1 |  | ***Indirect*** | 2 |
| Rank |  | 2 |  |  |  |  |  | 2 |

***Supplementary S12: League Tables for Sensitive Analysis***

*AE: Aerobic Exercise; ARE: Aerobic + Resistance Exercise; ER: Energy Restriction; High Carb: High Carbohydrates; HCC: High Complex Carbohydrates; HFLC: Higher Fiber/Lower Carbohydrates; Low Carb: Low Carbohydrates; LGY: Low GI + Yoga; PMW: Postmeal Walking; RE: Resistance Exercise; SE: Structured Exercise; Soy:Soy Protein;*

***FASTING PLASMA GLUCOSE (Big Loop)***

| ***Dash*** |  | | | | | | | | | | |
| --- | --- | --- | --- | --- | --- | --- | --- | --- | --- | --- | --- |
| -13.32  (-19.29, -7.42) | ***Low Carb*** |  | | | | | | | | | |
| -13.37  (-21.81, -4.86) | -0.03  (-5.94, 5.98) | ***High Carb*** |  | | | | | | | | |
| -15.12  (-24.18, -5.97) | -1.78  (-8.69, 5.14) | -1.79  (-5.38, 1.81) | ***High MUFA*** |  |  |  |  |  |  |  |  |
| -14.17  (-21.83, -6.53) | -0.82  (-9.94, 8.24) | -0.84  (-11.74, 9.96) | 0.96  (-10.46, 12.45) | ***HCC*** |  |  |  |  |  |  |  |
| -9.55  (-14.18, -4.81) | 3.81  (-2.85, 10.63) | 3.83  (-5.09, 12.87) | 5.56  (-3.99, 15.26) | 4.67  (-3.65, 12.85) | ***RE*** |  | |  | |  | |
| -17.12  (-23.99, -10.29) | -3.78  (-12.21, 4.53) | -3.76  (-14.1, 6.44) | -1.99  (-12.85, 8.83) | -2.96  (-6.46, 0.47) | -7.61  (-15.12, -0.03) | ***HFL*** |  |  |  |  |  |
| -7.67  (-12.17, -3.14) | 5.69  (-0.92, 12.3) | 5.68  (-3.29, 14.57) | 7.5  (-2.06, 17) | 6.5  (-1.57, 14.77) | 1.85  (-3.62, 7.4) | 9.45  (2.15, 16.96) | ***LGY*** | |  |  |  |
| -5.96  (-9.46, -2.5) | 7.39  (1.39, 13.37) | 7.41  (-1.03, 15.86) | 9.15  (0.11, 18.36) | 8.23  (0.55, 16) | 3.57  (-1.1, 8.27) | 11.19  (4.3, 18.08) | 1.74  (-2.82, 6.19) | | ***ARE*** | |  |
| -11.52  (-14.01, -9.07) | 1.8  (-3.62, 7.25) | 1.79  (-6.32, 9.95) | 3.56  (-5.25, 12.28) | 2.66  (-4.61, 9.97) | -1.99  (-5.95, 1.98) | 5.59  (-0.79, 11.97) | -3.85  (-7.64, -0.13) | | -5.58  (-8.02, -3.09) | | ***Control*** |

***2h POSTPRANDIAL GLUCOSE***

***(Big Loop)***

| ***DASH*** |  | |  |  |  |  |  |  |  |
| --- | --- | --- | --- | --- | --- | --- | --- | --- | --- |
| -34.27  (-56.3, -12.97) | ***Low Carb*** |  |  |  |  |  |  |  |  |
| -33.74  (-59.44, -8.68) | 0.63  (-12.54, 14.15) | ***High Carb*** |  |  |  |  |  |  |  |
| -35.46  (-61.96, -9.62) | -1.13  (-16.13, 13.71) | -1.84  (-8.07, 4.52) | ***High MUFA*** |  |  |  |  |  |  |
| -19.11  (-40, 1.54) | 15.29  (6.95, 23.42) | 14.62  (-1.08, 30.52) | 16.45  (-0.59, 33.44) | ***Low GI/GL*** |  |  |  |  |  |
| -25.42  (-46.32, -4.82) | 8.94  (0.9, 16.93) | 8.32  (-7.36, 24) | 10.13  (-6.81, 27.19) | -6.31  (-11.12, -1.51) | ***AE*** |  |  |  |  |
| -22.09  (-42.97, -1.51) | 12.3  (4.26, 20.31) | 11.66  (-4.11, 27.3) | 13.47  (-3.57, 30.55) | -3  (-7.85, 1.84) | 3.32  (0.58, 6.08) | ***RE*** |  |  |  |
| -16.44  (-37.26, 3.93) | 17.87  (9.79, 25.86) | 17.33  (1.55, 32.81) | 19.1  (2.26, 35.93) | 2.61  (-2.14, 7.42) | 8.95  (4.59, 13.34) | 5.63  (1.21, 10.11) | ***LGY*** |  |  |
| -16.88  (-37.67, 3.41) | 17.44  (9.52, 25.25) | 16.78  (1.22, 32.17) | 18.6  (1.65, 35.33) | 2.15  (-2.26, 6.68) | 8.48  (4.37, 12.57) | 5.13  (0.96, 9.33) | -0.48  (-4.5, 3.58) | ***ARE*** |  |
| -25.4  (-46.08, -5.15) | 8.96  (1.57, 16.35) | 8.3  (-7.06, 23.58) | 10.12  (-6.73, 26.84) | -6.3  (-9.96, -2.65) | 0.01  (-3.11, 3.11) | -3.31  (-6.51, -0.07) | -8.93  (-11.97, -5.85) | -8.45  (-11.1, -5.83) | ***Control*** |

***2h POSTPRANDIAL GLUCOSE***

***(Small Loop)***

| ***HCC*** |  |  |
| --- | --- | --- |
| -1.26  (-21.95, 19.79) | ***Soy*** |  |
| -3.01  (-11.26, 5.27) | -1.71  (-24.14, 20.47) | ***HFLC*** |

***INSULIN REQUIREMENTS***

***Network plot after removing zero event***

| ***DASH*** |  |  |  |  |  |  |  |  |  |  |  |  |
| --- | --- | --- | --- | --- | --- | --- | --- | --- | --- | --- | --- | --- |
| 0.29  (0.15, 0.5) | ***Low carb*** |  |  |  |  |  |  |  |  |  |  |  |
| 0.69  (0.05, 23.55) | 2.36  (0.21, 77.29) | ***High carb*** |  |  |  |  |  |  |  |  |  |  |
| 0.11  (0.04, 0.27) | 0.37  (0.16, 0.84) | 0.15  (0, 2.03) | ***High GI/GL*** |  |  |  |  |  |  |  |  |  |
| 0.26  (0.11, 0.53) | 0.89  (0.48, 1.61) | 0.37  (0.01, 4.56) | 2.38  (1.4, 4.46) | ***Low GI/GL*** |  |  |  |  |  |  |  |  |
| 0.22  (0.09, 0.5) | 0.76  (0.38, 1.53) | 0.32  (0.01, 4.02) | 2.05  (1.08, 4.14) | 0.86  (0.6, 1.21) | ***High Fiber*** |  |  |  |  |  |  |  |
| 0.28  (0.1, 0.75) | 0.99  (0.4, 2.33) | 0.41  (0.01, 5.28) | 2.67  (0.85, 8.36) | 1.11  (0.4, 2.99) | 1.29  (0.44, 3.66) | ***ER*** |  |  |  |  |  |  |
| 0.1  (0.01, 1.16) | 0.36  (0.03, 3.81) | 0.14  (0, 4.47) | 0.98  (0.09, 12.07) | 0.4  (0.04, 4.58) | 0.47  (0.04, 5.59) | 0.37  (0.03, 4.55) | ***HCC*** |  |  |  |  |  |
| 0.29  (0.03, 2.79) | 1  (0.11, 9.29) | 0.4  (0.01, 11.66) | 2.72  (0.27, 29.42) | 1.12  (0.12, 11.19) | 1.31  (0.14, 13.4) | 1.02  (0.11, 10.97) | 2.73  (1.28, 6.86) | ***Soy*** |  |  |  |  |
| 0.51  (0.18, 1.43) | 1.76  (0.75, 4.54) | 0.74  (0.02, 9.82) | 4.79  (1.54, 16.24) | 1.99  (0.74, 5.73) | 2.31  (0.81, 7.18) | 1.8  (0.56, 6.25) | 4.93  (0.41, 59.39) | 1.77  (0.17, 18.34) | ***AE*** |  |  |  |
| 0.88  (0.37, 2.16) | 3.04  (1.55, 6.78) | 1.28  (0.04, 16.14) | 8.3  (3.04, 24.66) | 3.42  (1.51, 8.72) | 4  (1.64, 10.72) | 3.12  (1.09, 9.63) | 8.59  (0.75, 97.65) | 3.08  (0.3, 29.72) | 1.74  (0.59, 5.03) | ***RE*** |  |  |
| 0.67  (0.21, 2.14) | 2.36  (0.81, 6.88) | 0.97  (0.03, 14.03) | 6.4  (1.79, 23.51) | 2.65  (0.85, 8.57) | 3.09  (0.94, 10.56) | 2.41  (0.64, 9.14) | 6.53  (0.5, 82.13) | 2.36  (0.2, 25.82) | 1.34  (0.36, 4.87) | 0.77  (0.35, 1.59) | ***HFLC*** |  |
| 0.29  (0.16, 0.47) | 1.01  (0.78, 1.3) | 0.43  (0.01, 4.83) | 2.72  (1.25, 6.1) | 1.13  (0.66, 1.99) | 1.32  (0.69, 2.56) | 1.03  (0.45, 2.4) | 2.81  (0.27, 28.76) | 1  (0.11, 8.75) | 0.57  (0.23, 1.3) | 0.33  (0.16, 0.62) | 0.43  (0.15, 1.2) | ***Control*** |

***BIRTH WEIGHT***

| ***DASH*** |  |  |  |  |  |  |  |  |  |  |  |  |  |  |
| --- | --- | --- | --- | --- | --- | --- | --- | --- | --- | --- | --- | --- | --- | --- |
| -541.5  (-805.22, -278.81) | ***Low Carb*** |  |  |  |  |  |  |  |  |  |  |  |  |  |
| -519.19  (-894.2, -146.7) | 22.37  (-243.53, 284.77) | ***High Carb*** |  |  |  |  |  |  |  |  |  |  |  |  |
| -521.47  (-1099.48, 48.8) | 20.99  (-486.94, 530.68) | -1.75  (-437.87, 430.71) | ***High MUFA*** |  |  |  |  |  |  |  |  |  |  |  |
| -407.47  (-593.09, -222.71) | 135.54  (-88.06, 356.02) | 110.82  (-230.42, 460.88) | 112.04  (-445.71, 674.47) | ***Low GI/GL*** |  |  |  |  |  |  |  |  |  |  |
| -408.01  (-745.91, -73.56) | 133.68  (-220.78, 491.83) | 111.69  (-326.89, 554.77) | 114.6  (-509.96, 739.99) | -1.77  (-276.74, 277.16) | ***High Fiber*** |  |  |  |  |  |  |  |  |  |
| -647.1  (-1756.18, 447.44) | -106.3  (-1216.54, 989.46) | -128.49  (-1277.4, 1004.06) | -126.43  (-1362.62, 1080.91) | -243.58  (-1339.01, 842.8) | -239.2  (-1371.3, 880.53) | ***HCC*** |  |  |  |  |  |  |  |  |
| -443.52  (-720.03, -169.92) | 99.3  (-201.26, 397.96) | 76.25  (-324.06, 471.7) | 78.7  (-519.63, 670.55) | -37.28  (-267.72, 198.82) | -35.97  (-397.57, 328.36) | 206.89  (-859.19, 1269.74) | ***Soy*** |  |  |  |  |  |  |  |
| -510.92  (-963.03, -64.28) | 32.15  (-426.42, 499) | 11.02  (-530.65, 547.9) | 11.88  (-682.17, 702.71) | -104.74  (-527.55, 326.81) | -103.4  (-602.87, 405.16) | 137.97  (-1025.85, 1307.61) | -64.54  (-534.54, 400.13) | ***PMW*** |  |  |  |  |  |  |
| -621.69  (-825.82, -417.73) | -79.04  (-318.68, 159.65) | -104.21  (-451.36, 252.36) | -101.86  (-658.41, 466.57) | -214.77  (-361.28, -69.37) | -212.83  (-531.62, 100.75) | 26.96  (-1065.58, 1126.53) | -177.44  (-427, 69.94) | -110.54  (-507.92, 286.75) | ***AE*** |  |  |  |  |  |
| -531.35  (-736.51, -328.7) | 9.94  (-227.99, 246.97) | -13.78  (-362.98, 350.56) | -10.64  (-570.78, 557.36) | -124.55  (-271.53, 22.25) | -123.9  (-441.92, 190.1) | 119.82  (-978.14, 1216.28) | -86.69  (-336.36, 160.32) | -21.3  (-443.77, 398.5) | 89.55  (-43.1, 223.1) | ***RE*** |  |  |  |  |
| -807.22  (-1915.2, 294.31) | -264.51  (-1373.54, 839.54) | -285.93  (-1438.99, 845.31) | -281.52  (-1520.94, 928.64) | -401.01  (-1496.89, 689.35) | -396.39  (-1530.03, 727.82) | -157.69  (-229.02, -88.13) | -362.48  (-1433.01, 703.92) | -297.15  (-1466.87, 872.74) | -184.33  (-1295.6, 909.23) | -275.48  (-1378.17, 819.81) | ***HFLC*** |  |  |  |
| -557.73  (-769.7, -343) | -14.96  (-263.49, 228.32) | -36.96  (-396.35, 325.38) | -38  (-605.71, 535.02) | -150.69  (-309.89, 9.82) | -149.67  (-466.61, 173.71) | 92.24  (-999.27, 1190.44) | -113.17  (-371.21, 143.32) | -46.45  (-486.3, 388.34) | 64.44  (-114.93, 241.98) | -25.18  (-204.88, 156.34) | 249.61  (-841.75, 1348.77) | ***SE*** |  |  |
| -467.18  (-704.41, -227.31) | 74.01  (-190.88, 345.34) | 53.21  (-315.38, 434.39) | 55.3  (-518.08, 631.66) | -59.51  (-248.79, 130.6) | -58.84  (-396.63, 280.01) | 184.61  (-916.49, 1282.47) | -22.48  (-303.7, 254.1) | 42.85  (-411.51, 496.53) | 154.69  (-53.77, 363.14) | 65.67  (-144.6, 273.15) | 341.63  (-755.15, 1443.23) | 91.01  (-129.09, 305.46) | ***ARE*** |  |
| -586.91  (-754.93, -424.09) | -45.81  (-251.29, 160.24) | -68.58  (-398.13, 269.68) | -65.6  (-617.75, 485.88) | -180.25  (-265.76, -93.91) | -179.43  (-470.23, 109.01) | 60.5  (-1023.32, 1152.92) | -143.32  (-364.53, 74.85) | -74.98  (-496.21, 337.8) | 33.99  (-84.51, 151.36) | -55.87  (-173.37, 65.04) | 219.49  (-863.45, 1311.82) | -29.78  (-166.18, 104.21) | -121.27  (-292.19, 48.81) | ***Control*** |

***MACROSOMIA***

***Network plot after removing zero event***

| ***DASH*** |  | | | | | | | | | | |
| --- | --- | --- | --- | --- | --- | --- | --- | --- | --- | --- | --- |
| 0.18  (0.02, 0.98) | ***Low Carb*** |  | | | | | | | | | |
| 0.21  (0.02, 1.14) | 1.14  (0.27, 5.1) | ***Low GI/GL*** |  | | | | | | | | |
| 0.05  (0, 0.98) | 0.3  (0.01, 4.73) | 0.26  (0.01, 2.44) | ***High Fiber*** |  | | | | | | | |
| 0.18  (0.02, 1.49) | 1.02  (0.18, 6.31) | 0.9  (0.14, 5.87) | 3.41  (0.19, 107.35) | ***Soy*** |  | | | | | | |
| 0.14  (0.01, 5.53) | 0.86  (0.04, 30.08) | 0.73  (0.04, 28.87) | 2.99  (0.07, 320.61) | 0.84  (0.03, 33.67) | ***PMW*** |  | | | | | |
| 0.11  (0.01, 0.69) | 0.59  (0.11, 3.03) | 0.51  (0.09, 2.98) | 1.95  (0.12, 54.33) | 0.59  (0.08, 3.92) | 0.75  (0.03, 7.07) | ***AE*** |  | | | | |
| 0.19  (0.02, 1.14) | 1.09  (0.22, 4.99) | 0.91  (0.19, 4.32) | 3.53  (0.21, 113.01) | 1.05  (0.15, 6.3) | 1.3  (0.05, 19.67) | 1.81  (0.45, 7.56) | ***RE*** |  | | | |
| 0.18  (0.01, 6.48) | 1.05  (0.08, 36.12) | 0.93  (0.07, 32.75) | 3.76  (0.1, 305.06) | 1.05  (0.06, 36.1) | 1.34  (0.02, 69.91) | 1.81  (0.11, 65.57) | 1.03  (0.06, 30.34) | ***HFLC*** |  | | |
| 0.15  (0.02, 0.59) | 0.83  (0.23, 2.42) | 0.72  (0.2, 2.38) | 2.71  (0.2, 78.62) | 0.82  (0.15, 3.59) | 0.98  (0.03, 16.05) | 1.37  (0.34, 6.13) | 0.77  (0.21, 2.75) | 0.77  (0.03, 8.68) | ***SE*** |  | |
| 0.4  (0.03, 17.74) | 2.37  (0.23, 87.27) | 2.03  (0.17, 78.27) | 8.52  (0.25, 923.28) | 2.33  (0.15, 87.61) | 2.81  (0.05, 253.42) | 4.02  (0.3, 181.83) | 2.22  (0.18, 94.64) | 2.28  (0.04, 164.44) | 2.77  (0.3, 96.94) | ***ARE*** |  |
| 0.11  (0.01, 0.36) | 0.58  (0.2, 1.5) | 0.51  (0.15, 1.39) | 1.89  (0.15, 52.59) | 0.58  (0.12, 2.26) | 0.69  (0.02, 10.05) | 0.96  (0.26, 3.74) | 0.55  (0.16, 1.65) | 0.54  (0.02, 5.78) | 0.7  (0.39, 1.18) | 0.25  (0.01, 2.31) | ***Control*** |

***MACROSOMIA***

***Network plot after removing all heterogeneous studies***

| ***DASH*** |  | | | | | | | | | | | |
| --- | --- | --- | --- | --- | --- | --- | --- | --- | --- | --- | --- | --- |
| 0.18  (0.02, 0.96) | ***Low Carb*** |  | | | | | | | | | | |
| 2407.58 (0, 3.51124929316492e+25) | 13852.63 (0, 2.26072237769593e+26) | ***High Carb*** |  | | | | | | | | |  |
| 0.21  (0.02, 1.22) | 1.16  (0.26, 5.39) | 0 (0, 39172858288023.8) | ***Low GI/GL*** |  | | | | | | | |  |
| 0.05  (0, 0.94) | 0.28  (0.01, 4.3) | 0 (0, 9366678138586.87) | 0.25  (0.01, 2.29) | ***High Fiber*** |  | | | | | | |  |
| 0.18  (0.02, 1.42) | 0.99  (0.18, 6.46) | 0 (0, 39928455989839) | 0.85  (0.14, 5.84) | 3.57  (0.19, 171.54) | ***Soy*** |  | | | | | |  |
| 0.15  (0.01, 6.46) | 0.85  (0.04, 31.46) | 0 (0, 37606004249638.9) | 0.72  (0.03, 28.8) | 3.22  (0.07, 402.44) | 0.84  (0.04, 35.09) | ***PMW*** |  | | | | |  |
| 0.11  (0.01, 0.7) | 0.58  (0.11, 3.16) | 0 (0, 22468199609987.6) | 0.5  (0.09, 2.85) | 2.06  (0.12, 85.59) | 0.59  (0.07, 4.13) | 0.72  (0.03, 7.92) | ***AE*** |  | | | |  |
| 0.19  (0.02, 1.19) | 1.05  (0.22, 5.29) | 0 (0, 40512505831997.4) | 0.9  (0.17, 4.72) | 3.7  (0.23, 154.18) | 1.06  (0.15, 6.81) | 1.26  (0.04, 21.66) | 1.8  (0.46, 7.77) | ***RE*** |  | | |  |
| 0.19  (0.01, 7.33) | 1.07  (0.07, 38.31) | 0 (0, 46902979841554.1) | 0.92  (0.06, 32.35) | 4.08  (0.11, 495.55) | 1.09  (0.06, 42.17) | 1.3  (0.02, 102.74) | 1.84  (0.1, 74.25) | 1.01  (0.06, 39.07) | ***HFLC*** |  | |  |
| 0.43  (0.02, 15.57) | 2.34  (0.2, 77.51) | 0 (0, 109321930777191) | 2.02  (0.16, 68.75) | 9.13  (0.26, 921.26) | 2.36  (0.15, 83.51) | 2.9  (0.04, 215.21) | 4.08  (0.29, 151.35) | 2.25  (0.17, 78.19) | 2.21  (0.04, 134.35) | ***ARE*** |  |  |
| 0.11  (0.02, 0.37) | 0.57  (0.2, 1.51) | 0 (0, 21258987063684.1) | 0.49  (0.15, 1.41) | 1.95  (0.16, 66.14) | 0.58  (0.11, 2.25) | 0.69  (0.02, 10.81) | 0.97  (0.25, 3.76) | 0.54  (0.15, 1.73) | 0.54  (0.02, 6.66) | 0.25  (0.01, 2.3) | ***Control*** |  |

***PRETERM BIRTH***

***Network plot after removing zero event***

| ***Low GI/GL*** |  |  |  |  |  |  |
| --- | --- | --- | --- | --- | --- | --- |
| 0.96  (0.1, 8.95) | ***High Fiber*** |  |  |  |  |  |
| 0.35  (0.01, 4.69) | 0.35  (0.01, 11.19) | ***Soy*** |  |  |  |  |
| 1.71  (0.53, 6.23) | 1.77  (0.15, 23.33) | 5.01  (0.31, 183.91) | ***AE*** |  |  |  |
| 0.46  (0.16, 1.25) | 0.47  (0.04, 5.46) | 1.3  (0.09, 43.88) | 0.27  (0.08, 0.81) | ***RE*** |  |  |
| 1.08  (0.47, 2.42) | 1.1  (0.1, 11.99) | 3.06  (0.22, 99.06) | 0.63  (0.16, 2.09) | 2.34  (0.81, 7.12) | ***SE*** |  |
| 0.83  (0.49, 1.44) | 0.87  (0.09, 8.77) | 2.39  (0.19, 74.21) | 0.49  (0.15, 1.37) | 1.83  (0.79, 4.61) | 0.78  (0.42, 1.48) | ***Control*** |

***PRETERM BIRTH***

***Network plot after removing all heterogeneous studies***

| ***Soy*** |  |  |  |  |  |
| --- | --- | --- | --- | --- | --- |
| 4.93  (0.33, 152.96) | ***AE*** |  |  |  |  |
| 1.29  (0.09, 39.21) | 0.27  (0.08, 0.79) | ***RE*** |  |  |  |
| 8565.1 (0, 1.37112929747485e+22) | 1598.08 (0, 2.03072057511937e+21) | 6240.21 (0, 9.04730543333287e+21) | ***HFLC*** |  |  |
| 3.01  (0.23, 86.1) | 0.63  (0.16, 2.08) | 2.35  (0.82, 7.08) | 0 (0, 129293941265.84) | ***SE*** |  |
| 2.32  (0.2, 65.58) | 0.49  (0.16, 1.37) | 1.83  (0.79, 4.49) | 0 (0, 100922010164.91) | 0.78  (0.42, 1.48) | ***Control*** |

The red numbers represent a significant difference, and the yellow tables indicate a significant change compared to the original results.

***Supplementary S13: SUCRA for Sensitive Analysis***

*AE: Aerobic Exercise; ARE: Aerobic + Resistance Exercise; CON: Control; DAS: DASH; ER: Energy Restriction; HC: High Carbohydrates; HCC: High Complex Carbohydrates; HF: High Fiber; HFL: Higher fat/Lower Carbohydrates; HGI: High GI/GL; HMU: High MUFA (Monounsaturated Fatty Acid); LC: Low Carbohydrates; LGI: Low GI/GL; LGY: Low GI + Yoga; PMW: Postmeal Walking; RE: Resistance Exercise; SE: Structured Exercise; Soy: Soy Protein;*

***FASTING PLASMA GLUCOSE (Big Loop)***

| ***DAS*** | ***ARE*** | ***LGY*** | ***RE*** | ***CON*** | ***HC*** | ***LC*** | ***HCC*** | ***HMU*** | ***HFL*** |
| --- | --- | --- | --- | --- | --- | --- | --- | --- | --- |
| 1 | 0.85 | 0.74 | 0.61 | 0.46 | 0.37 | 0.34 | 0.32 | 0.22 | 0.10 |

***2h POSTPRANDIAL GLUCOSE***

***(Big Loop)***

| ***DAS*** | ***LGY*** | ***ARE*** | ***LGI*** | ***RE*** | ***CON*** | ***AE*** | ***HC*** | ***HMU*** | ***LC*** |
| --- | --- | --- | --- | --- | --- | --- | --- | --- | --- |
| 0.98 | 0.83 | 0.81 | 0.68 | 0.55 | 0.36 | 0.36 | 0.19 | 0.12 | 0.12 |

***(Small Loop)***

| ***HCC*** | ***SOY*** | ***HFL*** |
| --- | --- | --- |
| 0.65 | 0.50 | 0.34 |

***INSULIN REQUIREMENTS***

***Network plot after removing zero event***

| ***DAS*** | ***RE*** | ***HFL*** | ***HC*** | ***AE*** | ***SOY*** | ***CON*** | ***LC*** | ***ER*** | ***LGI*** | ***HF*** | ***HCC*** | ***HGI*** |
| --- | --- | --- | --- | --- | --- | --- | --- | --- | --- | --- | --- | --- |
| 0.89 | 0.86 | 0.76 | 0.69 | 0.67 | 0.47 | 0.44 | 0.43 | 0.42 | 0.37 | 0.28 | 0.16 | 0.07 |

***BIRTH WEIGHT***

| ***DAS*** | ***LGI*** | ***HF*** | ***SOY*** | ***ARE*** | ***PMW*** | ***HMU*** | ***HC*** | ***RE*** | ***HCC*** | ***LC*** | ***SE*** | ***CON*** | ***HFL*** | ***AE*** |
| --- | --- | --- | --- | --- | --- | --- | --- | --- | --- | --- | --- | --- | --- | --- |
| 0.98 | 0.73 | 0.66 | 0.63 | 0.593 | 0.49 | 0.47 | 0.47 | 0.45 | 0.43 | 0.42 | 0.39 | 0.30 | 0.26 | 0.23 |

***MACROSOMIA***

***Network plot after removing zero event***

| ***DAS*** | ***ARE*** | ***LGI*** | ***RE*** | ***HFL*** | ***LC*** | ***SOY*** | ***PMW*** | ***SE*** | ***AE*** | ***CON*** | ***HF*** |
| --- | --- | --- | --- | --- | --- | --- | --- | --- | --- | --- | --- |
| 0.93 | 0.73 | 0.58 | 0.54 | 0.52 | 0.52 | 0.52 | 0.46 | 0.44 | 0.31 | 0.24 | 0.21 |

***Network plot after removing all heterogeneous studies***

| ***DAS*** | ***ARE*** | ***HC*** | ***LGI*** | ***RE*** | ***HFL*** | ***LC*** | ***SOY*** | ***PMW*** | ***AE*** | ***CON*** | ***HF*** |
| --- | --- | --- | --- | --- | --- | --- | --- | --- | --- | --- | --- |
| 0.87 | 0.68 | 0.67 | 0.55 | 0.52 | 0.50 | 0.50 | 0.49 | 0.45 | 0.31 | 0.26 | 0.21 |

***PRETERM BIRTH***

***Network plot after removing zero event***

| ***AE*** | ***SE*** | ***LGI*** | ***HF*** | ***CON*** | ***SOY*** | ***RE*** |
| --- | --- | --- | --- | --- | --- | --- |
| 0.84 | 0.64 | 0.60 | 0.55 | 0.45 | 0.25 | 0.17 |

***Network plot after removing all heterogeneous studies***

| ***AE*** | ***HFL*** | ***SE*** | ***CON*** | ***SOY*** | ***RE*** |
| --- | --- | --- | --- | --- | --- |
| 0.78 | 0.68 | 0.62 | 0.46 | 0.26 | 0.21 |

*The letters in yellow represent a significant change in ranking compared to the original results.*

***Supplementary S14: Network Plot for Sensitive Analysis***

***FASTING PLASMA GLUCOSE***

***
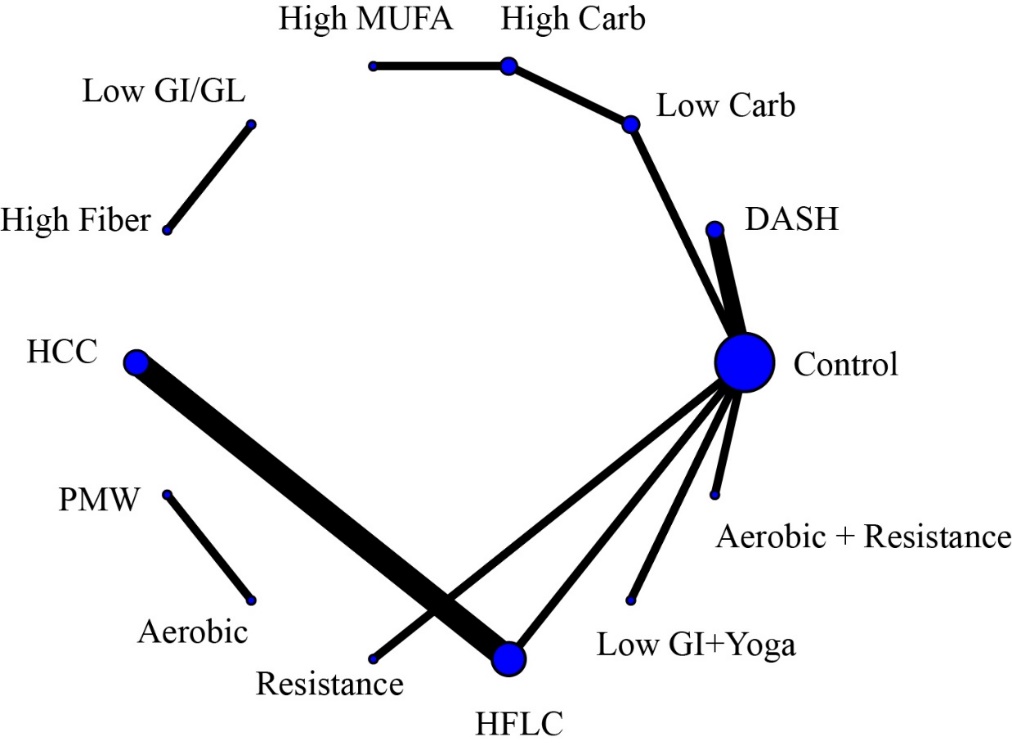
***

***2h POSTPRANDIAL GLUCOSE***

***
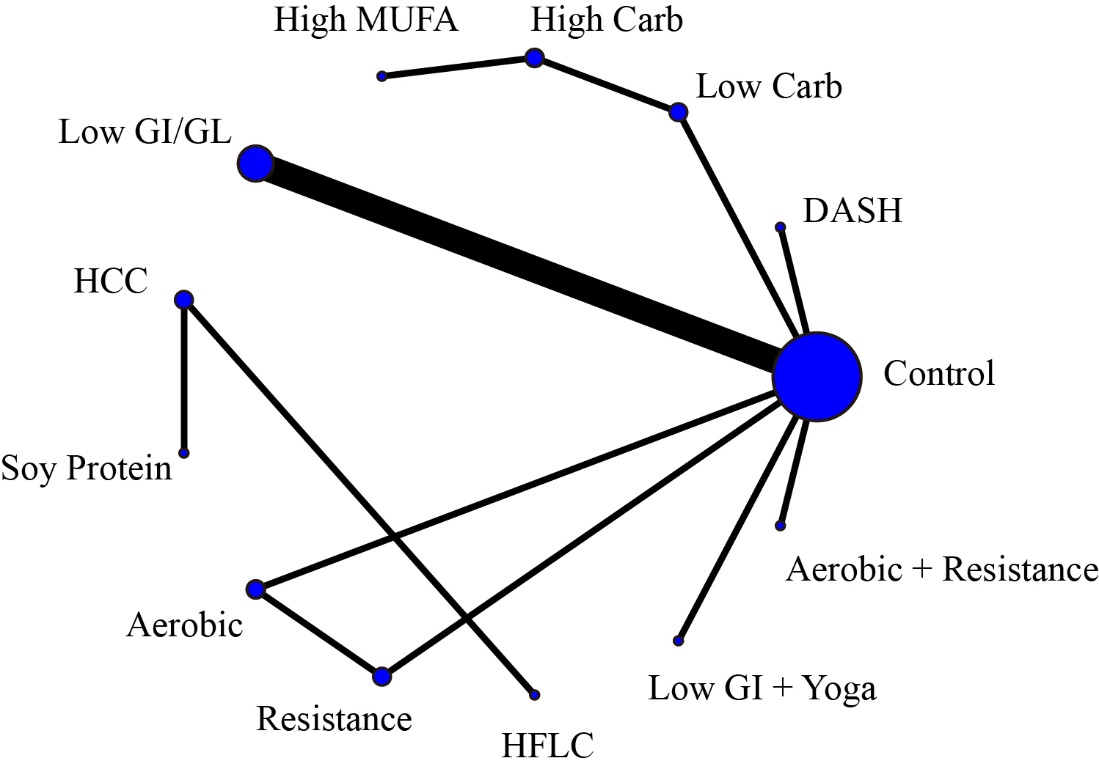
***

***INSULIN REQUIREMENTS***

***Network plot after removing zero event***

***
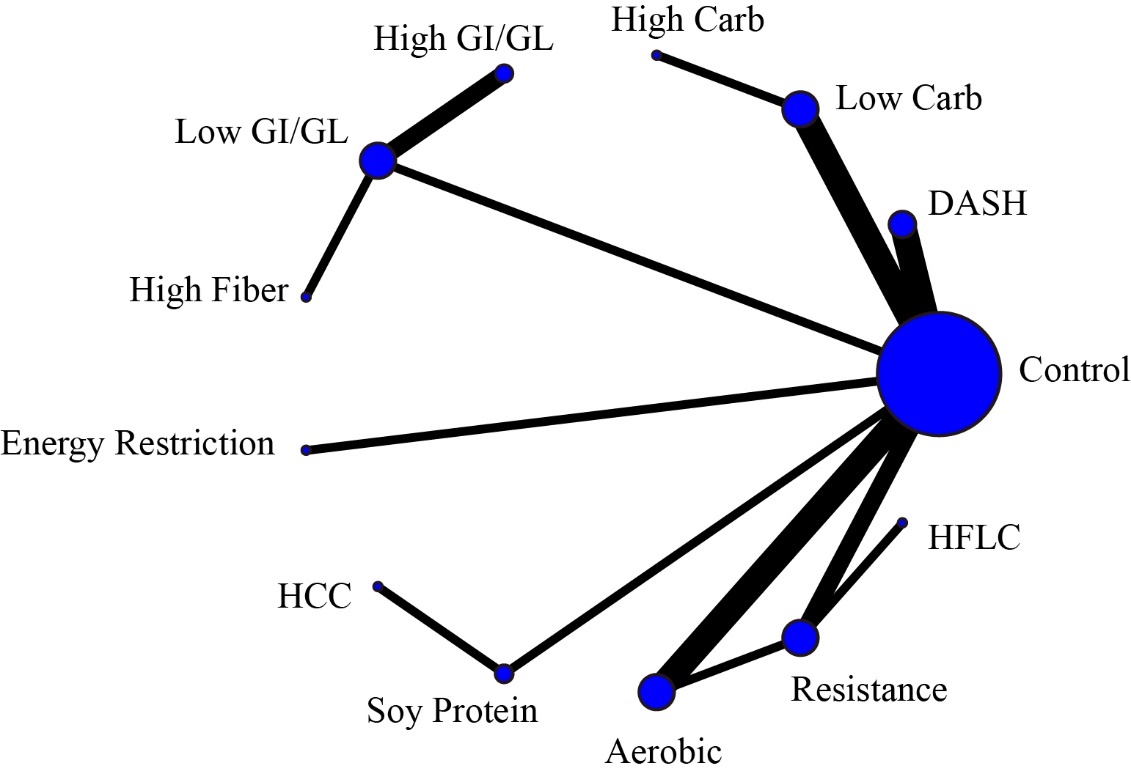
***

***BIRTH WEIGHT***

***
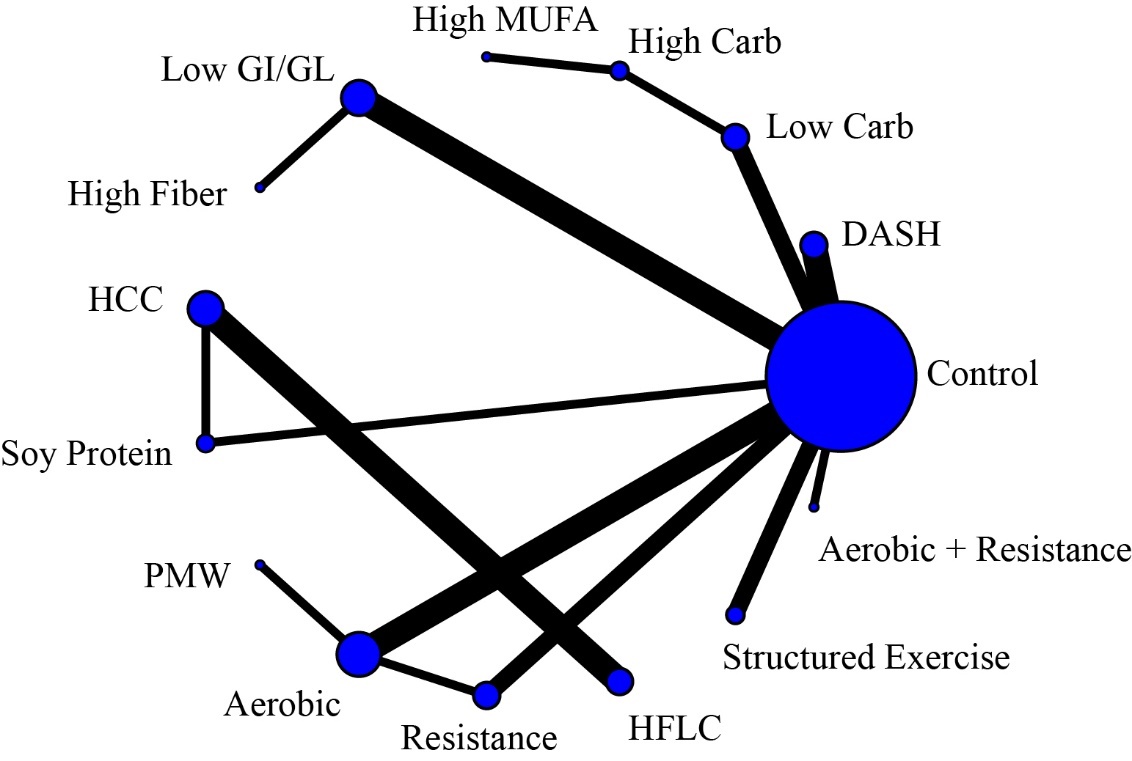
***

***MACROSOMIA***

***Network plot after removing zero event***

***
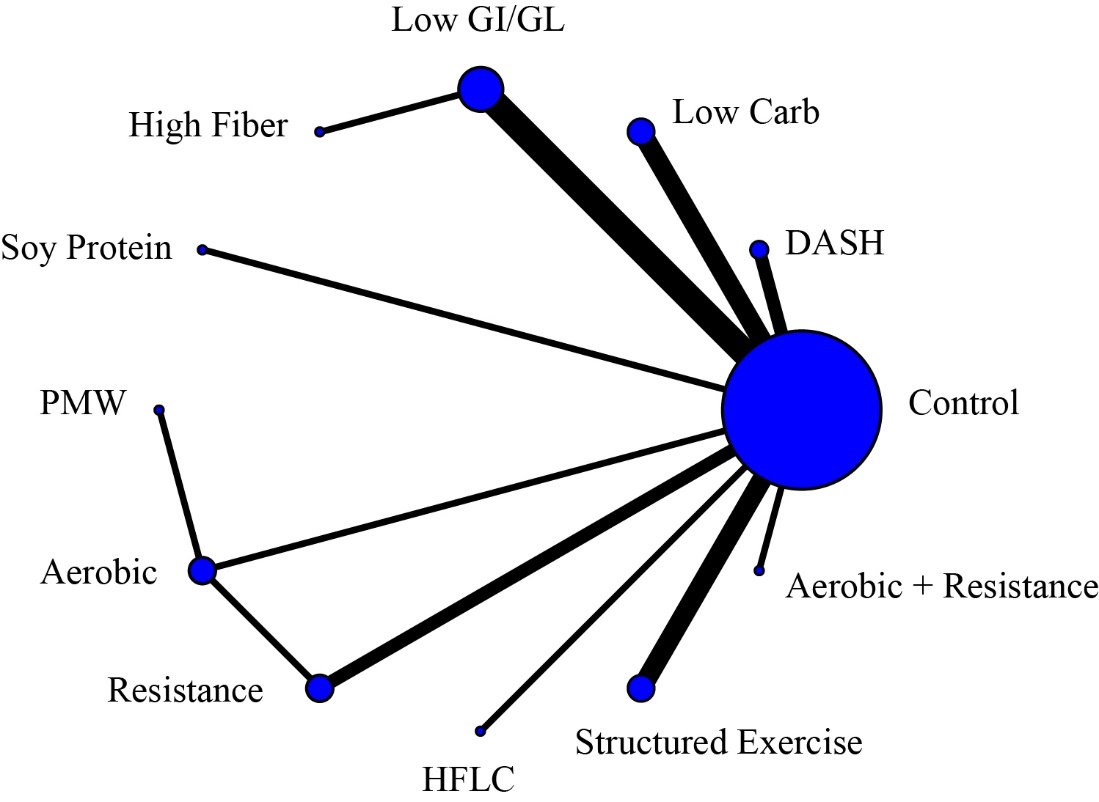
***

***Network plot after removing all heterogeneous studies***

***
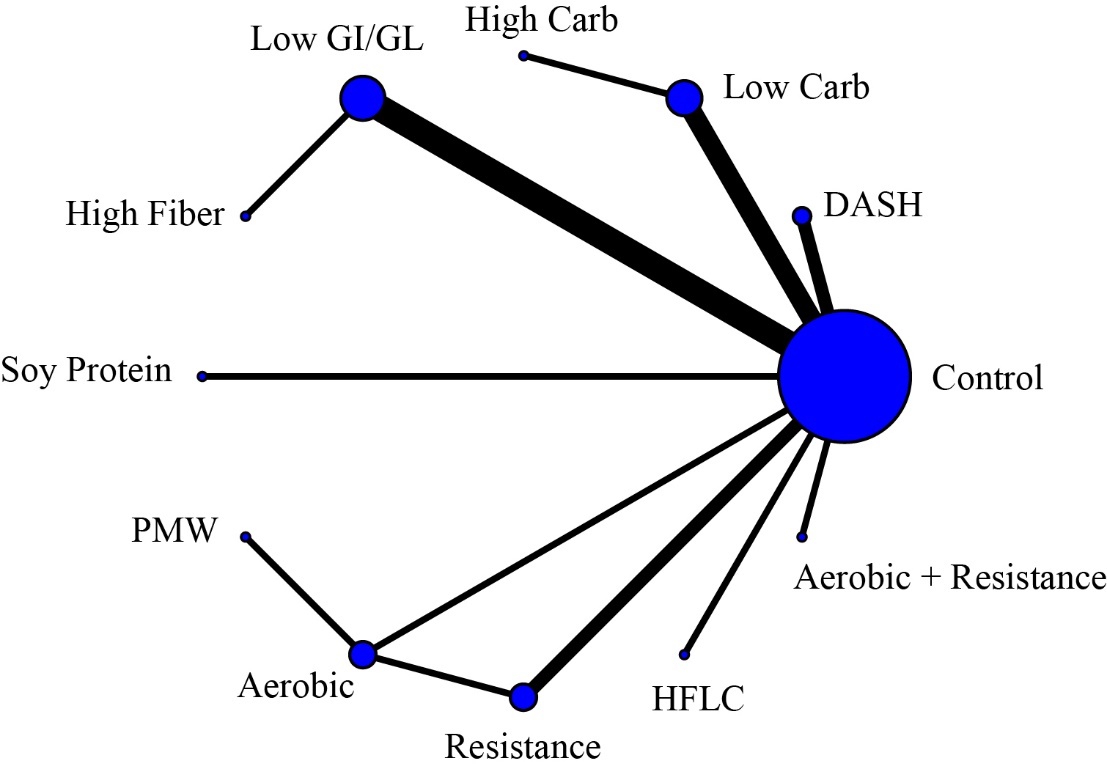
***

***PRETERM BIRTH***

***Network plot after removing zero event***

***
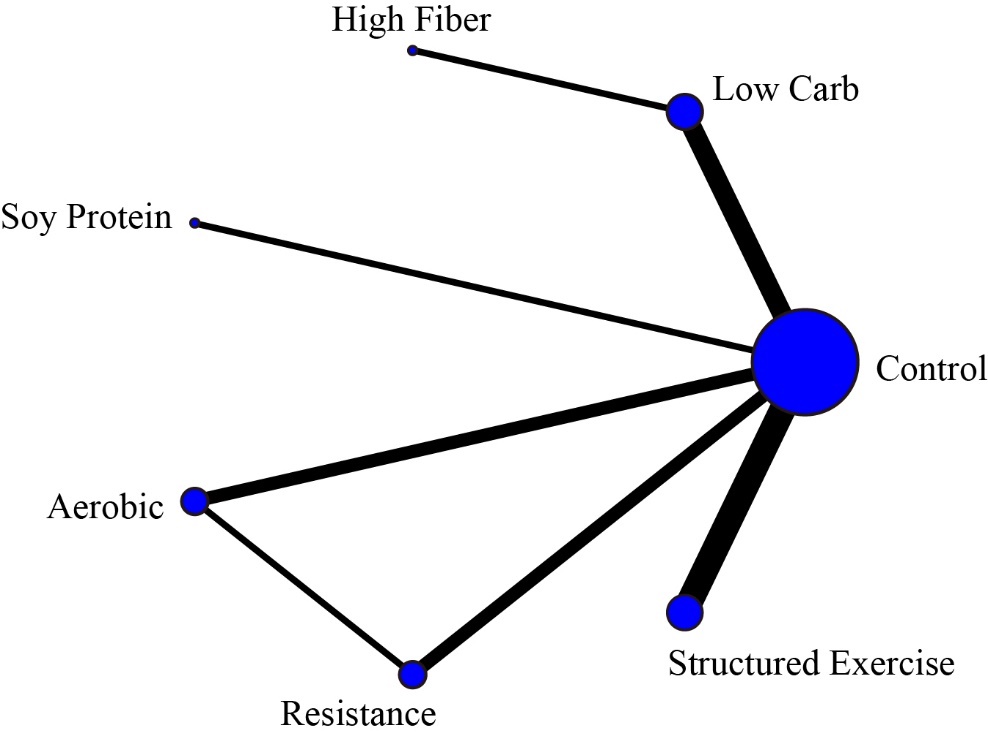
***

***Network plot after removing all heterogeneous studies***

***
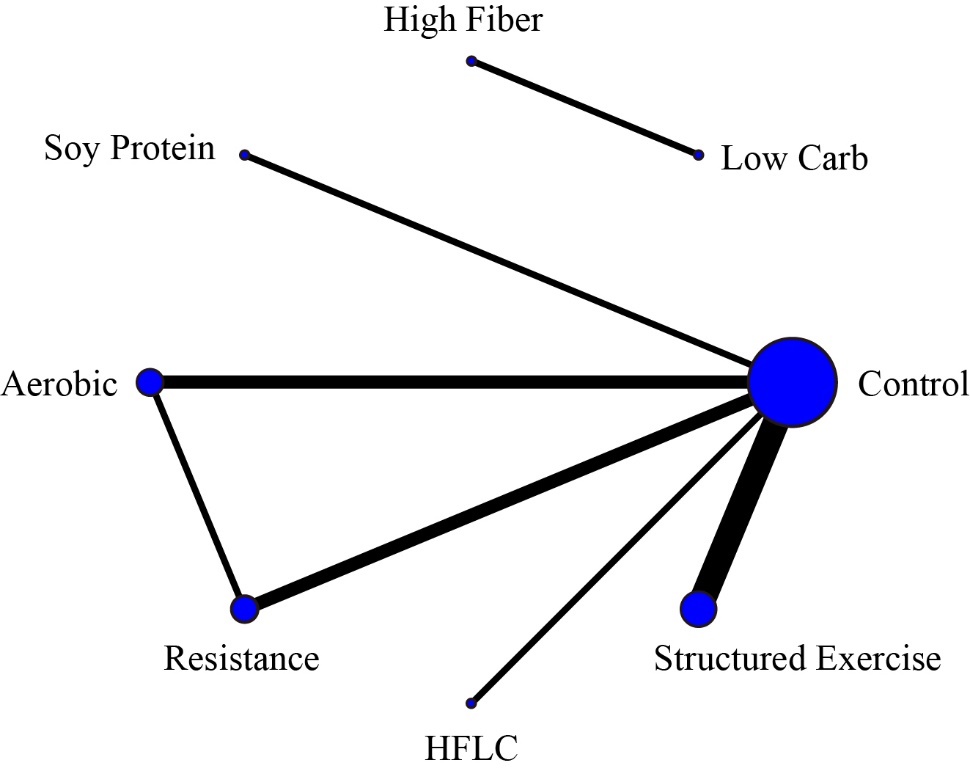
***

***Supplementary S15: Funnel plots***

***FASTING PLASMA GLUCOSE***

***
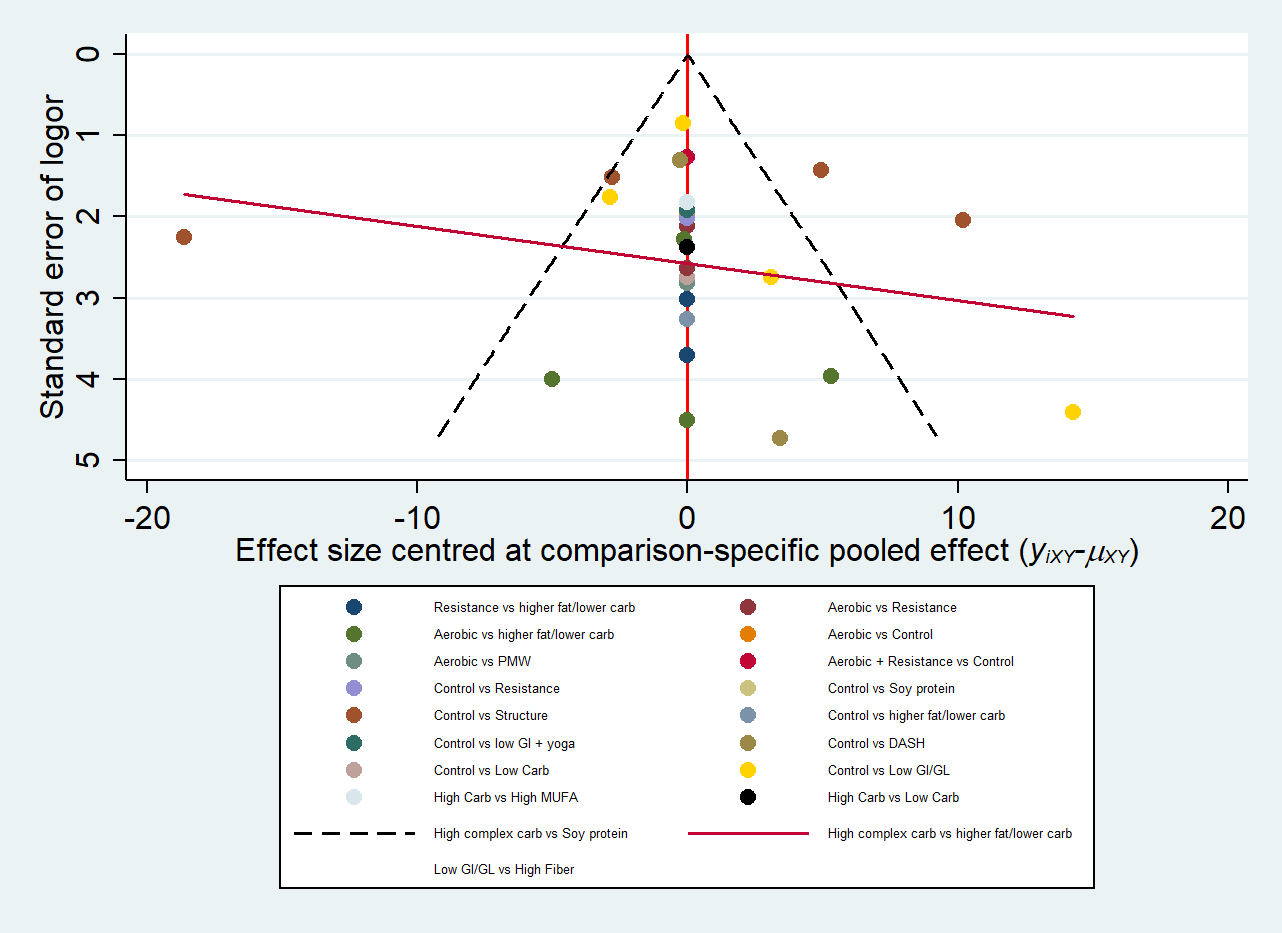
***

***2h POSTPRANDIAL GLUCOSE***

***
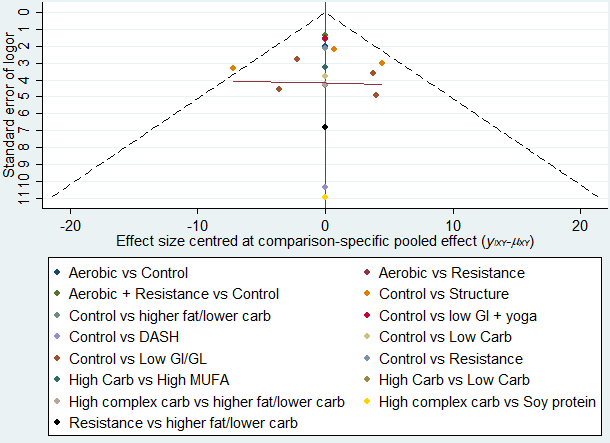
***

***INSULIN REQUIREMENTS***

***
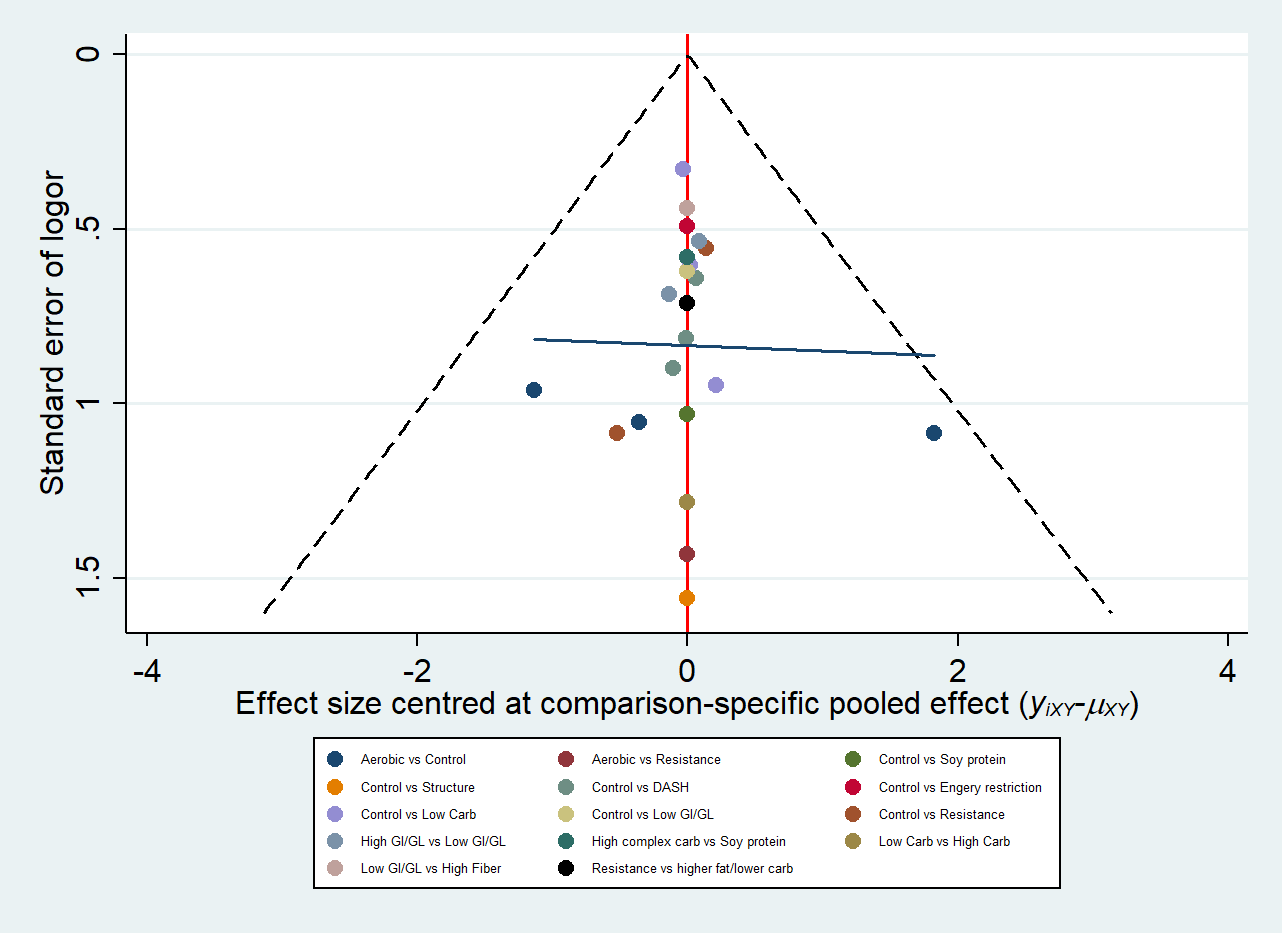
***

***BIRTH WEIGHT***

***
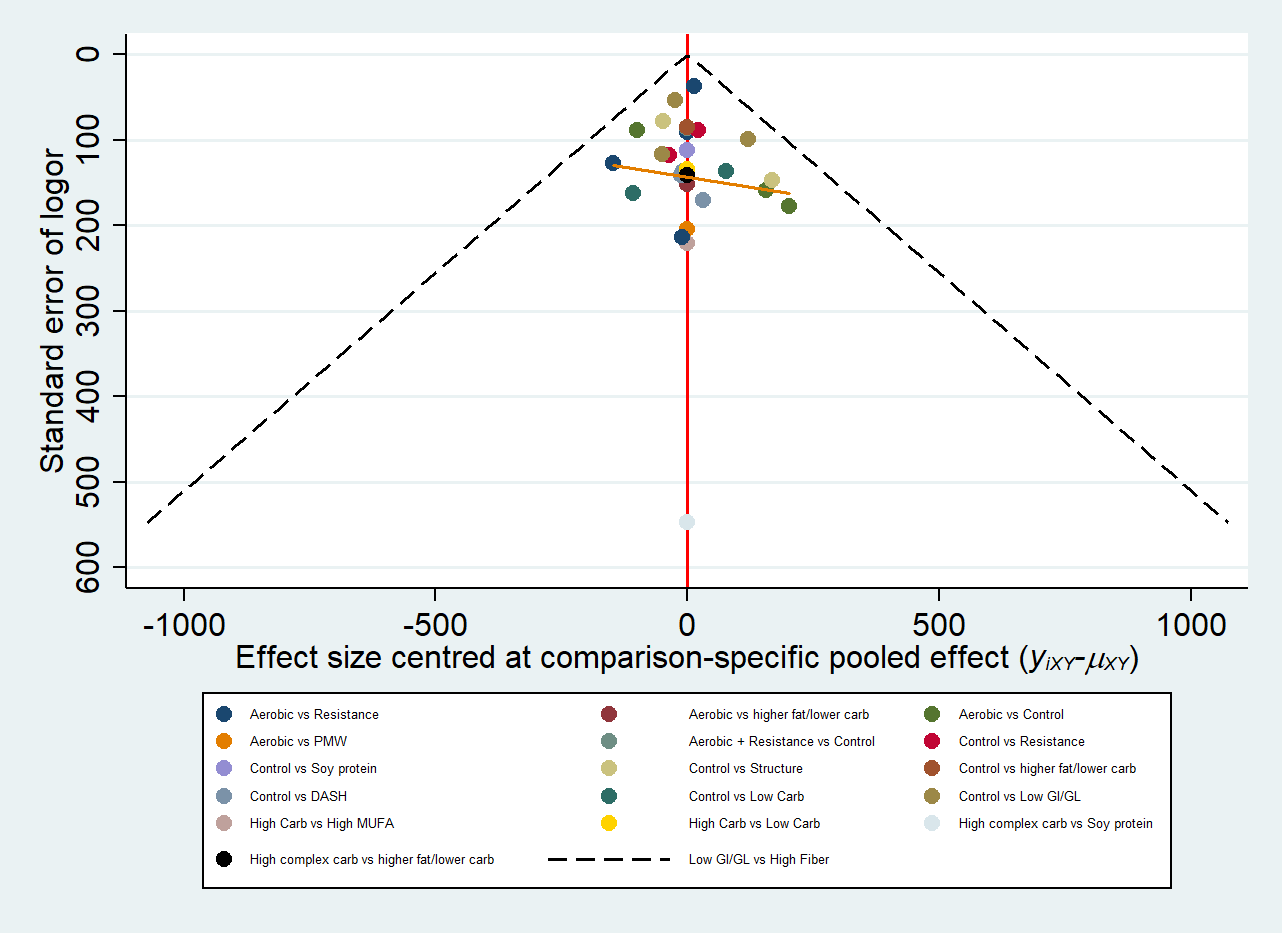
***

***MACROSOMIA***

***
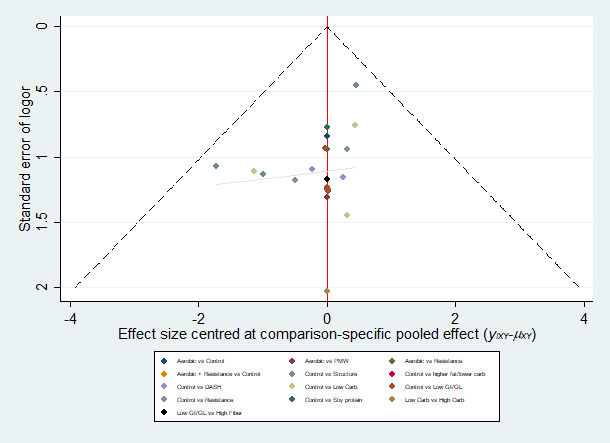
***

***PRETERM BIRTH***

***
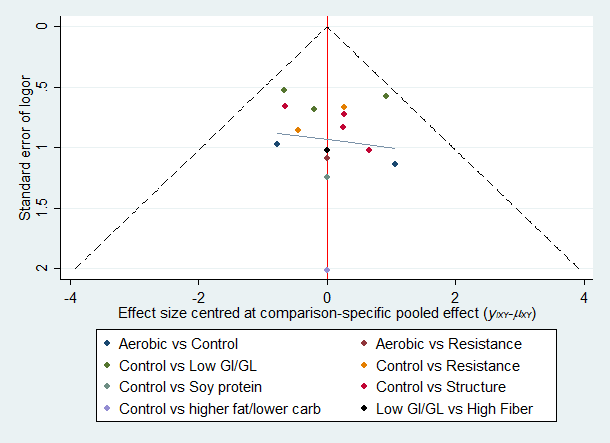
***

***Supplementary S16:*** ***Trace-density plots for Bayesian models***

*1. Control; 2. DASH; 3. Low Carbohydrates; 4. High Carbohydrates; 5. High MUFA; 6. High GI/GL; 7. Low GI/GL; 8. High Fiber; 9. Energy Restriction; 12. High Complex Carbohydrates; 13. Soy Protein; 14. PMW; 15. Aerobic Exercise; 16. Resistance Exercise; 17. Higher Fiber/Lower Carbohydrates; 18. Structured Exercise; 20. Low GI + Yoga; 21. Aerobic + Resistance Exercise.*

***FASTING PLASMA GLUCOSE***

***
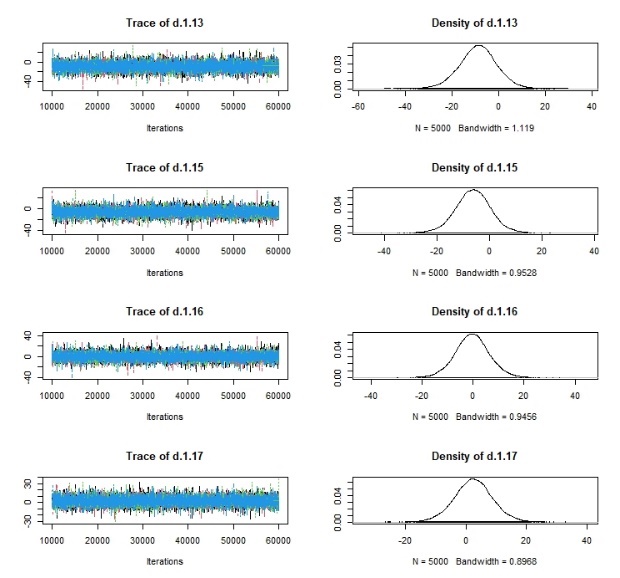

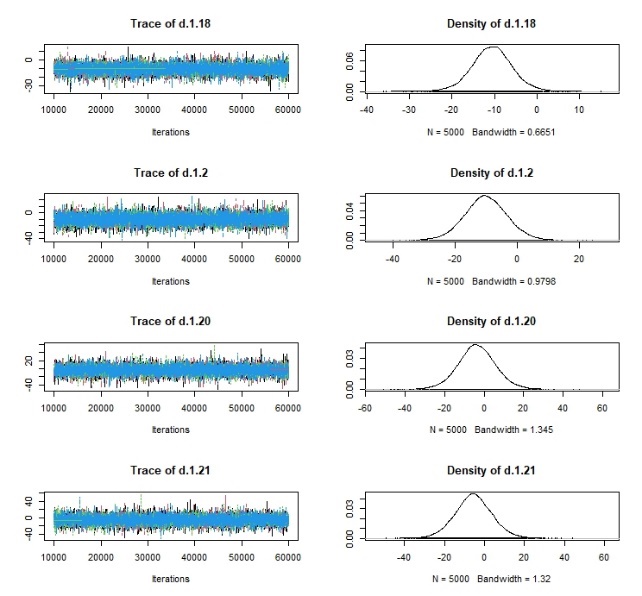
***

***
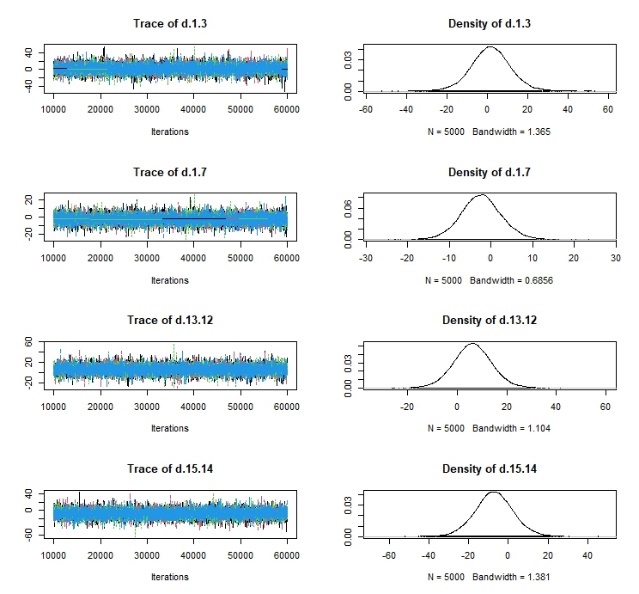

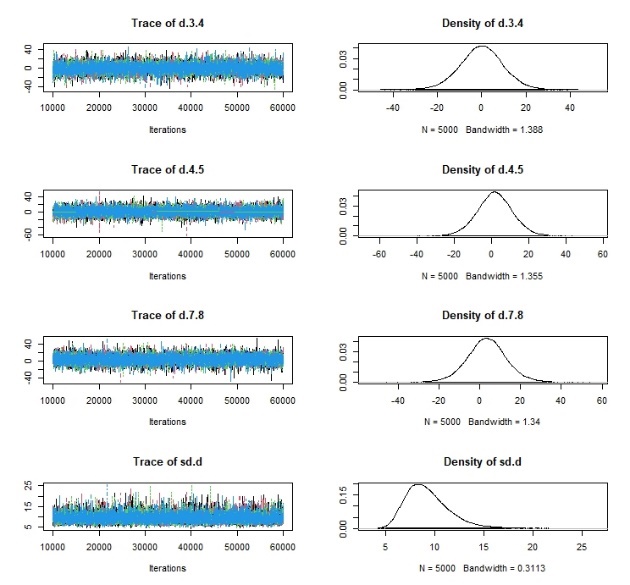
***

***2h POSTPRANDIAL GLUCOSE***

***
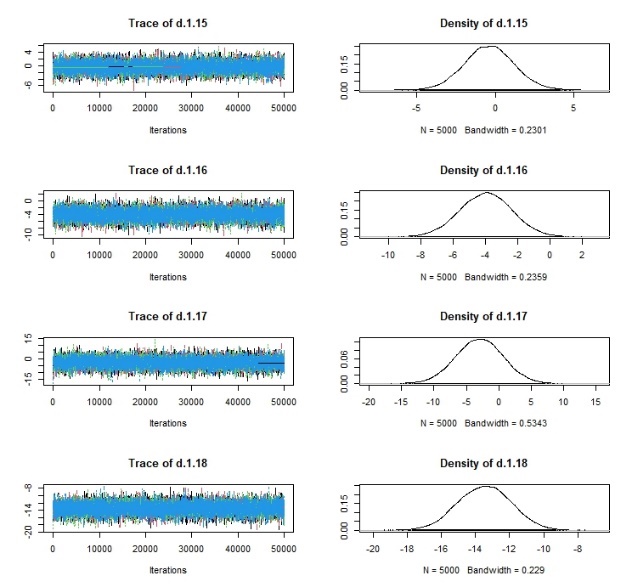

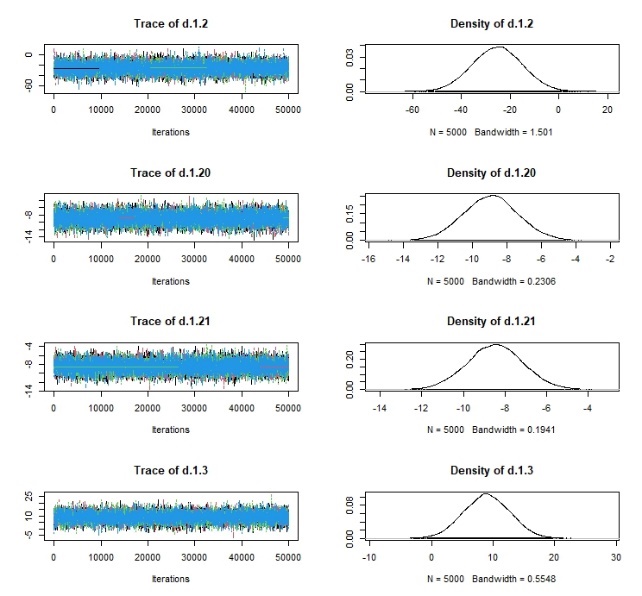
***

***
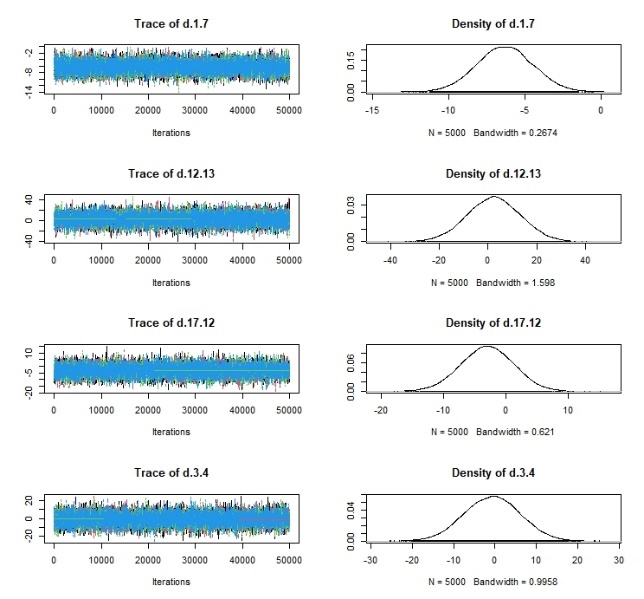
****
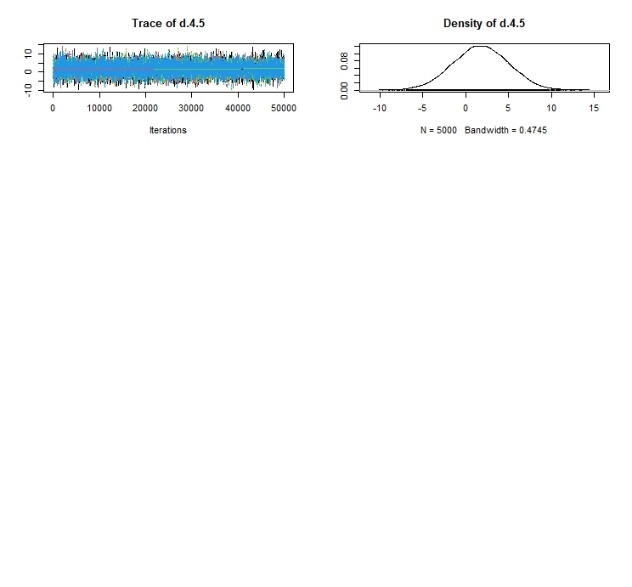
*

***INSULIN REQUIREMENTS***

***
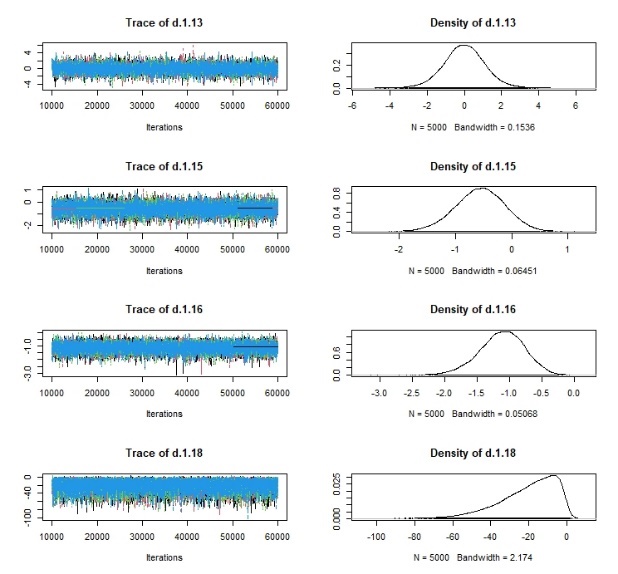

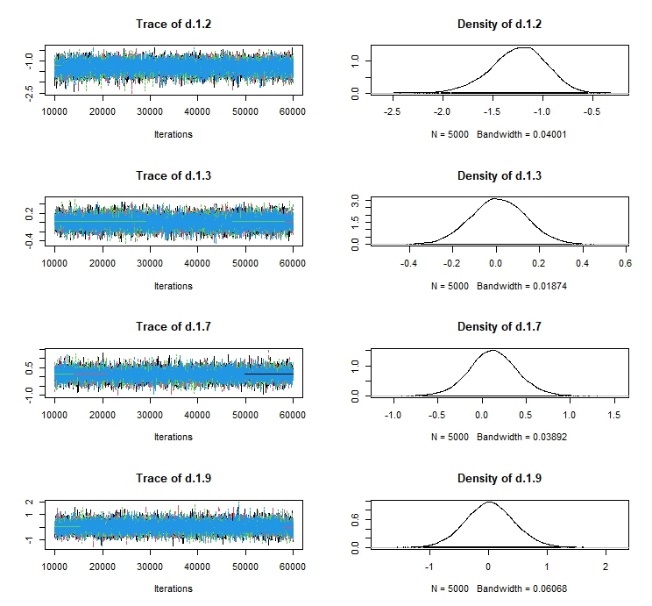
***

***
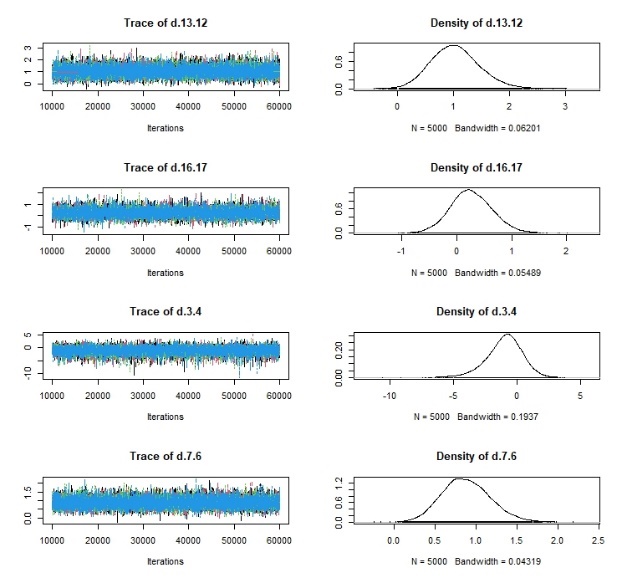

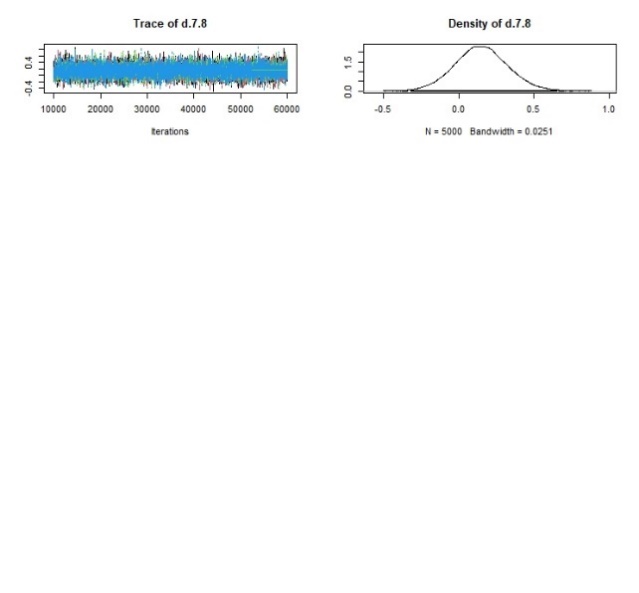
***

***BIRTH WEIGHT***

***
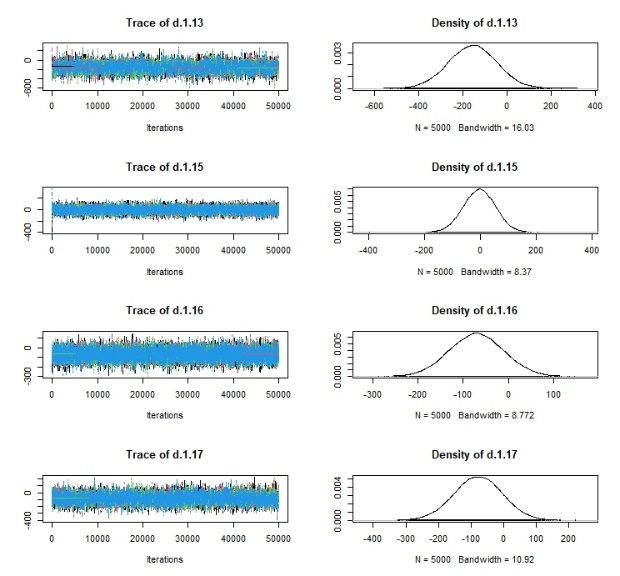

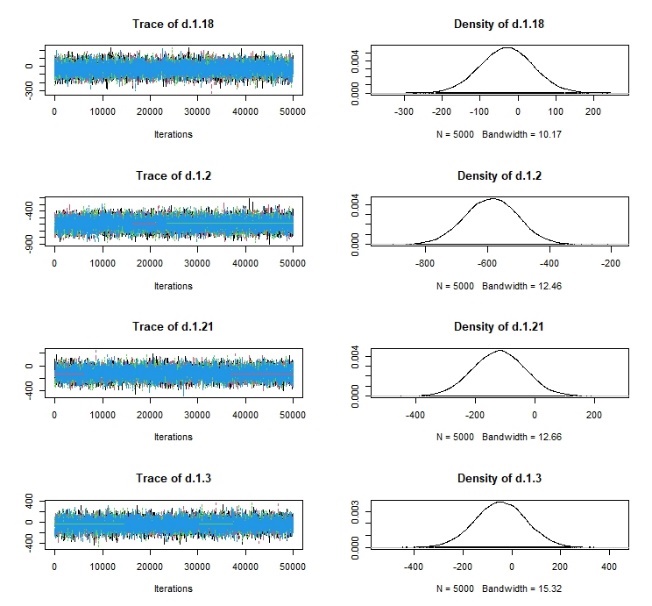
***

***
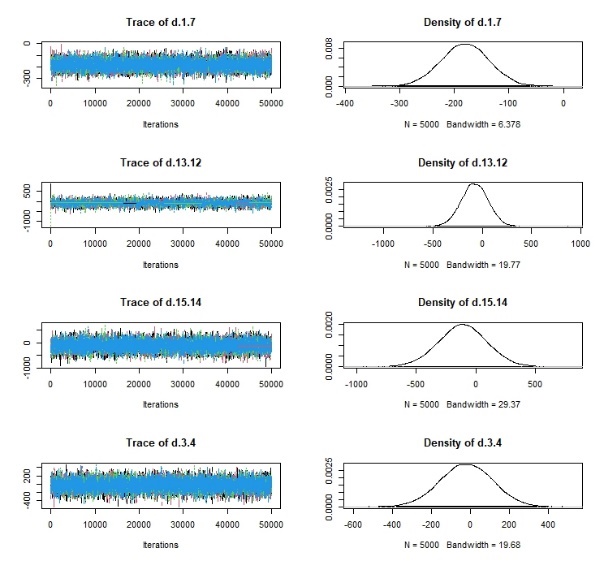

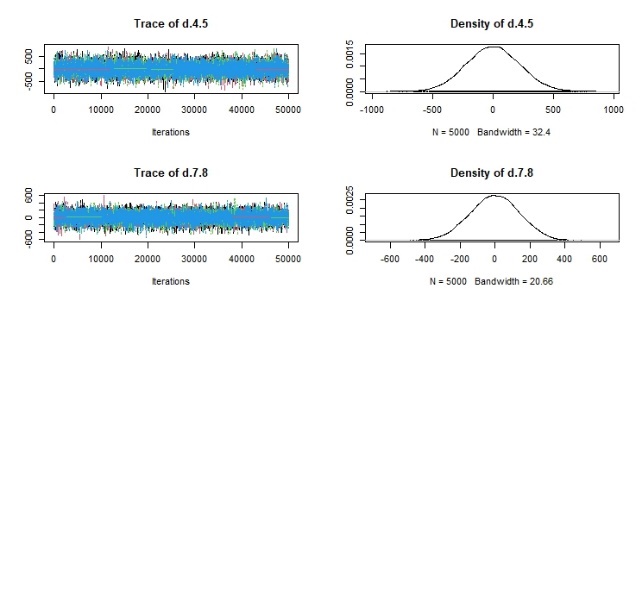
***

***MACROSOMIA***

***
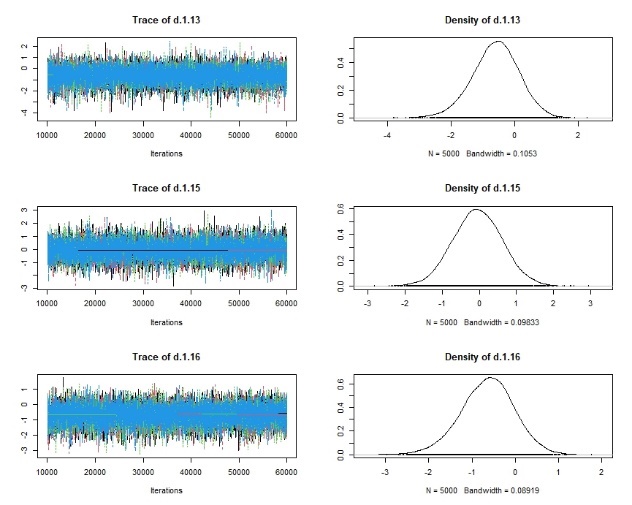

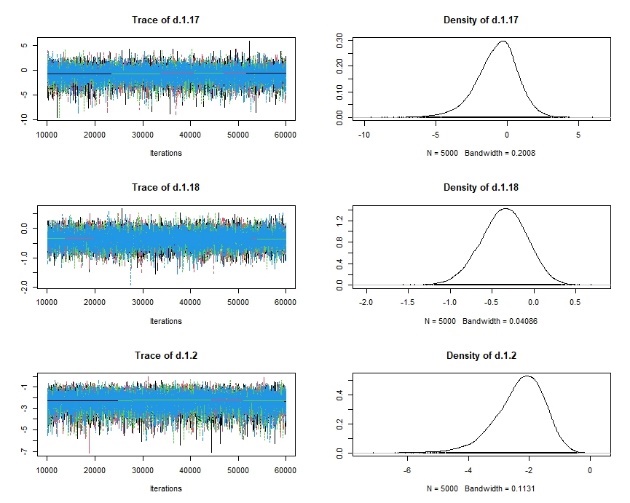
***

***
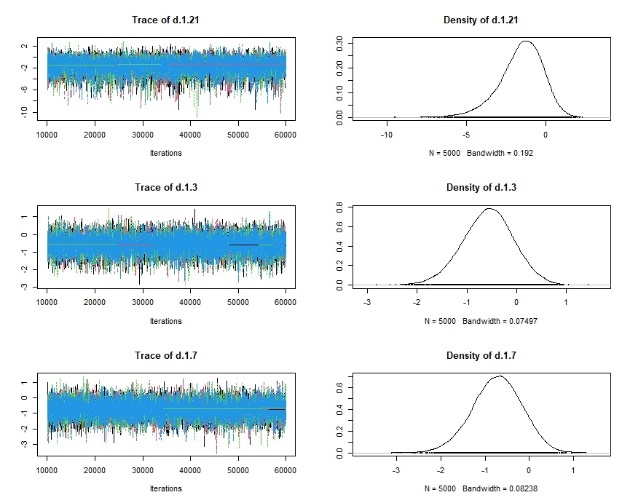

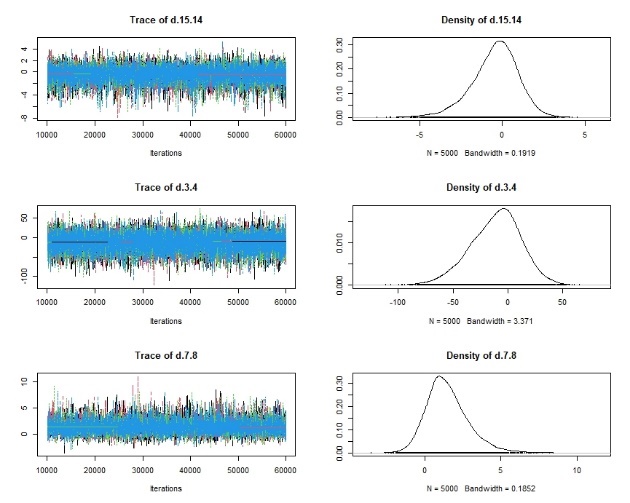
***

***PRETERM BIRTH***

***
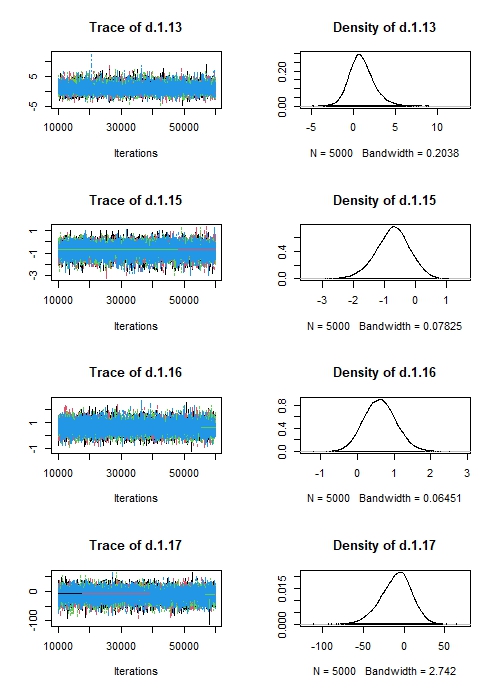

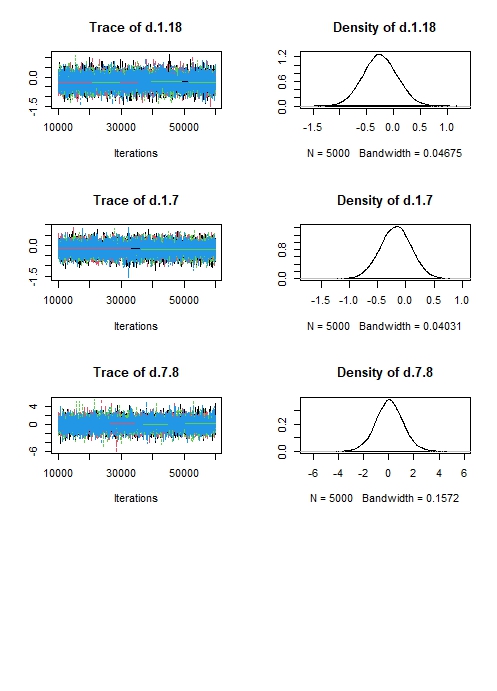
***

***Supplementary S17: Brooks-Gelman-Rubin Plots***

*1. Control; 2. DASH; 3. Low Carbohydrates; 4. High Carbohydrates; 5. High MUFA; 6. High GI/GL; 7. Low GI/GL; 8. High Fiber; 9. Energy Restriction; 12. High Complex Carbohydrates; 13. Soy Protein; 14. PMW; 15. Aerobic Exercise; 16. Resistance Exercise; 17. Higher Fiber/Lower Carbohydrates; 18. Structured Exercise; 20. Low GI + Yoga; 21. Aerobic + Resistance Exercise.*

***FASTING PLASMA GLUCOSE***

***
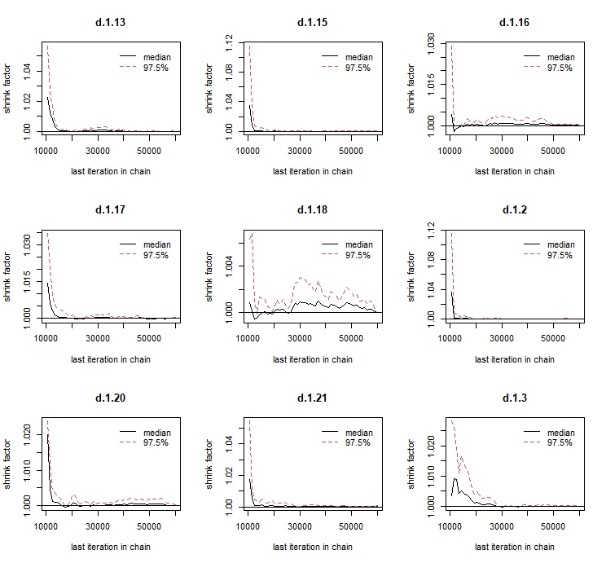

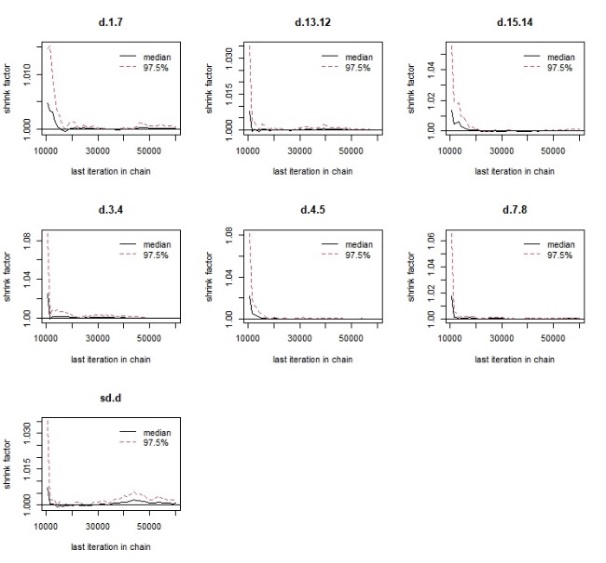
***

***2h POSTPRANDIAL GLUCOSE***

***
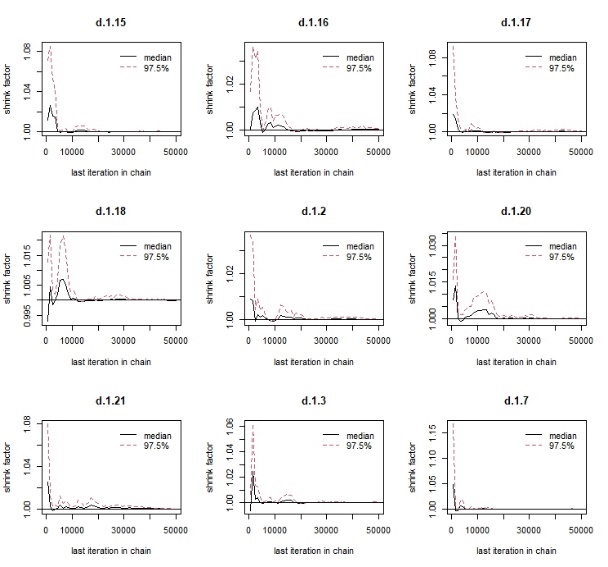

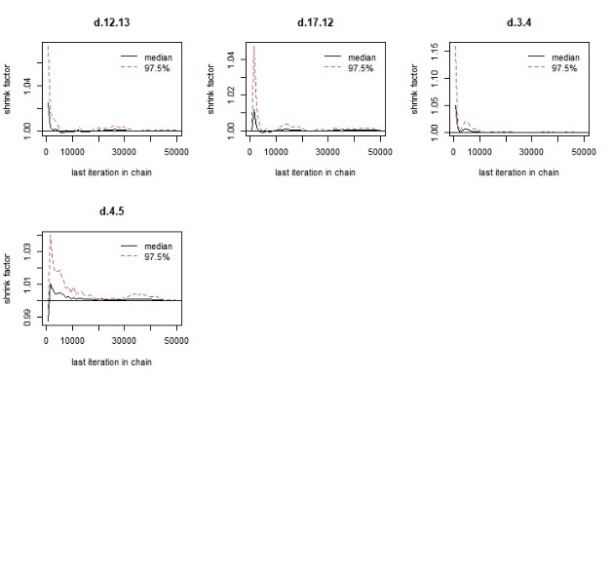
***

***INSULIN REQUIREMENTS***

***
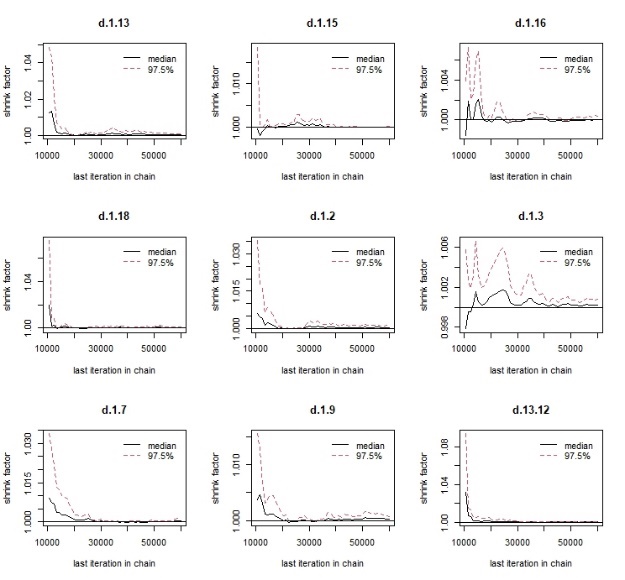

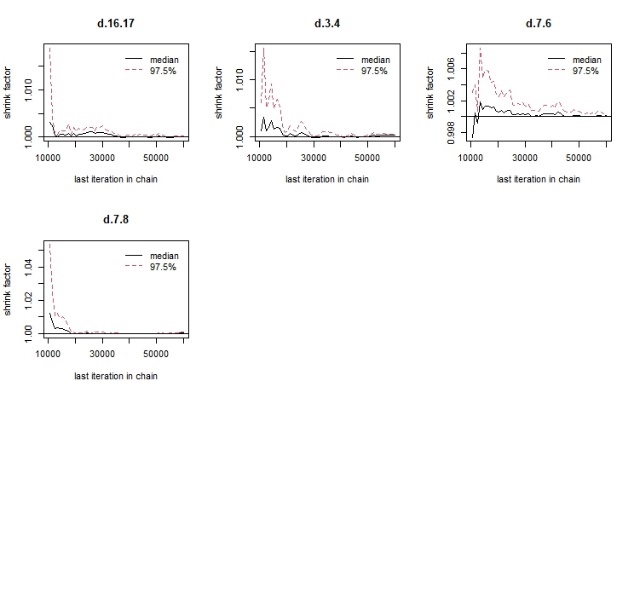
***

***BIRTH WEIGHT***

***
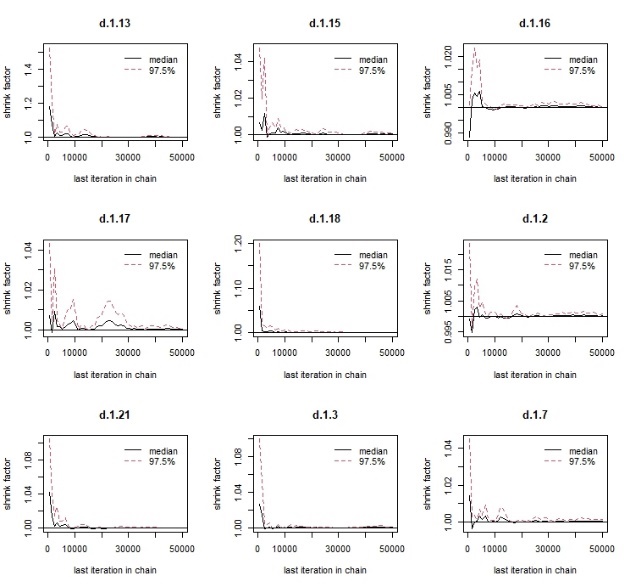

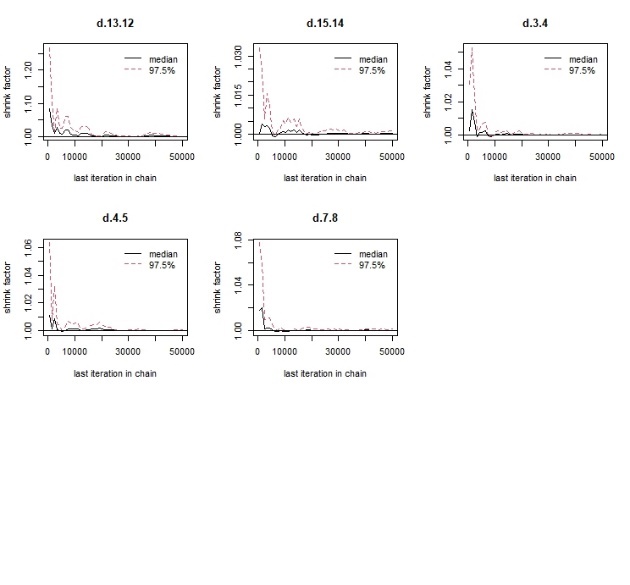
***

***MACROSOMIA***

***
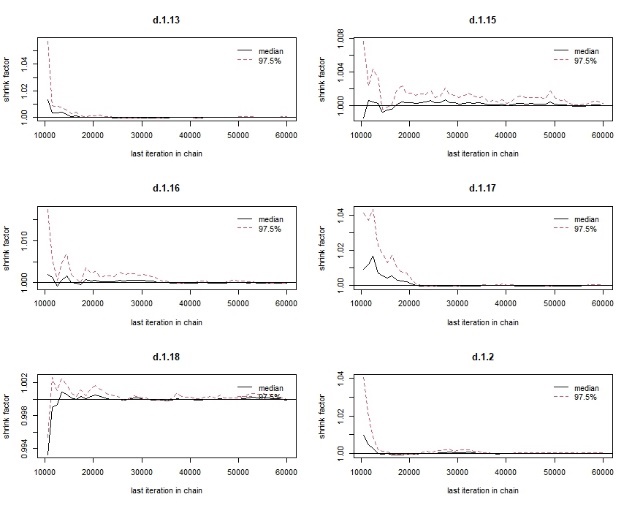

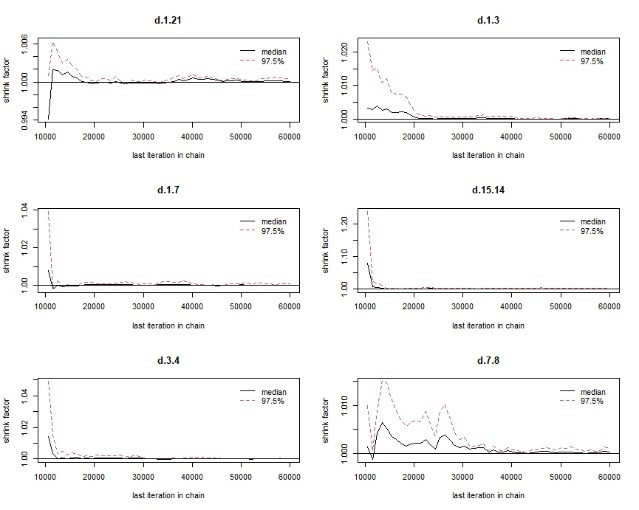
***

***PRETERM BIRTH***

***
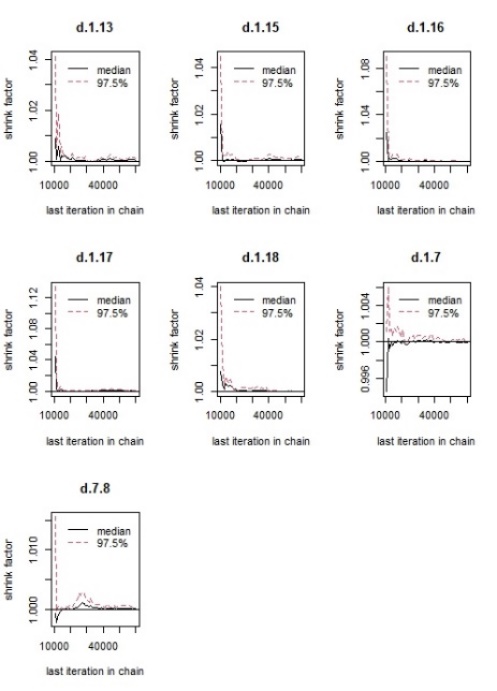
***

***Supplementary S18: Meta-analysis results of included data that cannot be used for network analysis.***

| Outcomes | Intervention | Comparison | N of studies | N of women | Effect estimate | I2(%) |
| --- | --- | --- | --- | --- | --- | --- |
| HOMA-IR | DASH | Control diet | 2 | 67 | -1.8 [-3.84, 0.24] | 91.2 |
|  | high complex carb | higher fat/lower carb | 3 | 112 | -0.41[-0.83, -0.0] | 0 |
|  | Low GI/GL | High fiber | 1 | 92 | -0.11[-0.52, 0.30] | - |
|  | Soy protein | Control diet | 1 | 68 | -0.79[-1.29, -0.30] | - |
| HbA1c (%) | DASH | Control diet | 1 | 34 | -0.98[-1.69, -0.27] | - |
|  | High MUFA | High carb | 1 | 25 | 0.22[-0.56, 1.01] | - |
|  | Low GI/GL | High fiber/control | 2 | 175 | 0.10[-0.20, 0.40] | 0 |
|  | aerobic | Usual care | 2 | 59 | -0.68[-2.13, 0.76] | 82.8 |
|  | Low carb | Control diet | 1 | 30 | -0.71[-1.45, 0.04] | - |
|  | structure | control | 1 | 131 | 0.18[-0.16, 0.52] | - |
|  | Soy protein | high complex carb | 1 | 62 | -0.05[-0.55, 0.45] | - |
| LGA | Low GI/GL | High fiber/control | 3 | 225 | 1.50[0.60, 3.73] | 0 |
|  | Low carb | Control diet | 2 | 193 | 0.46[0.13, 1.57] | 0 |
|  | structure | control | 1 | 90 | 1.31[0.52, 3.30] | - |
|  | Soy protein | high complex carb | 1 | 63 | 0.45[0.04, 4.76] | - |
|  | high complex carb | higher fat/lower carb | 1 | 46 | 0.75[0.19, 2.98] | - |
| CSR | DASH | Control diet | 3 | 119 | **0.54[0.40, 0.73]** | 0 |
|  | Low carb | Control/high carb | 4 | 292 | 1.00[0.73, 1.39] | 16.6 |
|  | high complex carb | higher fat/lower carb | 3 | 82 | 1.53[0.61, 3.83] | 29.8 |
|  | Structure | control | 5 | 442 | 0.88[0.68, 1.15] | 37.3 |
|  | aerobic | control | 3 | 158 | 0.78[0.51, 1.19] | 0 |
|  | aerobic + resistance | control | 2 | 163 | 0.45[0.18, 1.15] | 74 |
|  | resistance | control | 2 | 165 | 1.03[0.80, 1.34] | 0 |
|  | Soy protein | Control/ high complex carb | 2 | 130 | 0.93[0.57, 1.93] | 0 |
|  | Low GI/GL | High fiber | 1 | 88 | 0.57[0.21, 1.56] | - |
|  | Energy restriction | control | 1 | 121 | 1.18[0.74, 1.89] | - |
|  | aerobic | resistance | 1 | 86 | 1.31[0.80, 2.15] | - |

The data we extracted (HOMA-IR, HbA1c, Large for gestational age (LGA), and Cesarean Section Rate (CSR)) were not connected in a network, making it impossible to conduct a comprehensive network meta-analysis to evaluate the effects of various treatments across the entire network. Therefore, we performed a pairwise meta-analysis on these results. For studies with low heterogeneity (I² < 50%), we used a fixed-effects model for analysis. In contrast, for studies with high heterogeneity (I² ≥ 50%), we employed a random-effects model to account for the variability across studies.

***Supplementary S19: Inconsistency analysis***

*HF.LC.: Higher Fat/Lower Carbohydrates; HCC: High Complex Carbohydrates*

***FASTING PLASMA GLUCOSE***

***
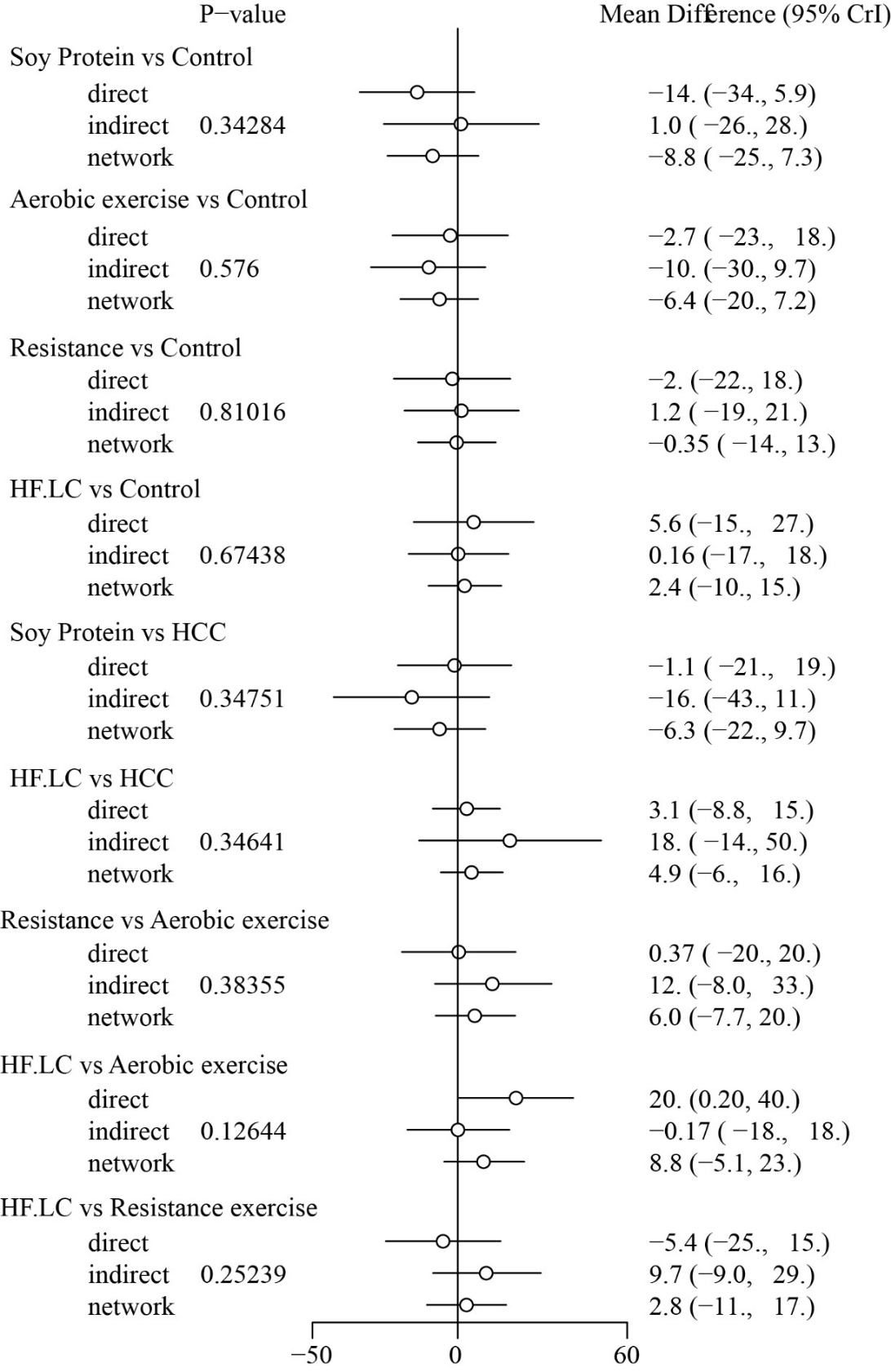
***

***2h POSTPRANDIAL GLUCOSE***

***
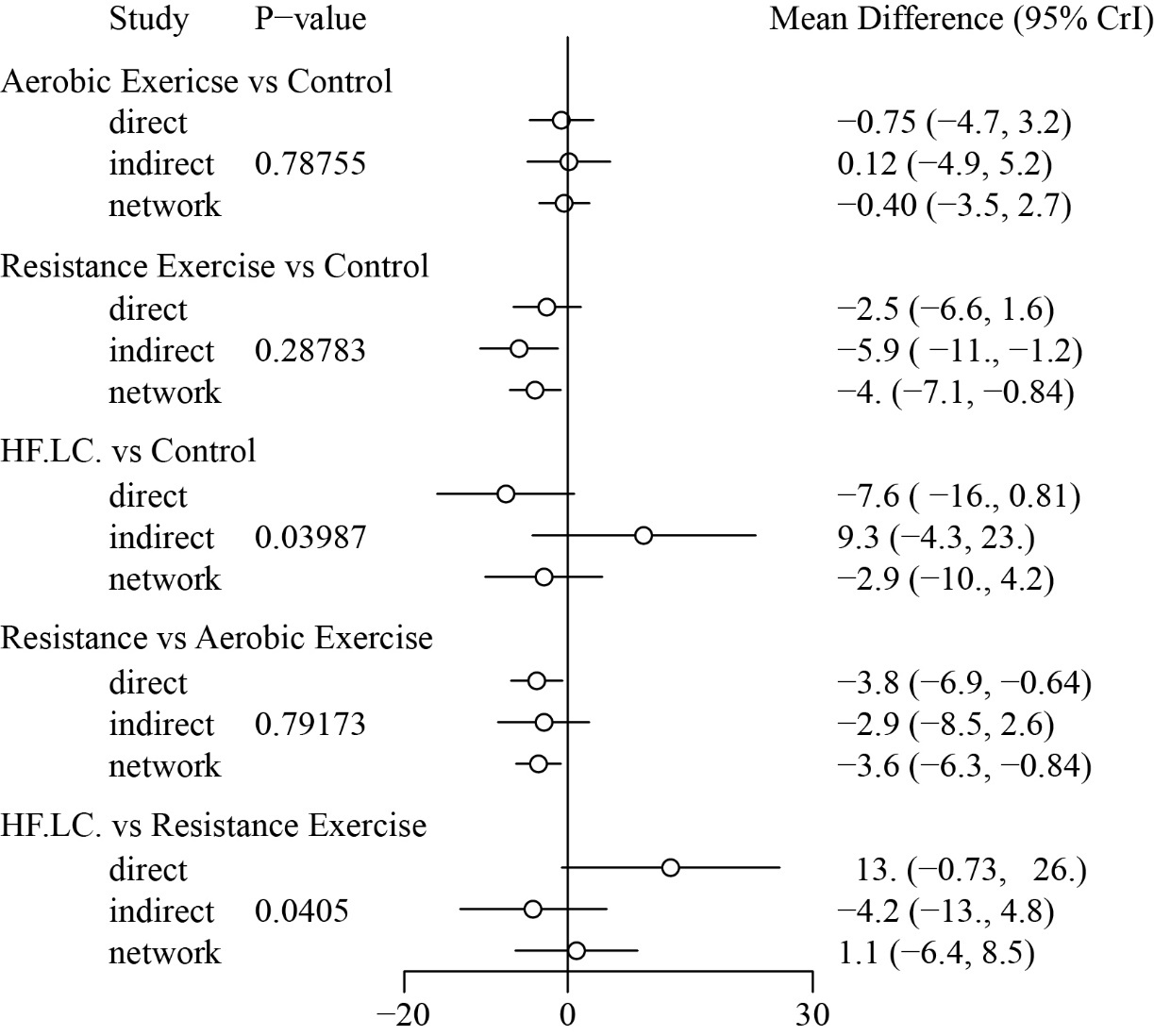
***

***INSULIN REQUIREMENTS***

***
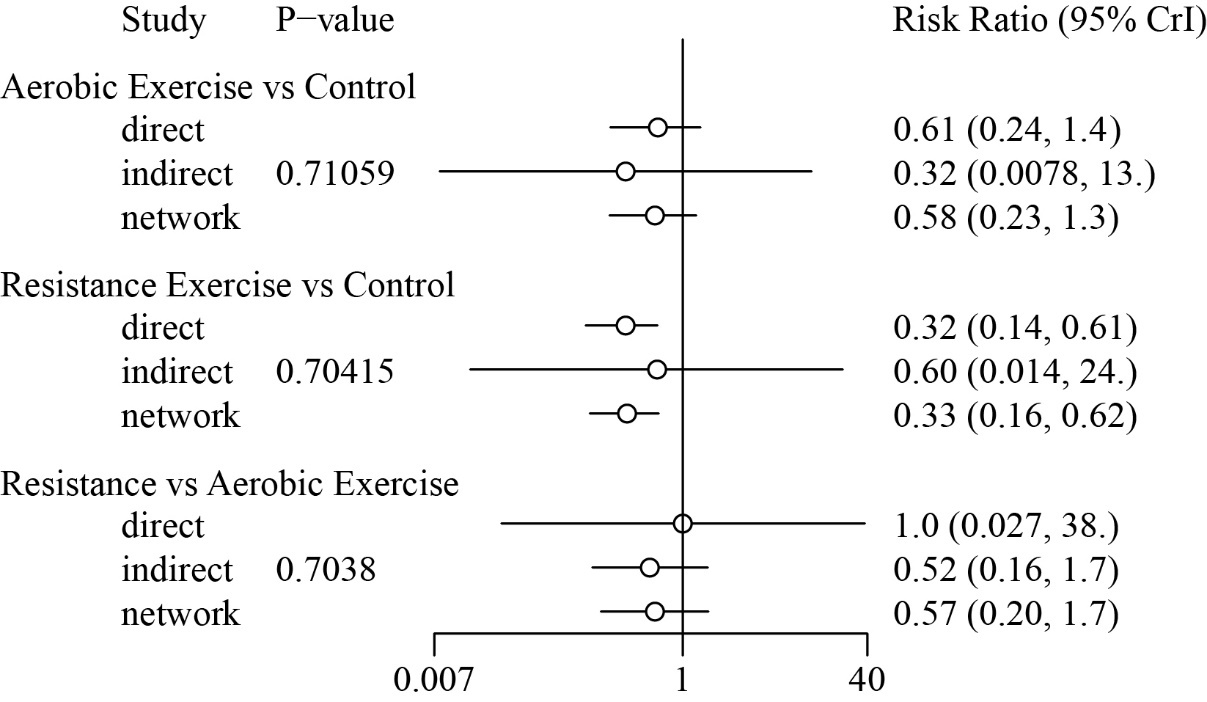
***

***BIRTH WEIGHT***

***
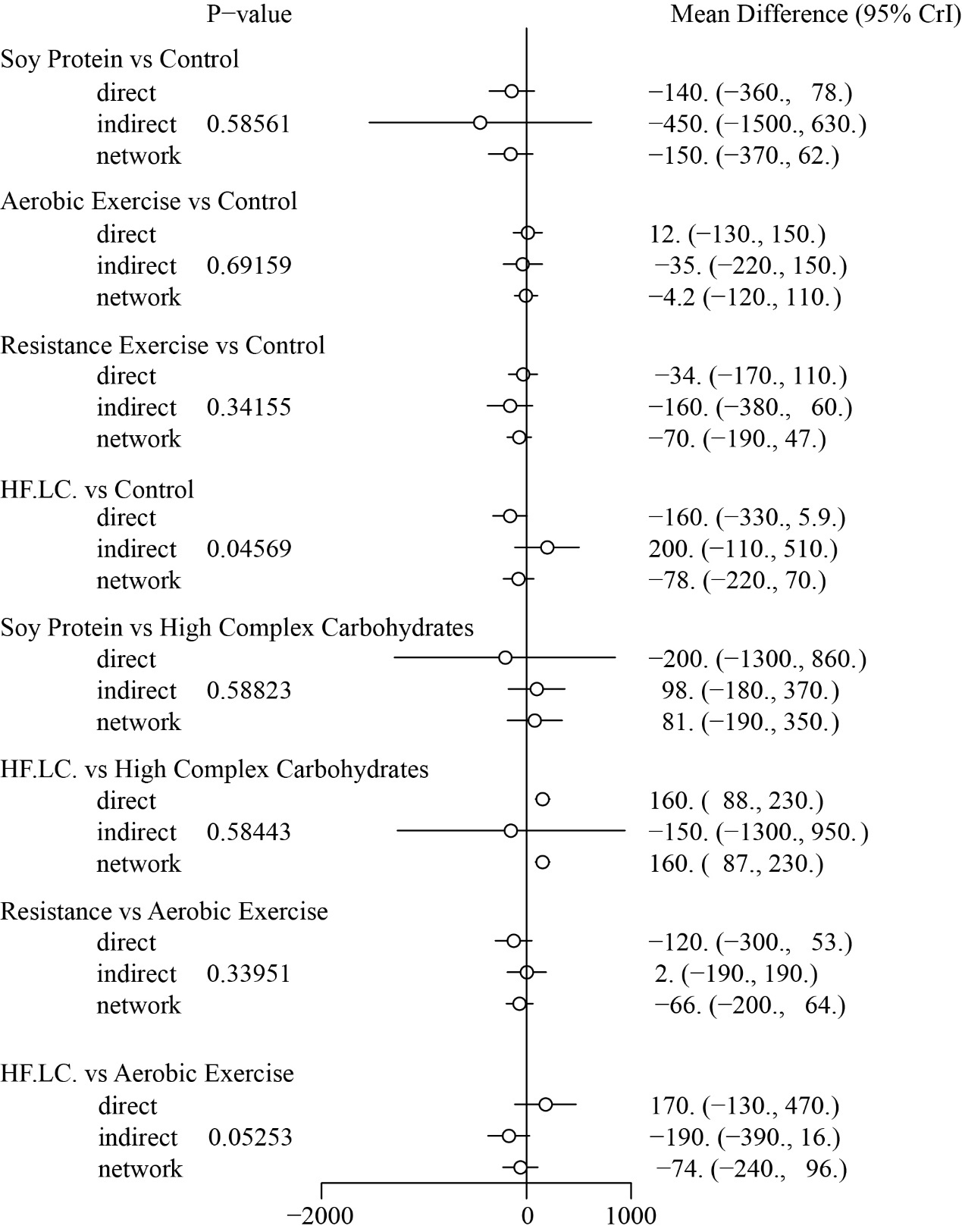
***

***MACROSOMIA***

***
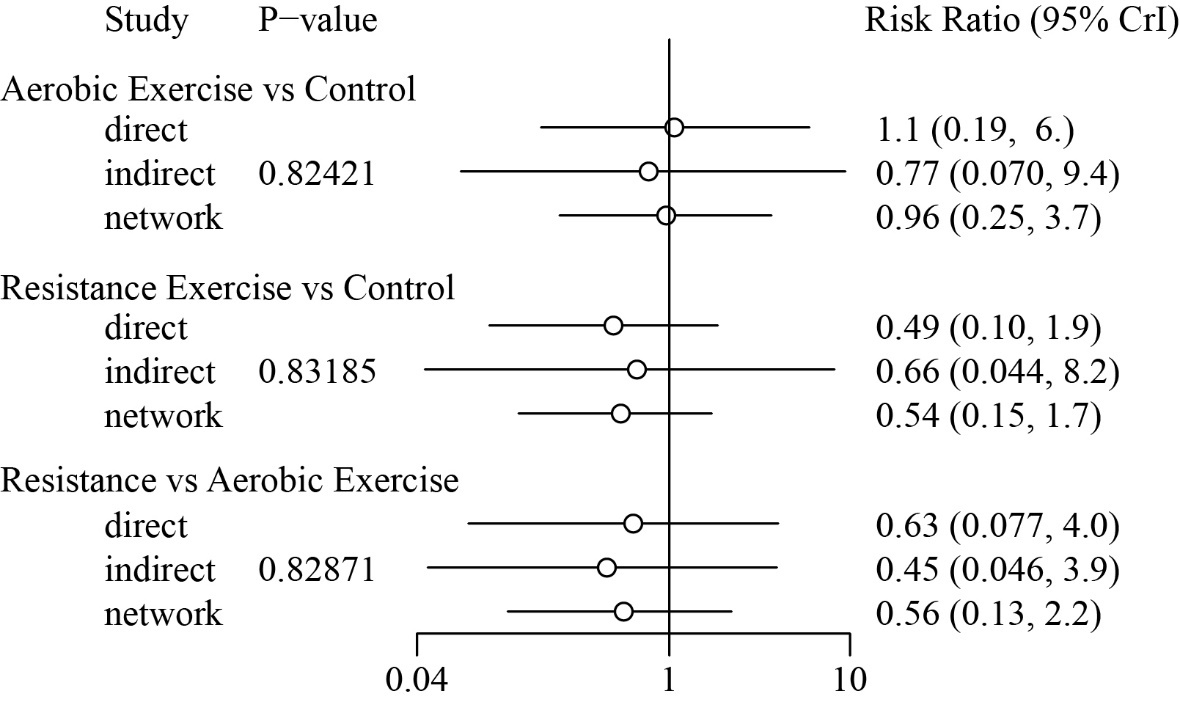
***

***PRETERM BIRTH***

***
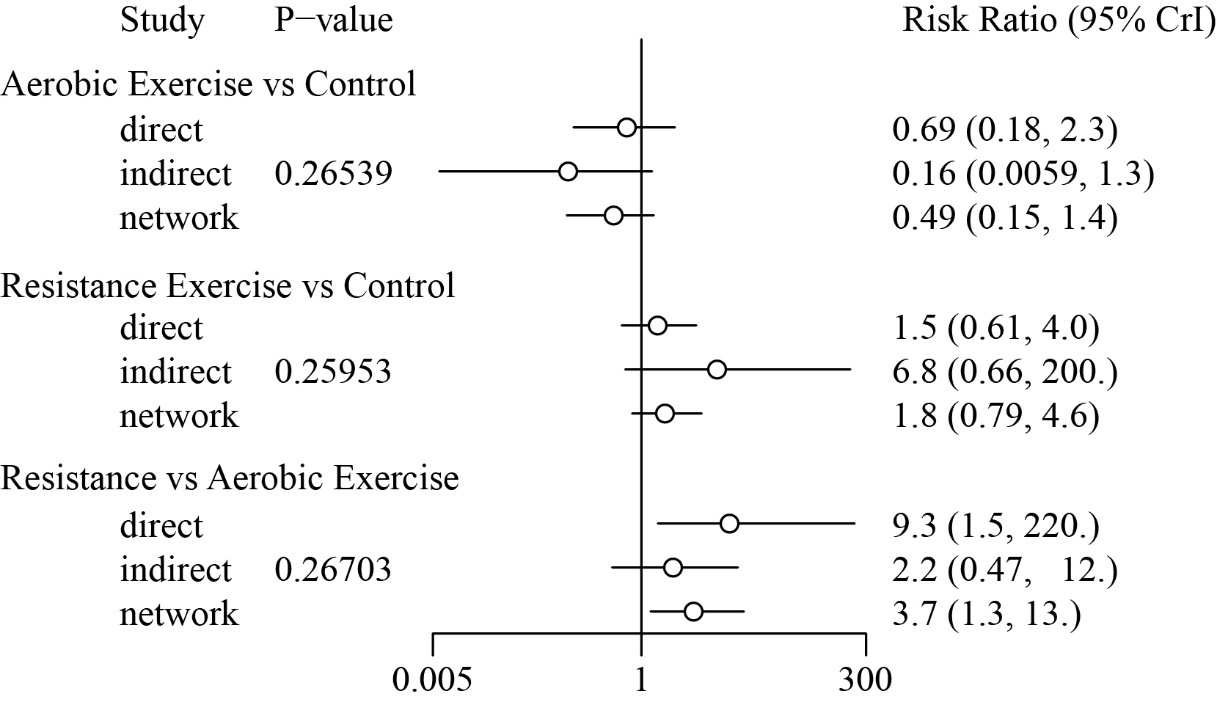
***

***Supplementary S20: Heterogeneity Analysis***

*HF.LC.: Higher Fat/Lower Carbohydrates; HCC: High Complex Carbohydrates*

***FASTING PLASMA GLUCOSE***

***
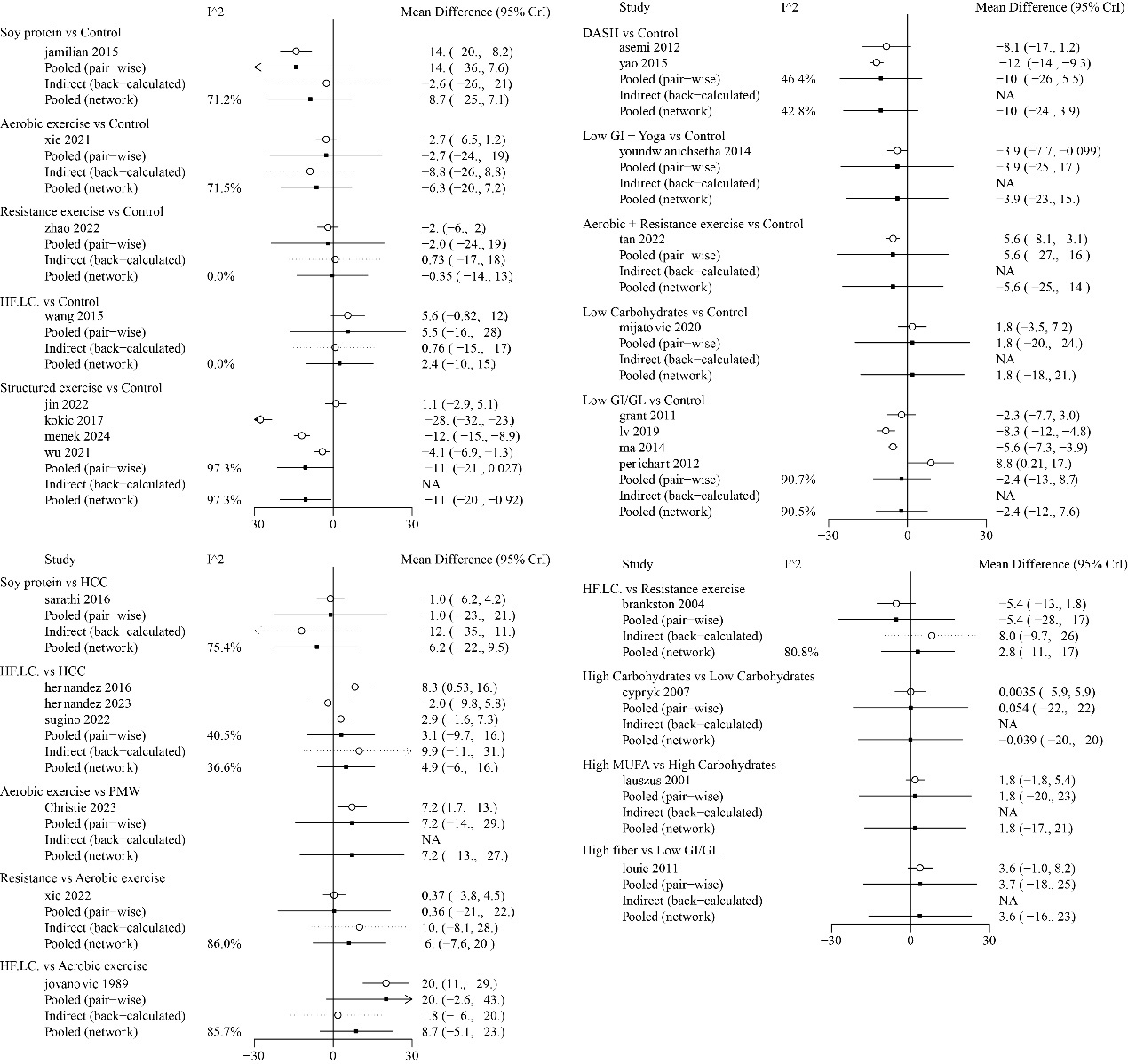
***

***2h POSTPRANDIAL GLUCOSE***


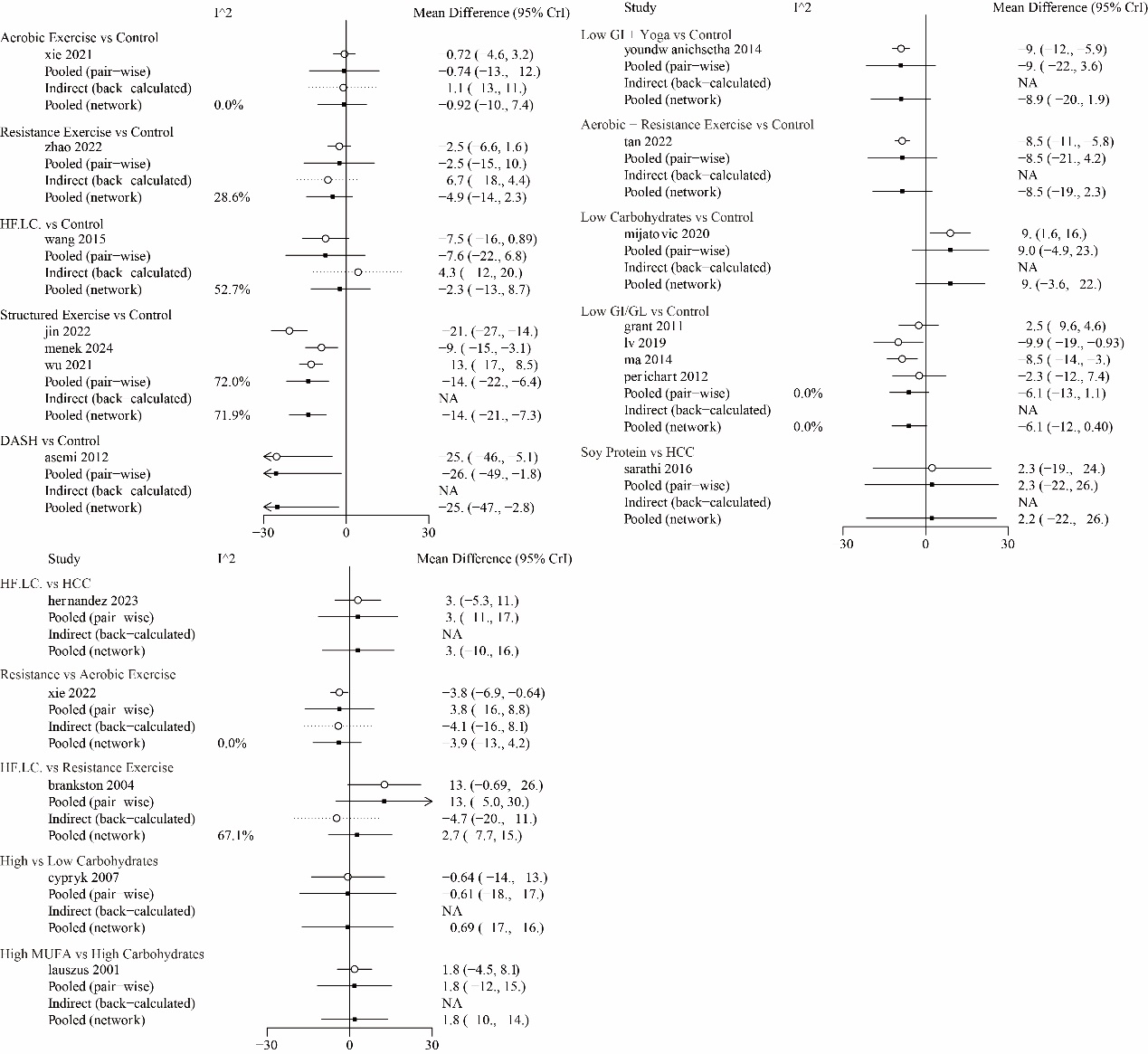


***INSULIN REQUIREMENTS***

***
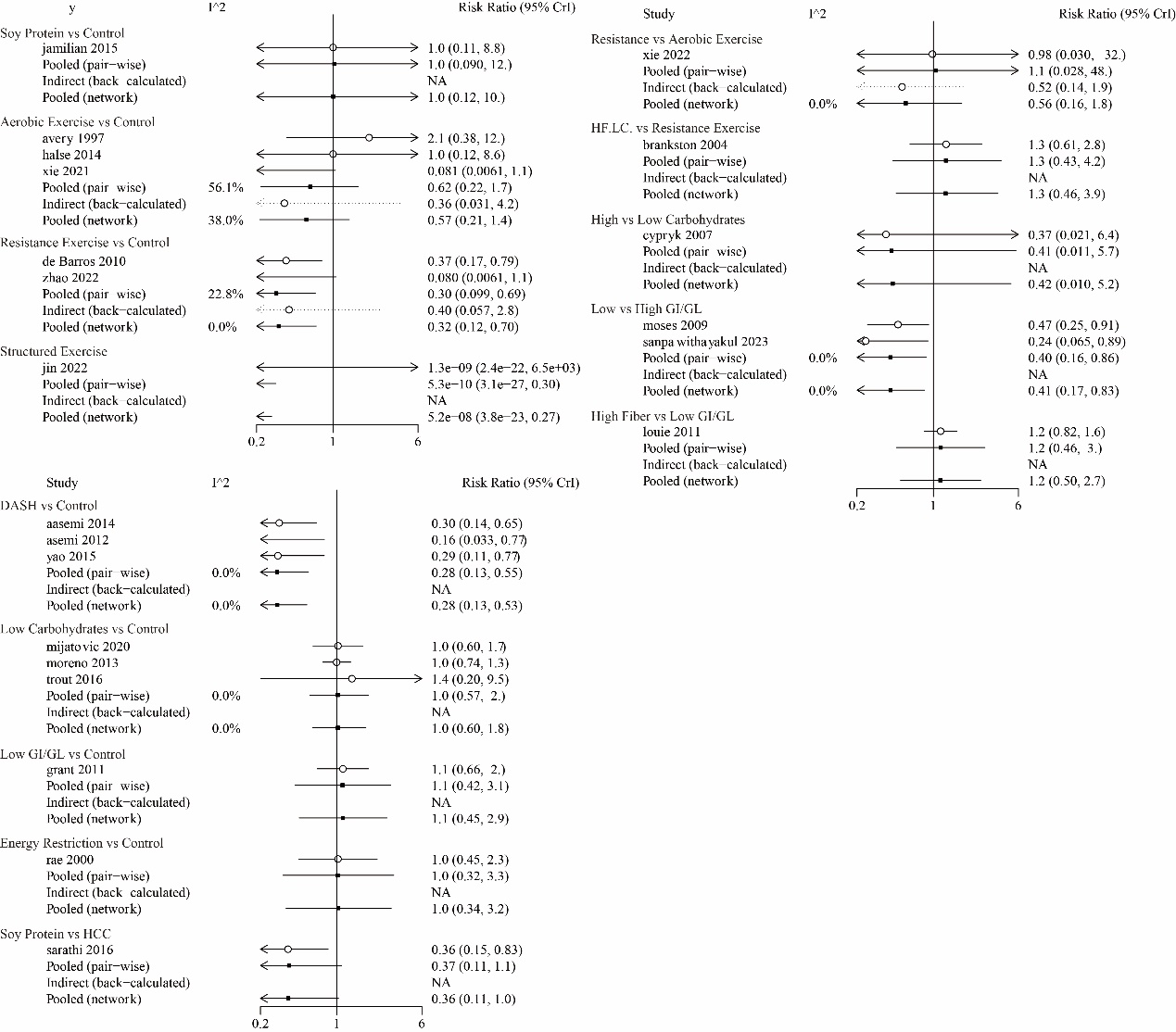
***

***BIRTH WEIGHT***

***
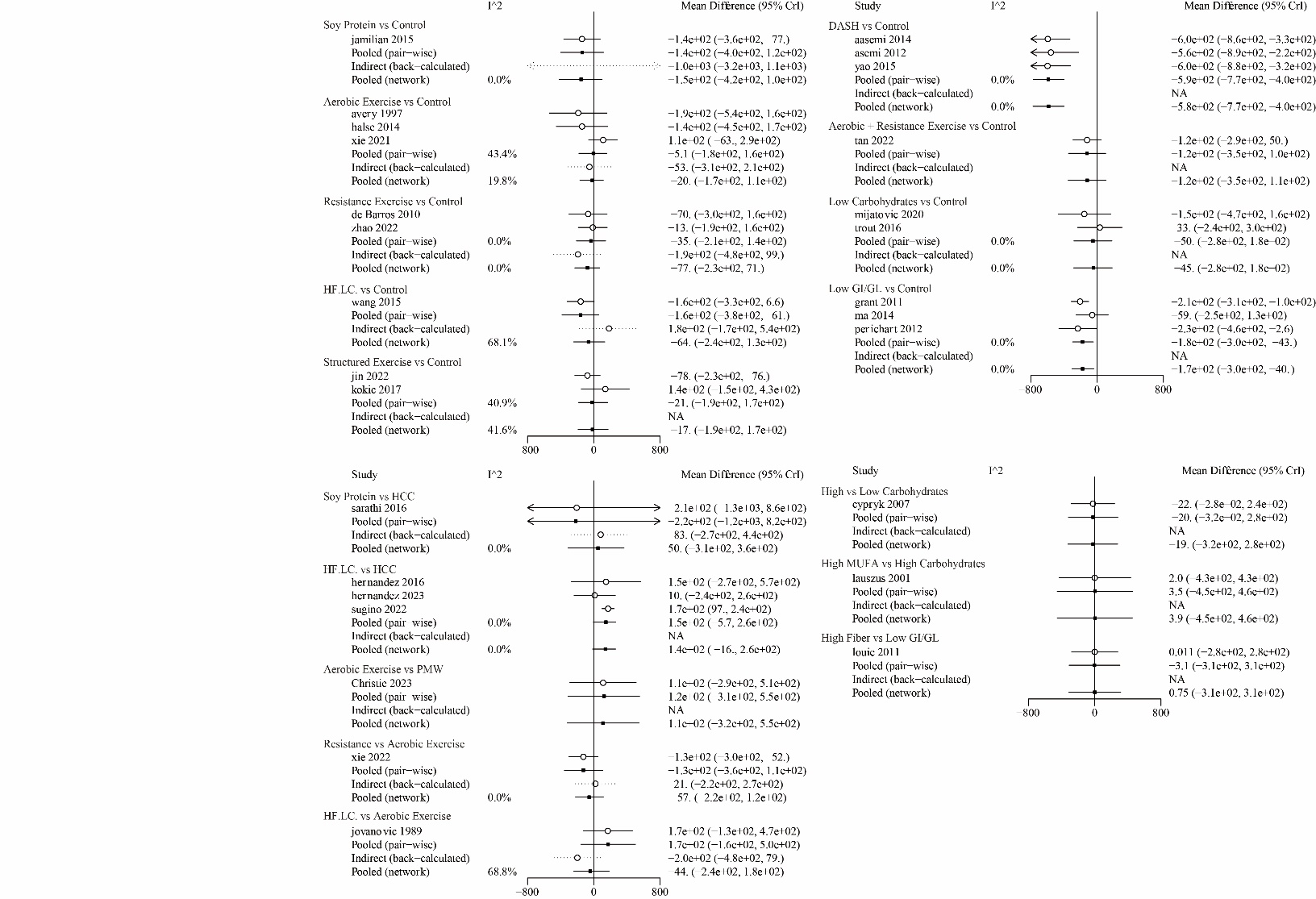
***

***MACROSOMIA***

***
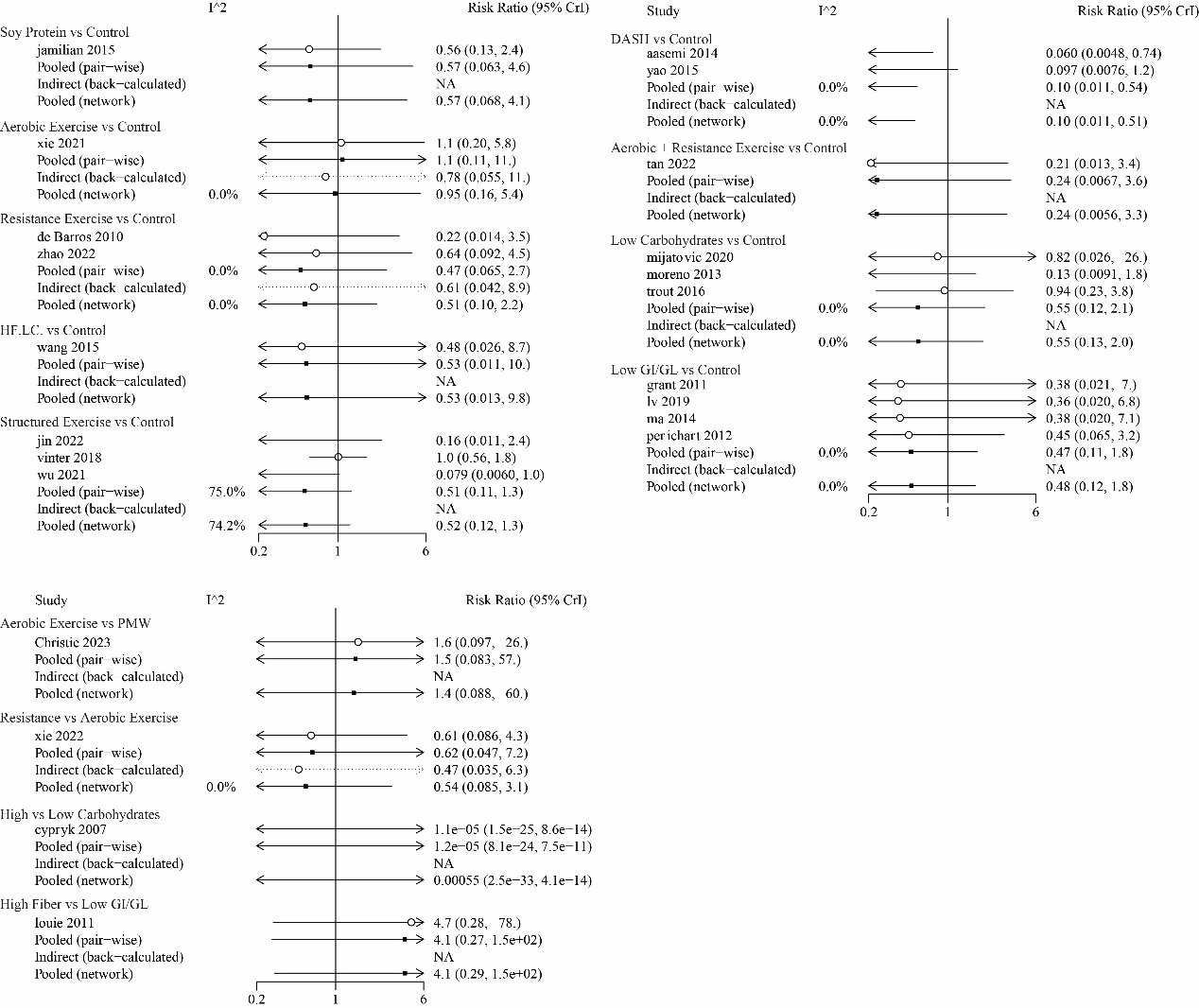
***

***PRETERM BIRTH***

***
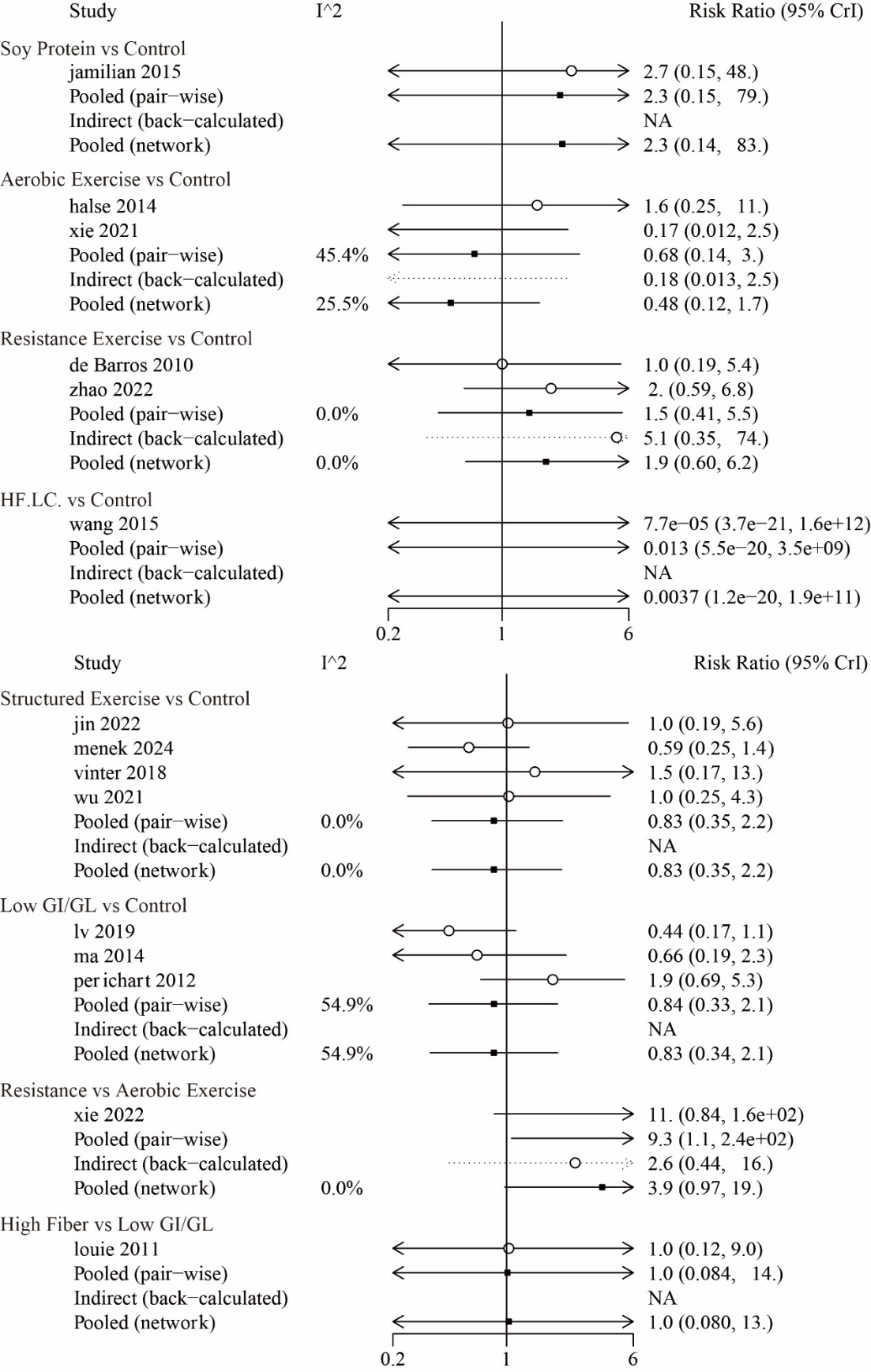
***

***Supplementary S21: Sensitivity analysis***

For FPG, local heterogeneity was detected in 8 comparisons involving 14 studies(1-14). We excluded these studies as a whole to observe the impact on network geometry and pooled estimates. Upon removal, the FPG evidence network disintegrated into a large loop and two independent studies unrelated to the loop (**Supplementary S14**). Since our analysis requires all evidence to be connected directly or indirectly in a closed network, these two independent studies had to be omitted, and sensitivity analysis was performed on the remaining large loop. Within this loop, the DASH intervention demonstrated superior effects compared to all other groups, and significant differences emerged across multiple group comparisons (**Supplementary S12**). In contrast, our original FPG network analysis only revealed a significant difference for the SE vs Control comparison. Regarding the ranking probabilities, HCC and HC exhibited a decrease after study removal, while other ranks remained relatively stable (**Supplementary S13**). These discrepancies potentially stemmed from the high heterogeneity across multiple studies, compounded by the network disconnection upon excluding highly heterogeneous evidence, as well as the missing data resulting from excluding the two disconnected studies, which may have partially contributed to the extreme uncertainty observed. These findings suggest insufficient robustness and questionable reliability of the current FPG network results.

Three comparisons (HFLC vs RE, HFLC vs Control, SE vs Control) involving 5 studies(3, 5, 6, 14, 15) exhibited high heterogeneity for the 2hPPG outcome, with one study(14) also displaying significant local inconsistency. After excluding these studies, the evidence network became disconnected (**Supplementary S14**). This disconnection could be partially attributed to the observed local inconsistency and heterogeneity within the loop of HFLC vs Control vs RE (**Supplementary S19 & S20**), exacerbated by the relatively small number of studies (only one study per comparison) informing this loop. Consequently, to mitigate the impact of these issues and maintain a relatively consistent network, we divided the 2hPPG network into two sub-networks and performed sensitivity analyses on the data within each sub-network separately.

For the small loop, which included 2 comparisons and 2 studies [HCC vs SOY(7) and HCC vs HFLC(16)], it is noteworthy that despite the limited comparisons and studies in the small loop's evidence network, the sensitivity analysis yielded consistent results with the overall network analysis, indicating the robustness of the findings in the small loop (**Supplementary S12 & S13**).

The large loop encompassed 10 comparisons and 13 studies(2, 8, 10-13, 17-23). The sensitivity analysis demonstrated that the effect estimates and rankings in the large loop were consistent with the original overall network results, suggesting the robustness of our findings in 2hPPG network (**Supplementary S12 & S13**). However, since the large and small loops were analyzed independently, we could not directly obtain the relative effect estimates between the interventions in the large and small loops.

For the Insulin requirement outcome, since zero events occurred in the SE group, rendering our data unanalyzable, we excluded this study(3) (SE vs Control; 0/65 vs 2/66) and performed the network meta-analysis again. After removing this study, there were no changes in the network results, rankings, inconsistency, or heterogeneity, indicating the robustness of our findings (**Supplementary S12 & S13**).

For the outcomes of preterm birth and macrosomia, we encountered zero events [HFLC vs Control (15), 0/41 vs 0/43; Low carb vs High carb (18), 0/15 vs 0/15, respectively] and significant local heterogeneity (LG vs Control, I^2^=54.9% and SE vs Control, I^2^=74.2%), respectively) (**Supplementary S20**). We performed sensitivity analyses by separately removing studies with zero events and heterogeneity. After excluding zero event studies, the effect estimates, heterogeneity statistics, inconsistency, and rankings for preterm and macrosomia remained unchanged (**Supplementary S12 & S13**), suggesting that the network meta-analysis results were stable, and zero events had a limited impact on the overall network.

Next, we proceeded to exclude studies exhibiting high heterogeneity. For the macrosomia outcome, after removing the highly heterogeneous studies, the effect estimates and rankings remained essentially unchanged (**Supplementary S12 & S13**). This suggests that the 3 studies(3, 6, 24) comparing SE and control were the primary contributors to local network heterogeneity for macrosomia, and our findings demonstrated robust stability. However, for preterm birth, exclusion of the highly heterogeneous studies led to network disconnection. Consequently, we were compelled to further omit the disconnected portion of evidence(25) that could not be analyzed (**Supplementary S14**). After excluding these studies, the effect estimates and rankings for preterm birth still did not exhibit significant changes, indicating good robustness of our results (**Supplementary S12 & S13**). Nevertheless, since we had to exclude one study that caused the network disconnection, this would inevitably lead to a reduction in our evidence base and may introduce bias. Further examination revealed that the excluded disconnected study compared LC vs HF interventions and reported similar preterm rates between the two groups (4.26% vs 4.08%, respectively). Although this suggests a potentially limited impact of this study on our network effect estimates and rankings, excluding this evidence may have reduced the generalizability of our conclusions.

Regarding birth weight, two comparisons(9, 15) exhibited local high heterogeneity, with Wang(15) et al. also displaying local inconsistency (**Supplementary S19 & S20**).

After excluding these two studies, several comparisons in our network showed significant changes (**Supplementary S12**). The comparisons of DASH vs. HCC, DASH vs. HFLC, HCC vs. AE, and HCC vs. Control became non-significant. The SUCRA rankings were also altered, with HCC dropping from the 2nd to the 10th position, HFLC shifting to 14th, and AE ranking the lowest. Other ranking changes were minor, with DASH remaining first and LG taking the second place (**Supplementary S13**)).

References:

1. Jamilian M, Asemi Z. The Effect of Soy Intake on Metabolic Profiles of Women With Gestational Diabetes Mellitus. *J Clin Endocrinol Metab*. 2015 Dec;100(12):4654-61.

2. Yaping X, Huifen Z, Meijing Z, Huibin H, Chunhong L, Fengfeng H et al. Effects of Moderate-Intensity Aerobic Exercise on Blood Glucose Levels and Pregnancy Outcomes in Patients With Gestational Diabetes Mellitus: A Randomized Controlled Trial. *Diabetes Ther*. 2021 Sep;12(9):2585-98.

3. Jin Y, Chen Z, Li J, Zhang W, Feng S. Effects of the original Gymnastics for Pregnant Women program on glycaemic control and delivery outcomes in women with gestational diabetes mellitus: A randomized controlled trial. *Int J Nurs Stud*. 2022 Aug;132:104271.

4. Sklempe Kokic I, Ivanisevic M, Biolo G, Simunic B, Kokic T, Pisot R. Combination of a structured aerobic and resistance exercise improves glycaemic control in pregnant women diagnosed with gestational diabetes mellitus. A randomised controlled trial. *Women Birth*. 2018 Aug;31(4):e232-e8.

5. Menek MY, Kaya AK. Comparison of home exercise under supervision and self home exercise in pregnant women with gestational diabetes: randomized controlled trial. *Arch Gynecol Obstet*. 2024 Mar;309(3):1075-82.

6. Wu Y, Xu M, Zheng G. Application of diversified and quantitative management model of exercise intervention in patients with gestational diabetes mellitus. *J Matern Fetal Neonatal Med*. 2022 Dec;35(25):5001-7.

7. Vijaya Sarathi AK, Hulivana Boranna Chaithanya, Chinthamani Suryanarayana Dwarakanath. Effect of Soya based Protein Rich Diet on Glycaemic Parameters and Thyroid Function Tests in Women with Gestational Diabetes Mellitus. *Romanian Journal of Diabetes Nutrition and Metabolic Diseases*. 2016;23(2):201-8.

8. Xie Y, Zhao H, Zhao M, Huang H, Liu C, Huang F et al. Effects of resistance exercise on blood glucose level and pregnancy outcome in patients with gestational diabetes mellitus: a randomized controlled trial. *BMJ Open Diabetes Res Care*. 2022 Apr;10(2).

9. Jovanovic-Peterson L, Durak EP, Peterson CM. Randomized trial of diet versus diet plus cardiovascular conditioning on glucose levels in gestational diabetes. *Am J Obstet Gynecol*. 1989 Aug;161(2):415-9.

10. Grant SM, Wolever TM, O'Connor DL, Nisenbaum R, Josse RG. Effect of a low glycaemic index diet on blood glucose in women with gestational hyperglycaemia. *Diabetes Res Clin Pract*. 2011 Jan;91(1):15-22.

11. Lv S, Yu S, Chi R, Wang D. Effects of nutritional nursing intervention based on glycemic load for patient with gestational diabetes mellitus. *Ginekol Pol*. 2019;90(1):46-9.

12. Ma WJ, Huang ZH, Huang BX, Qi BH, Zhang YJ, Xiao BX et al. Intensive low-glycaemic-load dietary intervention for the management of glycaemia and serum lipids among women with gestational diabetes: a randomized control trial. *Public Health Nutr*. 2015 Jun;18(8):1506-13.

13. Perichart-Perera O, Balas-Nakash M, Rodriguez-Cano A, Legorreta-Legorreta J, Parra-Covarrubias A, Vadillo-Ortega F. Low Glycemic Index Carbohydrates versus All Types of Carbohydrates for Treating Diabetes in Pregnancy: A Randomized Clinical Trial to Evaluate the Effect of Glycemic Control. *Int J Endocrinol*. 2012;2012:296017.

14. Brankston GN, Mitchell BF, Ryan EA, Okun NB. Resistance exercise decreases the need for insulin in overweight women with gestational diabetes mellitus. *Am J Obstet Gynecol*. 2004 Jan;190(1):188-93.

15. Wang H, Jiang H, Yang L, Zhang M. Impacts of dietary fat changes on pregnant women with gestational diabetes mellitus: a randomized controlled study. *Asia Pac J Clin Nutr*. 2015;24(1):58-64.

16. Hernandez TL, Farabi SS, Fosdick BK, Hirsch N, Dunn EZ, Rolloff K et al. Randomization to a Provided Higher-Complex-Carbohydrate Versus Conventional Diet in Gestational Diabetes Mellitus Results in Similar Newborn Adiposity. *Diabetes Care*. 2023 Nov 1;46(11):1931-40.

17. Asemi Z, Tabassi Z, Samimi M, Fahiminejad T, Esmaillzadeh A. Favourable effects of the Dietary Approaches to Stop Hypertension diet on glucose tolerance and lipid profiles in gestational diabetes: a randomised clinical trial. *Br J Nutr*. 2013 Jun;109(11):2024-30.

18. Cypryk K, Kaminska P, Kosinski M, Pertynska-Marczewska M, Lewinski A. A comparison of the effectiveness, tolerability and safety of high and low carbohydrate diets in women with gestational diabetes. *Endokrynol Pol*. 2007 Jul-Aug;58(4):314-9.

19. Lauszus FF, Rasmussen OW, Henriksen JE, Klebe JG, Jensen L, Lauszus KS et al. Effect of a high monounsaturated fatty acid diet on blood pressure and glucose metabolism in women with gestational diabetes mellitus. *Eur J Clin Nutr*. 2001 Jun;55(6):436-43.

20. Mijatovic J, Louie JCY, Buso MEC, Atkinson FS, Ross GP, Markovic TP et al. Effects of a modestly lower carbohydrate diet in gestational diabetes: a randomized controlled trial. *Am J Clin Nutr*. 2020 Aug 1;112(2):284-92.

21. Tan Y, Huang F, Wang Y, Lan Y, Cai S. Effects of Exercise Intervention Based on Self-efficacy Theory on Pregnant Women with Gestational Diabetes Mellitus. *Z Geburtshilfe Neonatol*. 2023 Jun;227(3):186-96.

22. Youngwanichsetha S, Phumdoung S, Ingkathawornwong T. The effects of mindfulness eating and yoga exercise on blood sugar levels of pregnant women with gestational diabetes mellitus. *Appl Nurs Res*. 2014 Nov;27(4):227-30.

23. Huifen Z, Yaping X, Meijing Z, Huibin H, Chunhong L, Fengfeng H et al. Effects of moderate-intensity resistance exercise on blood glucose and pregnancy outcome in patients with gestational diabetes mellitus: A randomized controlled trial. *J Diabetes Complications*. 2022 May;36(5):108186.

24. Vinter CA, Tanvig MH, Christensen MH, Ovesen PG, Jorgensen JS, Andersen MS et al. Lifestyle Intervention in Danish Obese Pregnant Women With Early Gestational Diabetes Mellitus According to WHO 2013 Criteria Does Not Change Pregnancy Outcomes: Results From the LiP (Lifestyle in Pregnancy) Study. *Diabetes Care*. 2018 Oct;41(10):2079-85.

25. Louie JC, Markovic TP, Perera N, Foote D, Petocz P, Ross GP et al. A randomized controlled trial investigating the effects of a low-glycemic index diet on pregnancy outcomes in gestational diabetes mellitus. *Diabetes Care*. 2011 Nov;34(11):2341-6.

36. Huifen Z, Yaping X, Meijing Z, Huibin H, Chunhong L, Fengfeng H, Yaping Z. Effects of moderate-intensity resistance exercise on blood glucose and pregnancy outcome in patients with gestational diabetes mellitus: A randomized controlled trial. J Diabetes Complications 2022;36:108186

37. Avery MD, Leon AS, Kopher RA. Effects of a partially home-based exercise program for women with gestational diabetes. Obstet Gynecol 1997;89:10-15

38. Hernandez TL, Farabi SS, Fosdick BK, Hirsch N, Dunn EZ, Rolloff K, Corbett JP, Haugen E, Marden T, Higgins J, Friedman JE, Barbour LA. Randomization to a Provided Higher-Complex-Carbohydrate Versus Conventional Diet in Gestational Diabetes Mellitus Results in Similar Newborn Adiposity. Diabetes Care 2023;46:1931-1940

39. Hernandez TL, Van Pelt RE, Anderson MA, Reece MS, Reynolds RM, de la Houssaye BA, Heerwagen M, Donahoo WT, Daniels LJ, Chartier-Logan C, Janssen RC, Friedman JE, Barbour LA. Women With Gestational Diabetes Mellitus Randomized to a Higher-Complex Carbohydrate/Low-Fat Diet Manifest Lower Adipose Tissue Insulin Resistance, Inflammation, Glucose, and Free Fatty Acids: A Pilot Study. Diabetes Care 2016;39:39-42

40. Jovanovic-Peterson L, Durak EP, Peterson CM. Randomized trial of diet versus diet plus cardiovascular conditioning on glucose levels in gestational diabetes. Am J Obstet Gynecol 1989;161:415-419

41. Sugino KY, Hernandez TL, Barbour LA, Kofonow JM, Frank DN, Friedman JE. A maternal higher-complex carbohydrate diet increases bifidobacteria and alters early life acquisition of the infant microbiome in women with gestational diabetes mellitus. Front Endocrinol (Lausanne) 2022;13:921464

42. Trout KK, Homko CJ, Wetzel-Effinger L, Mulla W, Mora R, McGrath J, Basel-Brown L, Arcamone A, Sami P, Makambi KH. Macronutrient Composition or Social Determinants? Impact on Infant Outcomes With Gestational Diabetes Mellitus. Diabetes Spectr 2016;29:71-78

43. Christie HE, Winter M, Meyer BJ, Francois ME. Diagnosis to Delivery: A Randomized Clinical Trial of Postmeal Walking in Women with Gestational Diabetes. Med Sci Sports Exerc 2024;56:860-867

44. Halse RE, Wallman KE, Dimmock JA, Newnham JP, Guelfi KJ. Home-Based Exercise Improves Fitness and Exercise Attitude and Intention in Women with GDM. Med Sci Sports Exerc 2015;47:1698-1704

45. Halse RE, Wallman KE, Newnham JP, Guelfi KJ. Home-based exercise training improves capillary glucose profile in women with gestational diabetes. Med Sci Sports Exerc 2014;46:1702-1709

46. Louie JC, Markovic TP, Perera N, Foote D, Petocz P, Ross GP, Brand-Miller JC. A randomized controlled trial investigating the effects of a low-glycemic index diet on pregnancy outcomes in gestational diabetes mellitus. Diabetes Care 2011;34:2341-2346

47. Mijatovic J, Louie JCY, Buso MEC, Atkinson FS, Ross GP, Markovic TP, Brand-Miller JC. Effects of a modestly lower carbohydrate diet in gestational diabetes: a randomized controlled trial. Am J Clin Nutr 2020;112:284-292

48. Moses RG, Barker M, Winter M, Petocz P, Brand-Miller JC. Can a low-glycemic index diet reduce the need for insulin in gestational diabetes mellitus? A randomized trial. Diabetes Care 2009;32:996-1000

49. Rae A, Bond D, Evans S, North F, Roberman B, Walters B. A randomised controlled trial of dietary energy restriction in the management of obese women with gestational diabetes. Aust N Z J Obstet Gynaecol 2000;40:416-422

50. Asemi Z, Samimi M, Tabassi Z, Sabihi SS, Esmaillzadeh A. A randomized controlled clinical trial investigating the effect of DASH diet on insulin resistance, inflammation, and oxidative stress in gestational diabetes. Nutrition 2013;29:619-624

51. Asemi Z, Tabassi Z, Samimi M, Fahiminejad T, Esmaillzadeh A. Favourable effects of the Dietary Approaches to Stop Hypertension diet on glucose tolerance and lipid profiles in gestational diabetes: a randomised clinical trial. Br J Nutr 2013;109:2024-2030

52. Asemi Z, Samimi M, Tabassi Z, Esmaillzadeh A. The effect of DASH diet on pregnancy outcomes in gestational diabetes: a randomized controlled clinical trial. Eur J Clin Nutr 2014;68:490-495

53. Jamilian M, Asemi Z. The Effect of Soy Intake on Metabolic Profiles of Women With Gestational Diabetes Mellitus. J Clin Endocrinol Metab 2015;100:4654-4661

54. Brankston GN, Mitchell BF, Ryan EA, Okun NB. Resistance exercise decreases the need for insulin in overweight women with gestational diabetes mellitus. Am J Obstet Gynecol 2004;190:188-193

55. Grant SM, Wolever TM, O'Connor DL, Nisenbaum R, Josse RG. Effect of a low glycaemic index diet on blood glucose in women with gestational hyperglycaemia. Diabetes Res Clin Pract 2011;91:15-22

56. Lauszus FF, Rasmussen OW, Henriksen JE, Klebe JG, Jensen L, Lauszus KS, Hermansen K. Effect of a high monounsaturated fatty acid diet on blood pressure and glucose metabolism in women with gestational diabetes mellitus. Eur J Clin Nutr 2001;55:436-443

57. Vinter CA, Tanvig MH, Christensen MH, Ovesen PG, Jorgensen JS, Andersen MS, McIntyre HD, Jensen DM. Lifestyle Intervention in Danish Obese Pregnant Women With Early Gestational Diabetes Mellitus According to WHO 2013 Criteria Does Not Change Pregnancy Outcomes: Results From the LiP (Lifestyle in Pregnancy) Study. Diabetes Care 2018;41:2079-2085

58. Sanpawithayakul K, Kaewprasert N, Tantiyavarong P, Wichansawakun S, Somprasit C, Tanathornkeerati N, Srichan C, Tharavanij T. Effects of the Consumption of Low to Medium Glycemic Index-based Rice on the Rate of Insulin Initiation in Patients with Gestational Diabetes: A Triple-blind, Randomized, Controlled Trial. Clin Ther 2023;45:347-353

59. Youngwanichsetha S, Phumdoung S, Ingkathawornwong T. The effects of mindfulness eating and yoga exercise on blood sugar levels of pregnant women with gestational diabetes mellitus. Appl Nurs Res 2014;27:227-230

60. Eman Awad HA, Amal Yousef, Ibtissam M. Saab. Eﬀect of antenatal exercise on mode of delivery in gestational diabetic females: a single-blind randomized controlled trial. Physiotherapy Quarterly 2019;27:1-5

61. Cypryk K, Kaminska P, Kosinski M, Pertynska-Marczewska M, Lewinski A. A comparison of the effectiveness, tolerability and safety of high and low carbohydrate diets in women with gestational diabetes. Endokrynol Pol 2007;58:314-319

62. de Barros MC, Lopes MA, Francisco RP, Sapienza AD, Zugaib M. Resistance exercise and glycemic control in women with gestational diabetes mellitus. Am J Obstet Gynecol 2010;203:556 e551-556

63. Sklempe Kokic I, Ivanisevic M, Biolo G, Simunic B, Kokic T, Pisot R. Combination of a structured aerobic and resistance exercise improves glycaemic control in pregnant women diagnosed with gestational diabetes mellitus. A randomised controlled trial. Women Birth 2018;31:e232-e238

64. Menek MY, Kaya AK. Comparison of home exercise under supervision and self home exercise in pregnant women with gestational diabetes: randomized controlled trial. Arch Gynecol Obstet 2024;309:1075-1082

65. Moreno-Castilla C, Hernandez M, Bergua M, Alvarez MC, Arce MA, Rodriguez K, Martinez-Alonso M, Iglesias M, Mateu M, Santos MD, Pacheco LR, Blasco Y, Martin E, Balsells N, Aranda N, Mauricio D. Low-carbohydrate diet for the treatment of gestational diabetes mellitus: a randomized controlled trial. Diabetes Care 2013;36:2233-2238

66. Perichart-Perera O, Balas-Nakash M, Rodriguez-Cano A, Legorreta-Legorreta J, Parra-Covarrubias A, Vadillo-Ortega F. Low Glycemic Index Carbohydrates versus All Types of Carbohydrates for Treating Diabetes in Pregnancy: A Randomized Clinical Trial to Evaluate the Effect of Glycemic Control. Int J Endocrinol 2012;2012:296017

67. Vijaya Sarathi AK, Hulivana Boranna Chaithanya, Chinthamani Suryanarayana Dwarakanath. Effect of Soya based Protein Rich Diet on Glycaemic Parameters and Thyroid Function Tests in Women with Gestational Diabetes Mellitus. Romanian Journal of Diabetes Nutrition and Metabolic Diseases 2016;23:201-208

***Supplementary S22: Meta-regression analysis***

***FASTING PLASMA GLUCOSE***


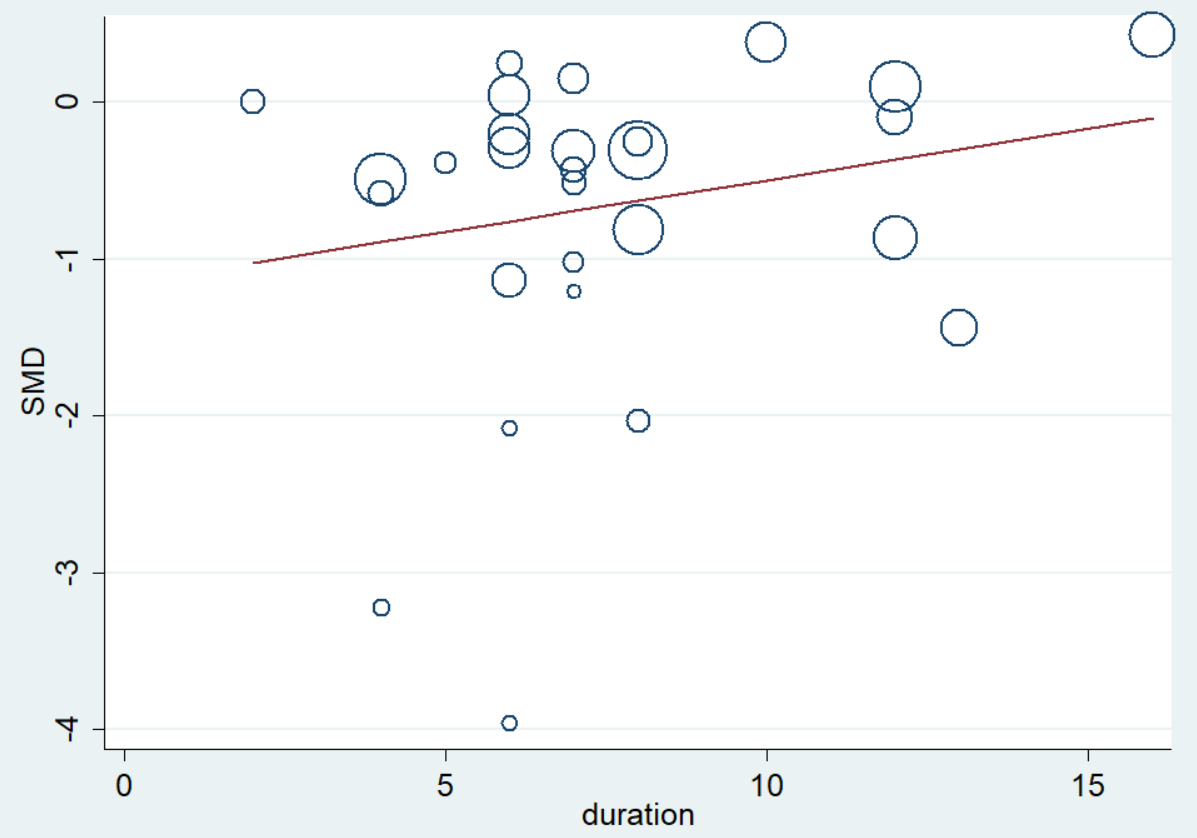


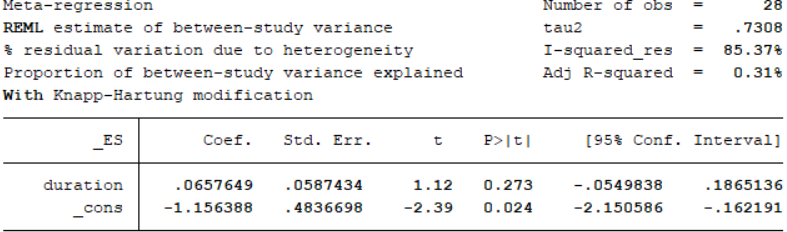


***2h POSTPRANDIAL GLUCOSE***


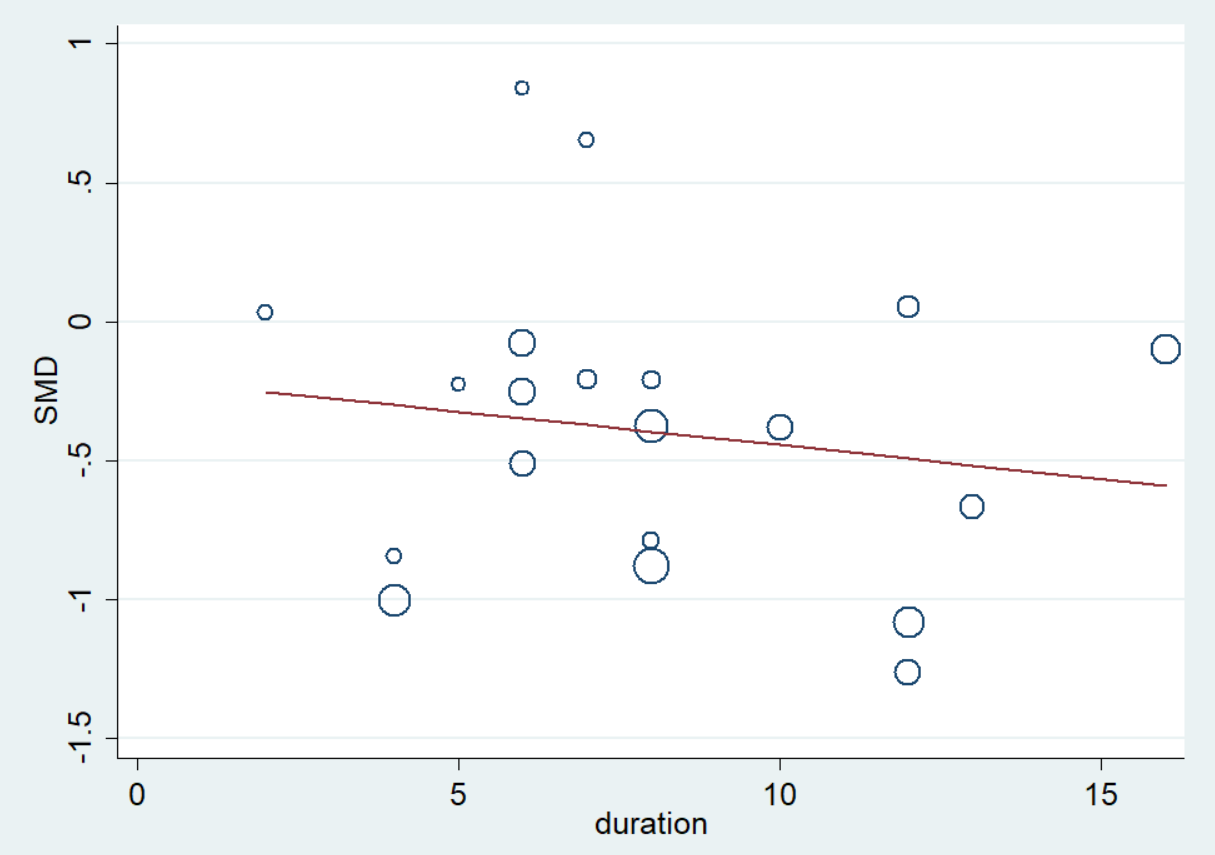


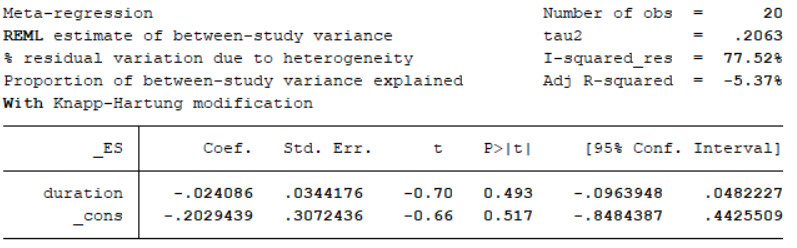


***INSULIN REQUIREMENTS***


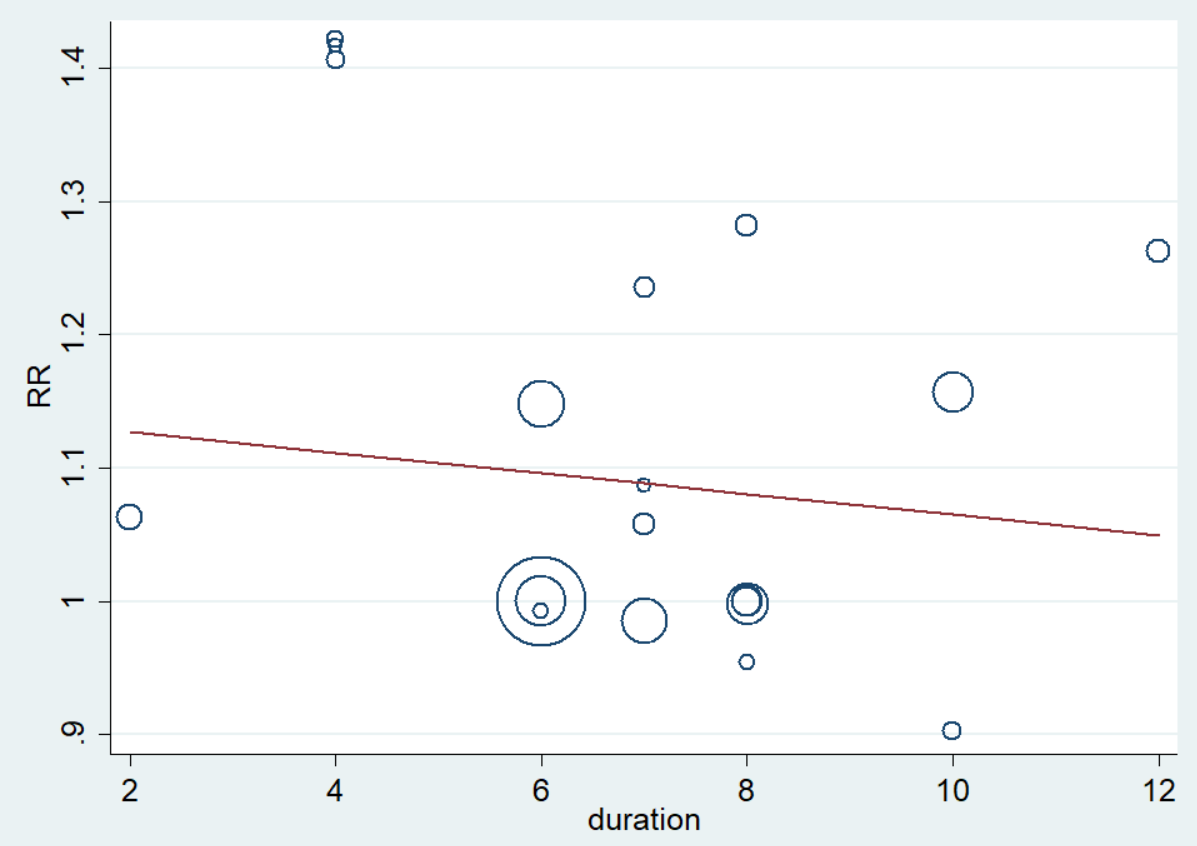


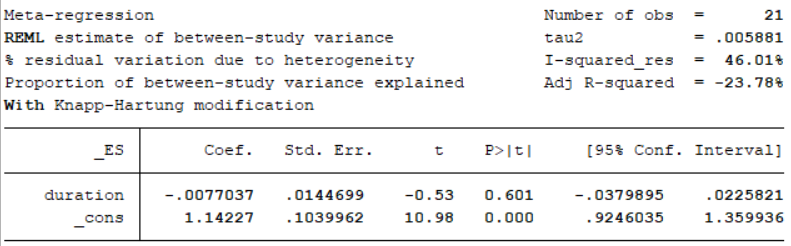


***BIRTH WEIGHT***


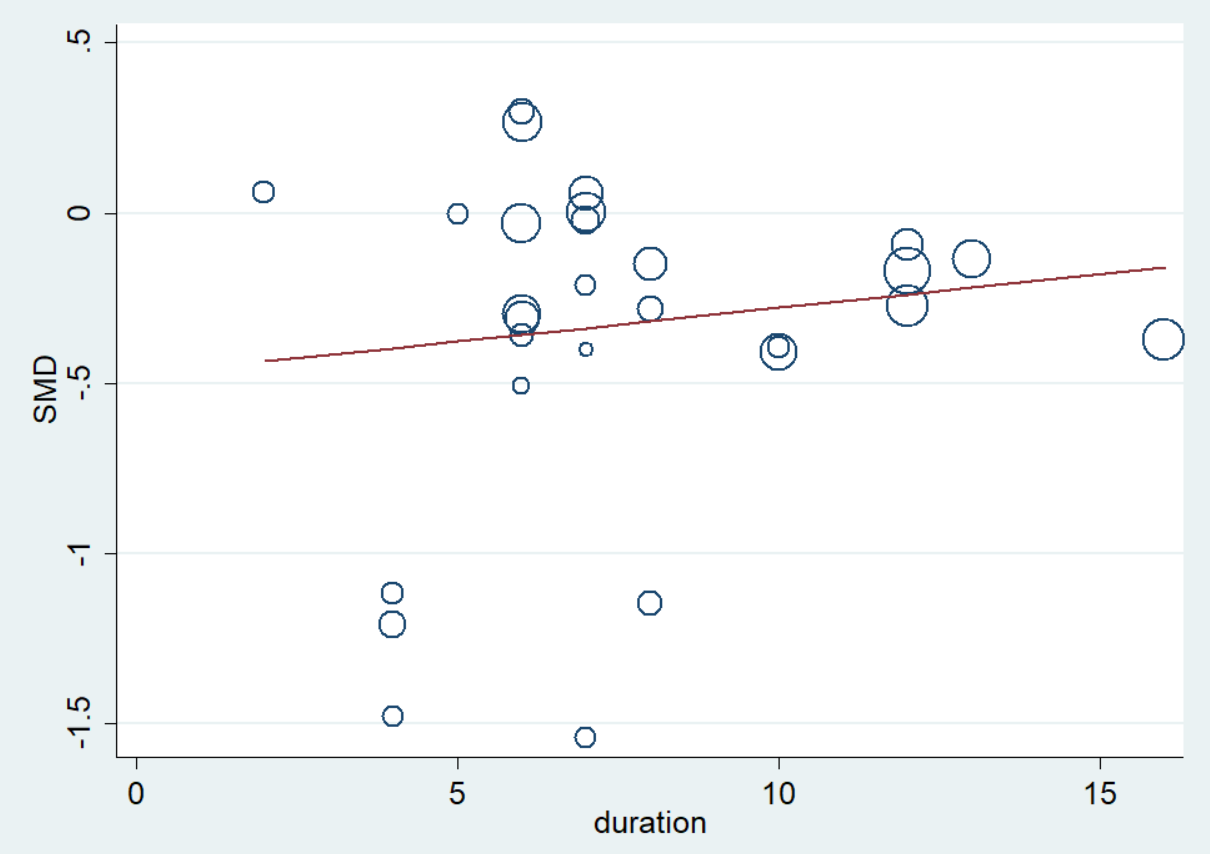


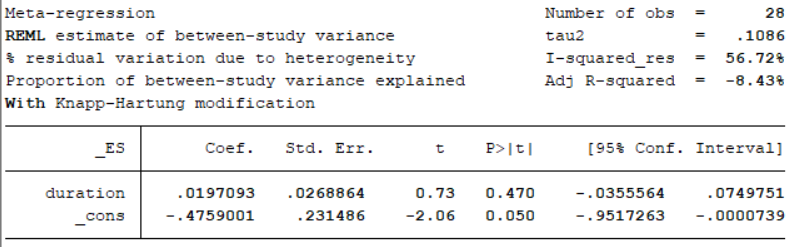


***MACROSOMIA***


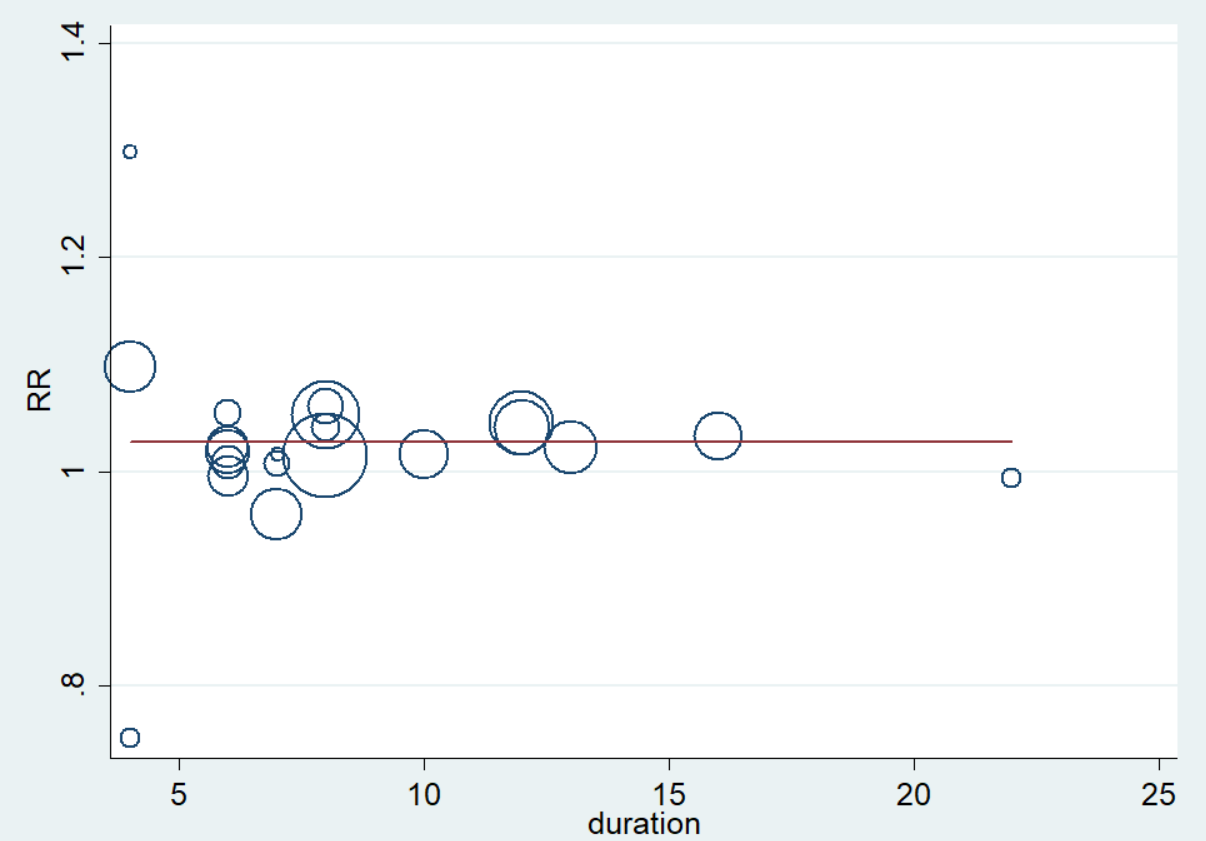


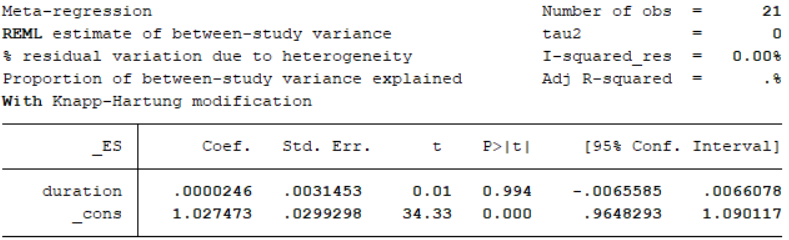


***PRETERM BIRTH***


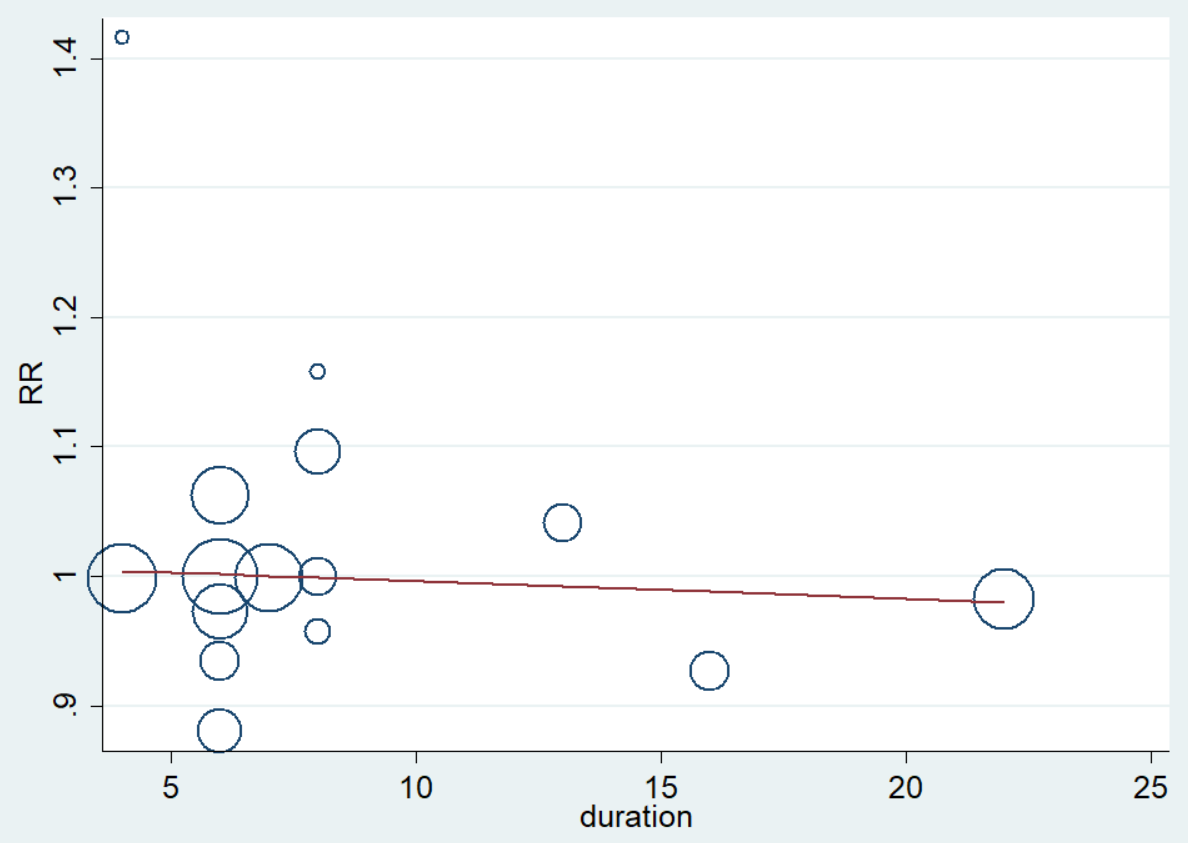


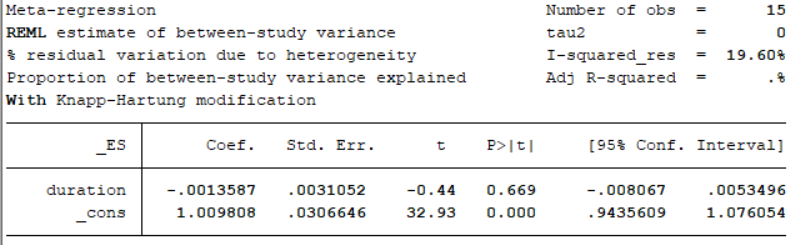

Supplement: Multimedia component 1 [file mmc1.docx]
